# Tailoring Liquid Crystalline Self‐Assembly and Photophysical Properties of Unsymmetrical Fluorenones via Polar Terminal Groups

**DOI:** 10.1002/chem.71542

**Published:** 2026-08-13

**Authors:** Falk Feucht, Franziska Müller, Johanna Osterbrink, Anna Zens, Yann Molard, Sabine Laschat

**Affiliations:** ^1^ Institut für Organische Chemie Universität Stuttgart Stuttgart Germany; ^2^ University of Rennes, CNRS, ISCR─UMR 6226, ScanMAT─UAR 2025 Rennes France

**Keywords:** fluorenone, fluorescence, liquid crystal, luminescence switching, smectic phase, solvatochromism

## Abstract

In this work, a series of novel unsymmetrical fluorenone derivatives bearing various head groups (H, Br, NO_2_, NH_2_, and CN) was synthesized and investigated with respect to their mesomorphic and photophysical properties. Depending on the head group, SmA, N, or SmC phases with phase widths of up to 145 K were observed, except for the NH_2_‐substituted derivative. All mesomorphic derivatives exhibited bright yellow to orange fluorescence in the solid state and solution with quantum yields of up to 35%. Notably, the nitrile derivative showed thermally induced color and emission color shift from yellow to orange, which was not reversible upon cooling to room temperature. These results revealed that the mesomorphic and photophysical properties of the examined fluorenone derivatives are strongly dependent on the headgroup and, in the latter case, can be significantly influenced by the presence and nature of solvent or temperature.

## Graphical abstract

Novel liquid crystalline fluorenones bearing various head groups were synthesized and characterized. By alteration of the head group, N, SmC, or SmA phases were obtained, and emission properties could be tuned depending on solvent or temperature.

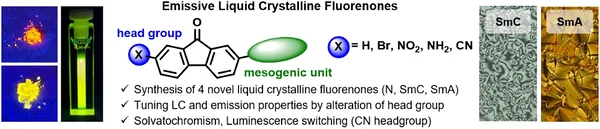

## 1 Introduction

Thermotropic rod‐like liquid crystals self‐assemble into mesophases resulting from nanosegregation of their immiscible parts, minimization of free volume, van der Waals interactions of aliphatic side chains, *π*−*π* interactions and/or H‐bonding. In addition, the role of polarity, that is, through polar groups attached to the mesogenic unit, on the formation and stabilization of liquid crystalline (LC) phases has recently received increasing attention [1, 2]. This is due to the discovery of polar and ferroelectric phases for non‐chiral LC compounds such as **RM734**  [3, 4] and **nX**  [1] (Scheme 1a), enabling applications in electrooptical devices [5]. Beyond materials displaying polar nematic phases, the attachment of polar terminal groups to the mesogenic core has been found beneficial for a large variety of different mesogens (Scheme 1a).

**SCHEME 1:**
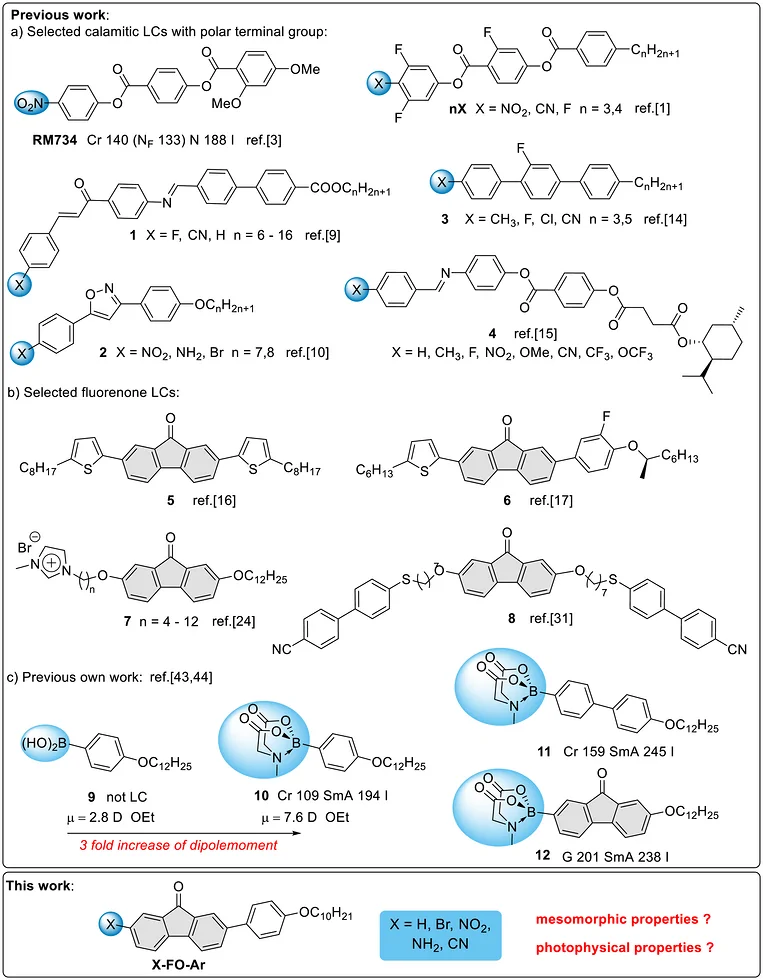
Previously reported (a) calamitic LCs with terminal groups, (b) fluorenone LCs, (c) own work and fluorenone target structures with various terminal groups of this work.

For example, the photo‐responsiveness of cholesteryl‐azobenzene dimers was affected by terminal groups [6, 7]. Quaterphenyls with low dielectric loss were obtained by terminal group manipulation [8]. The polar terminal groups also controlled the phase behavior of hockey stick‐shaped Schiff bases **1** with chalcone moiety [9]. Polar‐end 3,5‐diaryloxazole LCs **2** not only displayed SmA and highly ordered CrE mesophases, but also possessed ionic conductivity [10, 11, 12, 13]. Terminal groups in terphenyl LCs **3** enhanced the birefringence and dielectric anisotropy, which is helpful for high blue light stability in augmented reality displays [14]. In (−)‐menthol‐based blue phase LCs **4,** the terminal groups not only influenced the mesomorphic properties, but also the polarizability anisotropy [15].

An attractive core unit for emissive calamitic LCs is fluorenone. Both aryl units as well as the carbonyl group allow further synthetic manipulation and thus tailoring of both mesomorphic and photophysical properties. The lateral dipole moment at the rigid core has been found beneficial for tilted smectic (SmC) phases in both achiral (**5**) [16], chiral (**6**) [17, 18, 19, 20, 21, 22, 23], ionic LCs (**7**) [24] (Scheme 1b), as well as lyotropic LCs [25]. In addition, fluorenones were reported to stabilize biaxial nematic phases [26, 27, 28, 29, 30] and twist‐bend nematic phases (**8**) [31]. Besides mesogenic fluorenones, 2,4,7‐trinitrofluorenone (TNF) has been successfully utilized to induce switching of columnar mesophases in mixtures with disk‐like compounds [32]. Moreover, fluorenone derivatives have been successfully applied for two‐color switching [33], multi‐stimuli‐responsive materials and LED devices [34, 35], solvatochromic materials [36, 37, 38], water sensing [34, 35, 36, 37], OLEDs [39], and light‐emitting LCDs [40, 41, 42].

With regard to the above‐discussed role of polarity on the LC self‐assembly as well as dye properties, surprisingly little work has been carried out on fluorenone derivatives. Previously, we reported that the attachment of highly polar N‐methyl‐imidodiacetate (MIDA) boronate on simple alkoxyphenyls (and ‐biphenyls) (**10** and **11**) resulted in stable SmA phases [43], while the corresponding boronic acids (**9**) or pinacol borolanes were non‐mesomorphic (Scheme 1c). The threefold increase of the molecular dipole moment (as compared to boronic acids) by the MIDA group also induced SmA mesophases in fluorenones **12** [44].

Thus, in the current work, we aimed at unsymmetrical decyloxyphenyl‐fluorenones **X‐FO‐Ar** with different polar terminal groups X = Br, NO_2_, NH_2_, and CN and compared them with the parent unsubstituted derivative **H‐FO‐Ar** (X  =  H) regarding mesomorphic and photophysical properties. Our results revealed that the polar terminal group has a large impact on the mesophase type and stability and on the photophysical properties like absorption and emission behavior and quantum yields in the solid state and solution.

## 2 Results and Discussion

### 2.1 Synthesis

The synthesis of the liquid‐crystalline fluorenone derivatives with various headgroups started from commercially available fluorenone **H‐FO‐H**, 2‐bromofluorenone **H‐FO‐Br,** and 2,7‐dibromofluorenone **Br‐FO‐Br**. Here, the mesogenic unit was introduced via Suzuki coupling (Scheme 2).

**SCHEME 2:**
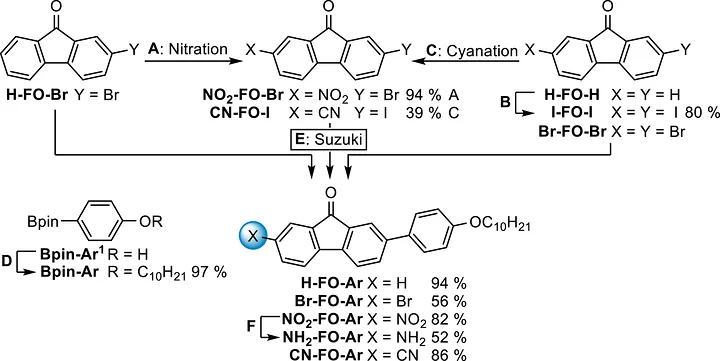
Synthesis of unsymmetrical fluorenone derivatives with various headgroups. (A) HNO_3_, H_2_SO_4_, H_2_O, 120°C, 3 h. (B) I_2_, H_5_IO_6_, H_2_SO_4_, AcOH, H_2_O, 85°C, 18 h. (C) NH_4_HCO_3_, Ag_2_CO_3_, Cu(NO_3_)_2_ (20 mol%), 2‐aminopyridine (20 mol%), DMF, O_2_‐atm, 150°C, 18 h. (D) BrC_10_H_21_, K_2_CO_3_, MeCN, reflux, 48 h, (E) **Bpin‐Ar**, Cs_2_CO_3_, Pd(PPh_3_)_4_, THF / H_2_O (10:1), 80°C, 18–72 h, and (F) NH_4_HCO_2_, Pd/C, THF, 60°C, 1 h.

For the nitro‐substituted derivative **NO_2_‐FO‐Ar,** 2‐bromofluorenone **H‐FO‐Br** was treated with nitric acid and sulfuric acid, which afforded nitro fluorenone **NO_2_‐FO‐Br** in 73% [45]. The nitrile‐substituted fluorenone **CN‐FO‐Ar** was prepared starting from fluorenone **H‐FO‐H**. For this, fluorenone **H‐FO‐H** was treated with iodine, periodic acid, and sulfuric acid in acetic acid and water, affording the diiodo fluorenone **I‐FO‐I** in 80% yield [46]. Following a procedure for the catalytic cyanation from Chang et al. with ammonium bicarbonate as nitrogen source, silver carbonate as oxidizing agent, copper(II) nitrate as catalyst, and 2‐aminopyridine as ligand in DMF under an oxygen atmosphere yielded the nitrile **CN‐FO‐I** in 39% [47].

For the introduction of the mesogenic unit via Suzuki coupling, a boronic acid ester was used. The pinacol borolane (Bpin) **Bpin‐Ar** was prepared by Williamson etherification of commercially available 4‐hydroxyphenyl pinacol borolane **Bpin‐Ar^1^** with decyl bromide, which yielded the desired product in 97 %.

Suzuki cross coupling of **H‐FO‐Br**, **Br‐FO‐Br**, **NO_2_‐FO‐Br**, and **CN‐FO‐I** with pinacol borolane **Bpin‐Ar**, cesium carbonate, and Pd(PPh_3_)_4_ in THF/H_2_O (10:1) yielded the desired substituted fluorenone derivatives **H‐FO‐Ar**, **Br‐FO‐Ar**, **NO_2_‐FO‐Ar**, and **CN‐FO‐Ar** in 56%–94% after purification by column chromatography. The decreased yield for the bromo‐substituted derivative **Br‐FO‐Ar** is due to double cross‐coupling of **Br‐FO‐Br** with two pinacol borolanes **Bpin‐Ar**, which could not be suppressed by adjusting the reaction conditions. Subsequent hydrogenation of the nitro‐substituted derivative **NO_2_‐FO‐Br** with ammonium formate and palladium on carbon afforded the amino‐substituted fluorenone **NH_2_‐FO‐Ar** in 52% yield [48].

### 2.2 Mesomorphic Properties

The influence of the headgroup on the mesomorphic properties of fluorenone derivatives **X‐FO‐Ar** was examined using polarized optical microscopy (POM), differential scanning calorimetry (DSC) and small‐ and wide‐angle x‐ray diffraction (SAXS and WAXS). All derivatives except for the amine **NH_2_‐FO‐Ar** showed mesomorphic behavior. **Br‐FO‐Ar**, **NO_2_‐FO‐Ar**, and **CN‐FO‐Ar** each displayed one enantiotropic mesophase with clearing temperatures around 200°C (Figure 1b–d). **CN‐FO‐Ar** showed a mesophase between 203°C and 140°C, leading to a phase width of 63 K (Figure 1d). For **Br‐FO‐Ar** and **NO_2_‐FO‐Ar,** supercooling occurred, which resulted in even larger mesophase widths of 145 K and 128 K, respectively (Figure 1b,c). In contrast, the derivative without a headgroup **H‐FO‐Ar** shows two mesophases, of which the high‐temperature mesophase between 116°C and 79°C is enantiotropic and the lower‐temperature mesophase from 79°C to 38°C is monotropic (Figure 1a). For further details, see Chapter S3.2.

**FIGURE 1:**
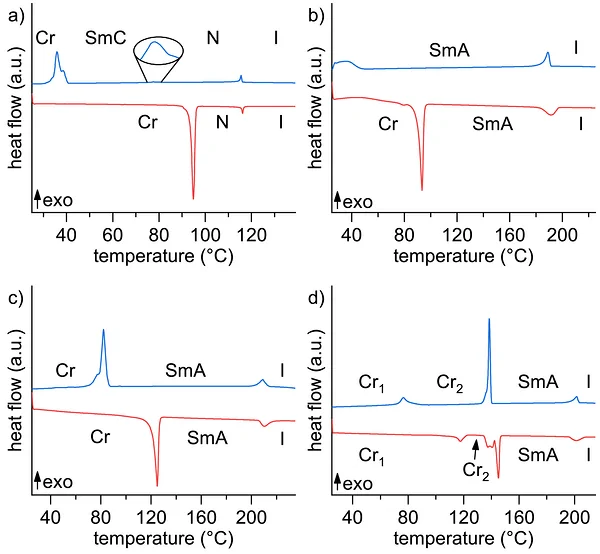
DSC thermograms of (a) **H‐FO‐Ar**, (b) **Br‐FO‐Ar**, (c) **NO_2_‐FO‐Ar**, and (d) **CN‐FO‐Ar**. Second cycle, red: heating, blue: cooling, heating rate: 10 K/min.

First indications on the type of the respective mesophases were obtained via POM. The derivative without headgroup **H‐FO‐Ar** did not show any textures for the high‐temperature phase due to homeotropic alignment. By applying pressure, the alignment could be disturbed, and bright colorful flashes were visible, which are characteristic of a N phase. Upon further cooling, Schlieren textures appeared (Figure 2a). Because only fourfold defects were visible, a SmC phase could be assigned. **Br‐FO‐Ar**, **NO_2_‐FO‐Ar**, and **CN‐FO‐Ar** showed focal conic and fan‐shaped textures as well as Maltese crosses, which suggests a SmA phase for these three derivatives (Figure 2b–d).

**FIGURE 2:**
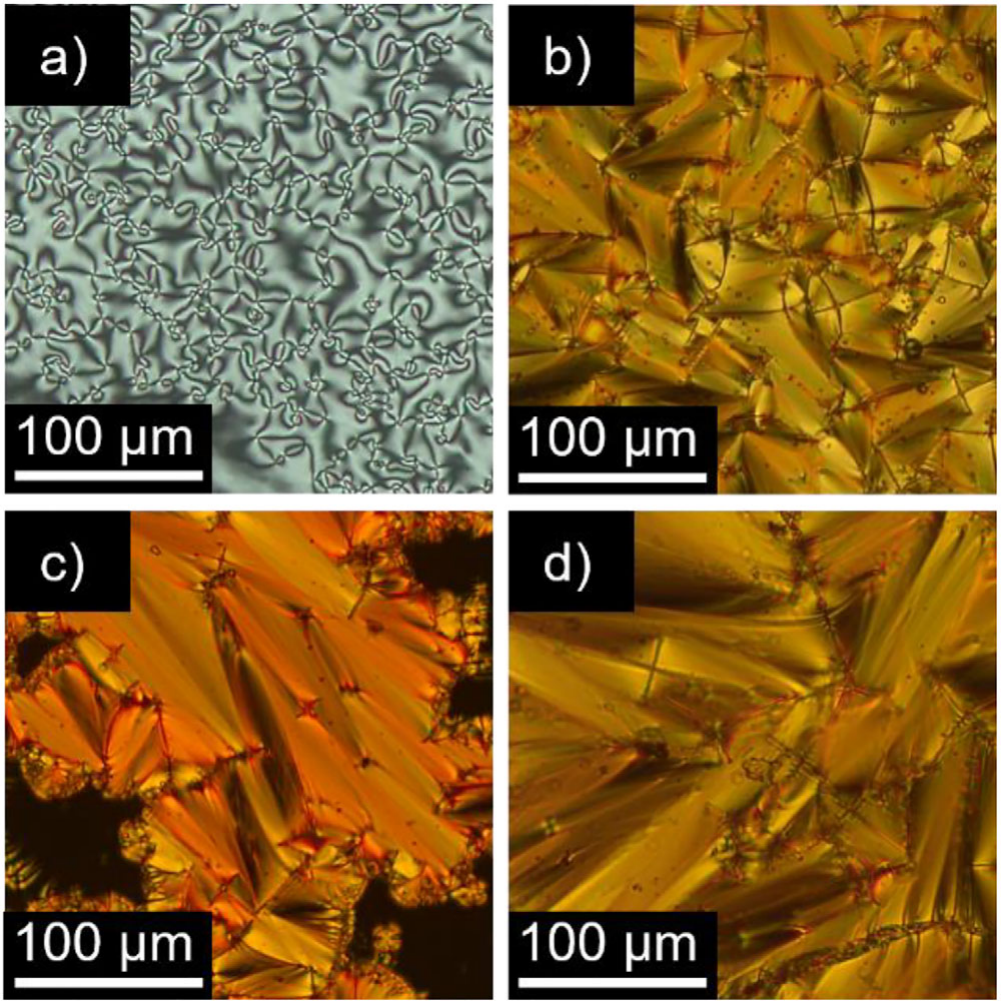
POM microphotography obtained upon cooling (5 K∙min^−1^) of (a) **H‐FO‐Ar** at 72°C, (b) **Br‐FO‐Ar** at 205°C, (c) **NO_2_‐FO‐Ar** at 215°C, and (d) **CN‐FO‐Ar** at 195°C.

To confirm the previously assigned mesophases, **H‐FO‐Ar**, **Br‐FO‐Ar**, **NO_2_‐FO‐Ar**, and **CN‐FO‐Ar** were analyzed with SAXS and WAXS measurements. The WAXS diffractogram of **H‐FO‐Ar** at a higher temperature shows a diffuse reflex in the small‐angle area, which is also visible in the SAXS diffractogram, and a diffuse halo in the wide‐angle area (Figure 3a,b). This diffraction pattern is characteristic of the nematic phase and confirms the previous assignment. By cooling into the next mesophase, a sharp (001) reflex occurs in the SAXS and WAXS, as well as a (002) reflex in the latter case, which confirms a smectic phase (Figure 3c,d). To confirm that the smectic phase is a SmC phase, temperature‐dependent SAXS measurements were performed. From the (001) reflex of each measurement, the corresponding layer spacing was calculated and plotted against the temperature (Figure 3e). The layer spacing increases exponentially with increasing temperature, which is characteristic of the SmC phase [49]. Also, the 2D diffraction pattern shows that the halo is tilted more than 90° with respect to the small‐angle reflex by a tilt angle *θ* = 9°.

**FIGURE 3:**
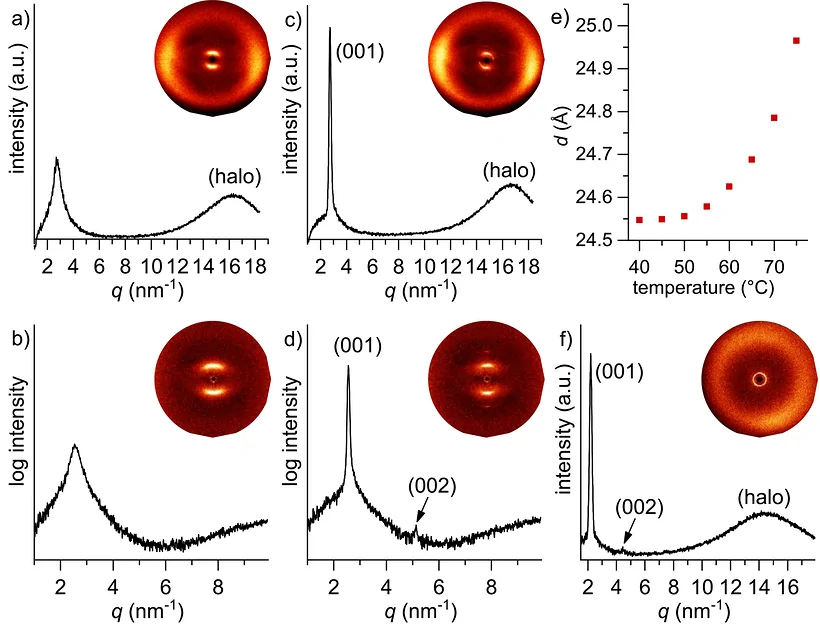
Diffractograms with diffraction patterns of **H‐FO‐Ar** (a) WAXS and (b) SAXS at 90°C, (c) WAXS and (d) SAXS at 50°C. (e) Temperature‐dependent layer spacing of the SmC phase of **H‐FO‐Ar**. (f) WAXS diffractogram with diffraction pattern of **Br‐FO‐Ar** at 140°C.

For **Br‐FO‐Ar**, **NO_2_‐FO‐Ar**, and **CN‐FO‐Ar**, the WAXS and SAXS diffractograms were similar; thus, the WAXS of **Br‐FO‐Ar** is shown exemplary (Figure 3f). All WAXS diffractograms showed a broad halo in the wide‐angle area and a sharp (001) reflex in the small‐angle area, which was also present in the SAXS diffractograms (Figures S9–S11). The WAXS and SAXS of **Br‐FO‐Ar** showed an additional (002) reflex (Figures 3f and S9). This observation confirms the previously assigned SmA phase for all three derivatives.

Detailed XRD data are shown in Table 1. For **H‐FO‐Ar**, the calculated average distance of the molecules along the long axis in the N phase (*d*_exp_ = 24.54) as well as the layer spacing of the SmC phase (*d*_exp_ = 24.54) are in good agreement with the molecule length (*L* = 24.0 Å) (molecule lengths determined with Chem3D). Since the mesogens in a SmC phase are tilted, the layer thickness is typically lower than the molecule length; however, this effect is marginal due to the small tilt angle of *θ* = 9°.

**TABLE 1:** Detailed x‐ray diffraction data (reflexes determined from SAXS except halo).

Compound	Mesophase	*q*/nm^−1^	(*hkl*)	*d*_exp_/Å	*d*_calc_/Å
**H‐FO‐Ar**	N at 90°C	2.56	diffuse reflex	24.54	—
		16.26	(halo)	3.86	—
	SmC at 50°C	2.56	(001)	24.54	—
		5.12	(002)	12.27	12.27
		16.61	(halo)	3.78	—
**Br‐FO‐Ar**	SmA at 140°C	2.24	(001)	28.05	—
		4.48	(002)	14.02	14.02
		14.48	(halo)	4.34	—
**NO_2_‐FO‐Ar**	SmA at 169°C	1.83	(001)	34.33	—
		15.05	(halo)	4.17	—
**CN‐FO‐Ar**	SmA at 169°C	1.60	(001)	39.27	—
		13.94	(halo)	4.51	—

For **Br‐FO‐Ar**, the layer spacing of the SmA phase (*d*_exp_ = 28.05) is slightly larger than the molecule length (*L* = 25.5 Å) and indicates a monolayer structure (Figure 4, left). For the derivatives **NO_2_‐FO‐Ar** (*d*_exp_ = 34.33, *L* = 26.2 Å) and **CN‐FO‐Ar** (*d*_exp_ = 39.27, *L* = 26.1 Å) the layer spacing is significantly larger than the molecule length but not twice as big (*L* < *d* < 2*L*). This suggests a bilayer structure with fully interdigitated alkyl chains (Figure 4, right).

**FIGURE 4:**
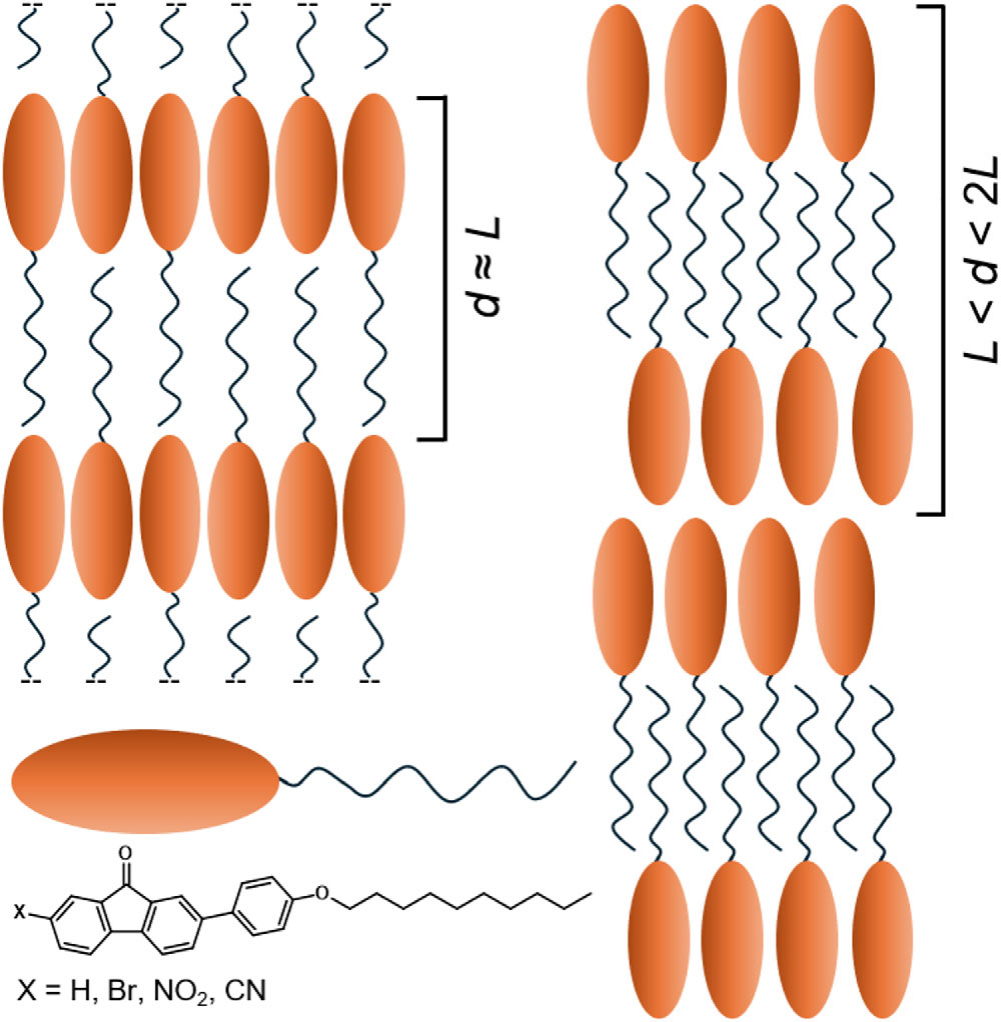
Proposed molecular arrangement in the mesophase for left: a monolayer, right: a bilayer with interdigitation of the alkyl chains.

Figure 5 gives an overview of the mesophases of all derivatives. **H‐FO‐Ar** is the only derivative that displays two mesophases, where the N and SmC phases are unique to this derivative in this series. Also, the clearing temperature (116°C) of this derivative is significantly lower than for the other derivatives. **Br‐FO‐Ar**, **NO_2_‐FO‐Ar**, and **CN‐FO‐Ar** all showed a SmA phase with clearing temperatures around 200°C. From these three derivatives, **Br‐FO‐Ar** shows the lowest melting point, and thus the broadest phase width of 145 K. Switching to an amino headgroup for **NH_2_‐FO‐Ar** led to the disappearance of mesomorphic behavior. Presumably, this is due to intermolecular hydrogen bonding between the NH_2_ group and the carbonyls of two neighboring fluorenone molecules. This leads to a strong interaction between the molecules, resulting in high order and disturbing π–π stacking, which suppresses liquid crystalline properties. The observed high melting/decomposition temperature might be taken as another hint for the stable structure in the crystalline state.

**FIGURE 5:**
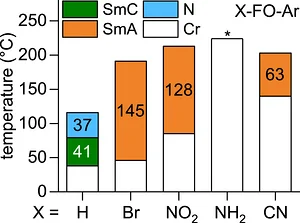
Phase diagram of compound **X‐FO‐Ar**. Transition temperatures determined from second cooling cycle (heating rate 10 K/min). *Determined from first heating cycle due to decomposition.

### 2.3 Photophysical Properties

The influence of the headgroup on the photophysical properties of fluorenone derivatives **X‐FO‐Ar** was examined via absorption and emission measurements, absolute quantum yields (AQY), and fluorescence lifetime measurements in solid state and solution (different solvents). Absorption spectra were measured in CH_2_Cl_2_ (DCM) with concentrations of *c* = 0.01–0.02 mM.

Exemplary absorption spectra of **H‐FO‐Ar** and **CN‐FO‐Ar** are shown (Figure 6a,b; for details see Chapter S4.1). All derivatives show one large absorption maximum around 266–292 nm with up to two additional peaks or shoulders, which were attributed to *π*–π* and n–π* transitions [34, 35, 39]. Further, they show one smaller broad band located in the visible area around 432–492 nm, which was assigned to the intramolecular charge transfer (ICT) [34, 35, 39]. For **H‐FO‐Ar**, **Br‐FO‐Ar**, **NO_2_‐FO‐Ar**, and **CN‐FO‐Ar** the broad band is located between 432–446 nm, which results in a yellow to orange color of the compounds, whereas for **NH_2_‐FO‐Ar** this band is shifted bathochromically to 492 nm, which results in a purple color. Further, the absorption maxima of **H‐FO‐Ar**, **Br‐FO‐Ar**, **NH_2_‐FO‐Ar,** and **CN‐FO‐Ar** are located between 282 and 292 nm, while for **NO_2_‐FO‐Ar** the maximum is located at a lower wavelength (266 nm). The absorption maxima with their respective extinction coefficients for all **X‐FO‐Ar** derivatives (X = H, Br, NO_2_, NH_2_, and CN) are summarized in Table 2.

**FIGURE 6:**
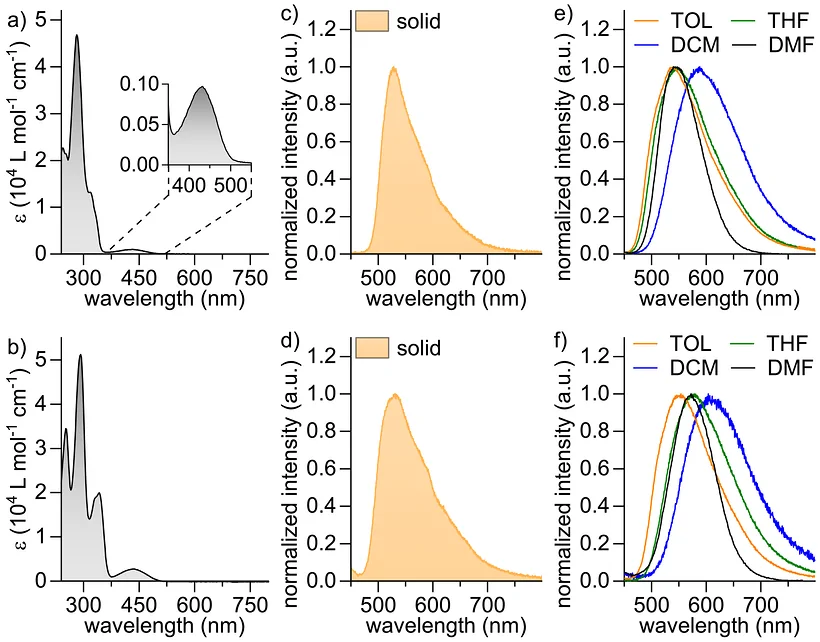
Absorption spectrum of (a) **H‐FO‐Ar** and (b) **CN‐FO‐Ar** in DCM. Emission spectra of (c) **H‐FO‐Ar** in the solid state, (d) **CN‐FO‐Ar** in the solid state, and (e) **H‐FO‐Ar** in toluene (TOL), THF. DCM and DMF, (f) **CN‐FO‐Ar** in TOL, THF. DCM and DMF. Details in Chapters S4.1 and S4.2.

**TABLE 2:** Absorption maxima *λ*_abs_ in DCM with respective extinction coefficients.

Compound	*λ*_abs,1_/nm	*λ*_abs,2_/nm	*λ*_abs,3_/nm	*λ*_abs,4_/nm
	(ε_1_/mol^−1^cm^−1^)	(ε_2_/mol^−1^cm^−1^)	(ε_3_/mol^−1^cm^−1^)	(ε_4_/mol^−1^cm^−1^)
**H‐FO‐Ar**	244 (23,000)	282 (47,000)	318^a^ (13,000)	432 (1000)
**Br‐FO‐Ar**	252 (15,000)	286 (36,000)	322 (11,000)	446 (1000)
**NO_2_‐FO‐Ar**	266 (28,000)	292^a^ (23,000)	360 (15,000)	432 (4000)
**NH_2_‐FO‐Ar**	—	290 (56,000)	340 (19,000)	492 (1000)
**CN‐FO‐Ar**	252 (34,000)	292 (51,000)	342 (20,000)	434 (3000)

^a^ Shoulder.

The emission properties were examined in the solid state and in solution (toluene, THF, DCM, and DMF) and are shown exemplary for **H‐FO‐Ar** (Figure 6c,e) and **CN‐FO‐Ar** (Figure 6d,f). All derivatives except **NH_2_‐FO‐Ar** are brightly emissive in the solid state and in solution (details see Chapter S4.2). These results show that **NH_2_‐FO‐Ar** differs from the other compounds in both its absorption and emission properties, where the absorption is shifted to longer wavelengths, and no emission is observed. These results further demonstrate the unique electronic effect of the amino group on the fluorenone as compared to the other substituents. This is in agreement with the observed absence of mesomorphism. The emission maxima and full width half maximum (FWHM) values are summarized in Table 3. In the solid state, **H‐FO‐Ar** and **CN‐FO‐Ar** exhibit a yellow emission (*λ*_em_ = 528 and 530 nm), whereas **Br‐FO‐Ar** and **NO_2_‐FO‐Ar** show an orange emission (*λ*_em_ = 594 and 571 nm) (Figure 6c,d).

**TABLE 3:** Detailed emission properties. Emission maxima *λ*_em_ with respective FWHM, AQY *Φ*, fluorescence lifetimes *τ*, χ^2^ values and rate constants for radiative and nonradiative deactivation *k*_r_ and *k*_nr_ in solution and solid state.

	solvent	*E*_T_(30)^b^	H‐FO‐Ar	Br‐FO‐Ar	NO_2_‐FO‐Ar	CN‐FO‐Ar
*λ*_em_/nm	Solid	—	528 [81] (2730)	594 [103] (2771)	571 [96] (2837)	530 [102] (3453)
FWHM/[nm] (cm^−1^)	TOL	33.9	540 [121] (4002)	556 [124] (3862)	551 [119] (3767)	554 [124] (3929)
	THF	37.4	544 [123] (3966)	575 [130] (3871)	583 [133] (3731)	578 [132] (3826)
	DCM	40.7	588 [141] (3928)	597 [147] (3851)	618 [141] (3630)	604 [143] (3752)
	DMF	43.2	540 [88] (2896)	563 [90] (2797)	591 [92] (2677)	572 [94] (2852)
	Solid^h^	—	—	—	—	591 [87] (2336)
Φ/%	Solid^a^	—	35 [6.1] (0.99)	16 [3.6] (1.11)	2 [7.4] (1.09)	10 [5.9] (1.38)
[τ/ns]	TOL	33.9	14 [19.2] (1.17)	7 [2.8] (1.15)	6 [7.7] (1.09)	20 [10.7] (1.00)
(χ^2^)	THF	37.4	15	6	27	17
	DCM	40.7	2	1	4	6
	Solid^h^	—	—	—	—	13 [7.8] (1.06)
*k*_r_│k_nr_ /	TOL	33.9	5.73│10.64	4.44│23.33	0.27│13.20	1.68│15.15
10^7^ s^−1^	Solid	—	0.73│4.47	2.50│33.15	0.78│12.24	1.86│7.45
	Solid^h^	—	—	—	—	1.67│11.14

^a^ Emission decays were fitted with two components for **Br‐FO‐Ar**, **NO_2_‐FO‐Ar**, **CN‐FO‐Ar**. Average lifetimes *τ*_av_ were calculated as *τ*_av_ = (a_1_τ_1_^2^+a_2_τ_2_^2^)/(a_1_τ_1_+a_2_τ_2_).

^b^ *E*_T_(30) values in kcal mol^−1^ were taken from the literature [52].

To examine solvatochromic emission behavior, solvents with different polarities were used. For this, the polarity was increased stepwise from toluene (TOL) as the least polar solvent via THF and DCM to DMF as the most polar solvent (Figure 6e,f and Table 3). In the least polar solvent TOL, the emission peaks of **Br‐FO‐Ar** and **NO_2_‐FO‐Ar** are located at lower wavelengths (*λ*_em_ = 556 and 551 nm) compared to the solid state, whereas for **H‐FO‐Ar** and **CN‐FO‐Ar** the peaks are shifted to higher wavelengths (*λ*_em_ = 540 and 554 nm) as in the solid state. The increased polarity of THF had a marginal effect on the emission of **H‐FO‐Ar** and **Br‐FO‐Ar**, whereas the emission maxima of the more polar derivatives **NO_2_‐FO‐Ar** and **CN‐FO‐Ar** are bathochromically shifted (*λ*_em_ = 583 and 578 nm). Further increase of the polarity to DCM has a significant influence on the emission band of all derivatives. The emission maxima are bathochromically shifted (*λ*_em_ = 588–618 nm), and the bands are broadened (FWHM = 3630–3928 cm^−1^). When using the most polar solvent, the emission wavelength does not increase further. Instead, the emission maxima for all derivatives occur at lower wavelengths (*λ*_em_ = 540–591 nm), similar to those in THF. Similar behavior was already reported in the literature [38]. This result cannot be explained by the polarity of the solvent, because the emission is influenced by multiple factors [50]. It is presumably due to the large dipole in DMF, which can interact with the lateral dipole of the fluorenone core and the polar head groups via dipole–dipole interactions and thus counteract the bathochromic shift. The emission is also influenced by hydrogen bonding that is present in DCM (weak hydrogen‐bond donor) but not in DMF (only a hydrogen‐bond acceptor), which leads to a further bathochromic shift in DCM than in DMF [51]. Furthermore, less broadening of the emission band is observed, which is possibly due to the solvation environment of the compound in DMF [50]. Stokes shifts of **X‐FO‐Ar** derivatives (X = H, Br, NO_2_, and CN) in DCM were in the range of Δ$\bar{\nu }$ = 5670–6974 cm^−1^ (Table S4).

The nitrile derivative **CN‐FO‐Ar** also displayed a unique property. Upon heating, the visible color and the emission color changed from yellow to orange in the solid state at 122°C (Figure 7b). This color change was not reversible by cooling to room temperature, which would be the case for classical thermochromism.

**FIGURE 7:**
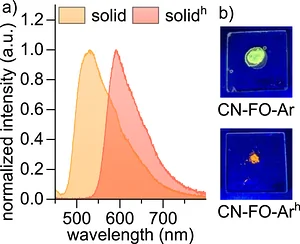
(a) Emission spectra of **CN‐FO‐Ar** in the solid state and in the solid state after heating (solid^h^), (b) **CN‐FO‐Ar** and **CN‐FO‐Ar^h^** in the solid state under UV light.

Thus, measurements were performed at ambient temperature after heating above 122°C and subsequent cooling. The heated form of **CN‐FO‐Ar** is labeled as solid^h^ or **CN‐FO‐Ar^h^** in the following. Figure 7a shows the emission bands of **CN‐FO‐Ar** and **CN‐FO‐Ar^h^**. After heating, a bathochromic shift of the emission maximum from *λ*_em_ = 530 nm to *λ*_em_ = 591 nm occurs, accompanied by sharpening of the peak (from FWHM = 3453 cm^−1^ to FWHM = 2336 cm^−1^).

The nitrile derivative **CN‐FO‐Ar** was further investigated regarding the change of emission color from yellow to orange at 122°C, which is not reversible by cooling to ambient temperature. To exclude the occurrence of a chemical reaction, ^1^H‐NMR and mass spectra of **CN‐FO‐Ar** were measured before and after heating (**CN‐FO‐Ar^h^**), which were identical (Figures S64–S68). Interestingly, the color change is not completely irreversible. By dissolving **CN‐FO‐Ar^h^** and subsequent evaporation of the solvent, the initial yellow form is recovered. This suggests that the change in emission color is due to a change in the molecular arrangement. To gain more insight into the structure, single‐crystal x‐ray diffraction of **CN‐FO‐Ar** was performed. The obtained crystal structure is shown in Figure 8 (for details see Chapter S5). The molecules are arranged in a bilayer where the nitriles overlap in an antiparallel fashion (Figure 8b) and the ketones point in the same direction on each side, with both sides antiparallel to each other (Figure 8a). The 3D structure is assembled by weak hydrogen bonds between the carbonyls and the aromatic C─H in 4‐ and 5‐positions. After heating above 122°C, the single crystal was no longer intact, which shows a change in the molecular arrangement. Unfortunately, it was not possible to obtain a single crystal of **CN‐FO‐Ar^h^**.

**FIGURE 8:**
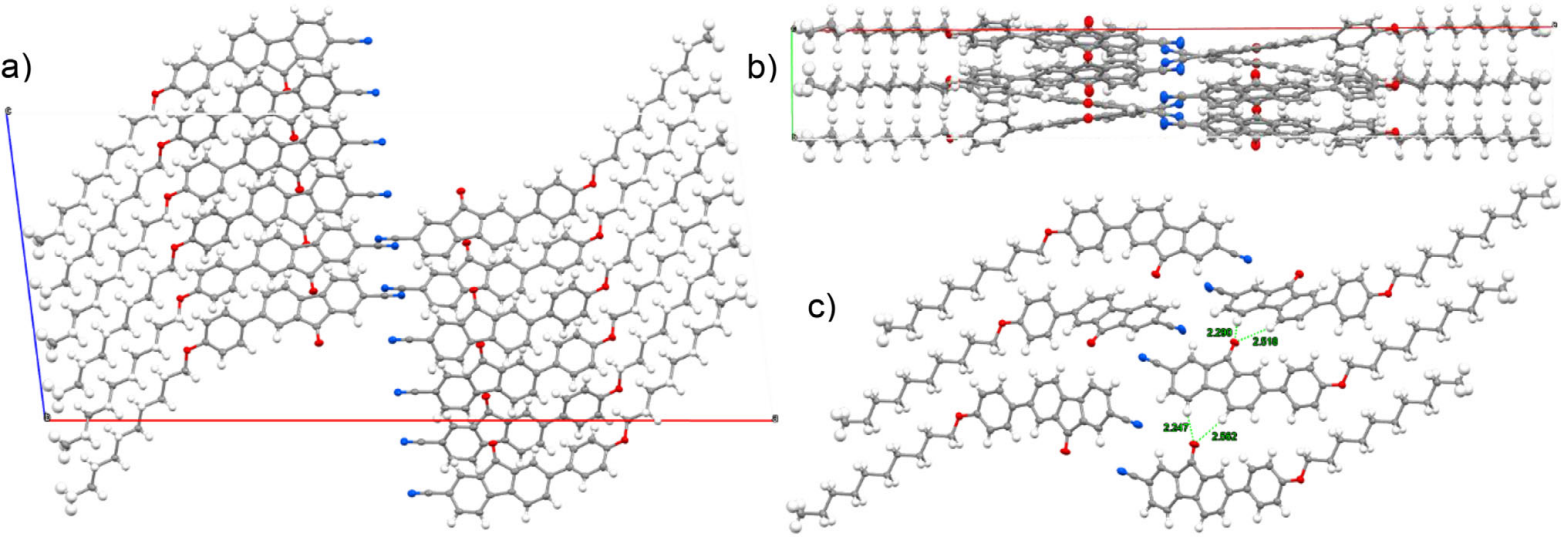
Crystal structure of **CN‐FO‐Ar** obtained from single‐crystal x‐ray diffraction. (a) Top view (along *b*‐axis), (b) side view (along *c*‐axis), (c) selected molecules to display intermolecular hydrogen bonds. Displacement ellipsoids drawn at the 50% probability level. Color code: O red, N blue, C gray, and H white.

A similar behavior was already reported as solid‐state luminescence switching (SLS) for fluorenone derivatives (e.g., 2,7‐bis(4‐methoxyphenyl)‐9*H*‐fluoren‐9‐one) by Yuan et al. [33]. In this case, two forms of the same compound with different colors were observed, which were stable at room temperature and could be converted into one another by heating. The color change was caused by a change in the crystal structure, where one structure was hydrogen‐bond‐directed, and the other was *π*–*π* stacking‐directed [33].

Like the results for the unheated form **CN‐FO‐Ar**, a hydrogen‐bond‐directed packing was observed. Upon heating, this packing could change to a π–π stacking‐directed packing. The orange color is also present in the mesophase in which π–π interactions are very common.

Further, the transition from yellow to orange emission was studied in a temperature‐dependent manner. For this, **CN‐FO‐Ar** was heated, and an emission spectrum was measured in 10 K steps. Figure 9 shows the emission profile upon heating. The emission intensity is slightly decreasing until the yellow‐to‐orange transition temperature is reached, where the intensity drastically increases. Upon transition to the mesophase, the intensity drops strongly. The emission wavelength remains unchanged until 100°C and drastically changes to the orange area after passing the transition temperature.

**FIGURE 9:**
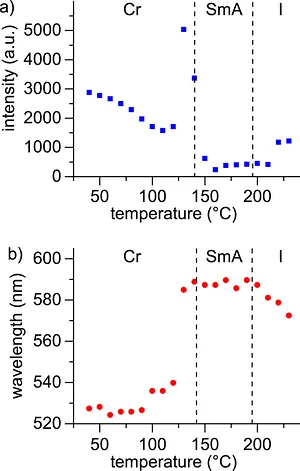
**CN‐FO‐Ar** (a) intensities and (b) wavelengths of emission maxima against the temperature with indicated phase transition temperatures.

Further, AQY of **X‐FO‐Ar** derivatives (X = H, Br, NO_2_, CN) was measured in the solid state and in solution. The AQY values are summarized in Table 3. For **H‐FO‐Ar** and **Br‐FO‐Ar,** aggregation‐induced emission (AIE) is observed with AQY values in the solid state (*Φ* = 35% and 16%) more than twice as high as in TOL (*Φ* = 14% and 7%). In contrast, **NO_2_‐FO‐Ar** and **CN‐FO‐Ar** showed aggregation‐caused quenching (ACQ), where the emission of **NO_2_‐FO‐Ar** (*Φ* = 2%) almost vanishes, and the AQY value of **CN‐FO‐Ar** is halved by switching from TOL (*Φ* = 20%) to the solid state (*Φ* = 10%). Interestingly, the AQY increases for **CN‐FO‐Ar** in the solid state after heating (*Φ* = 13%). The lowest AQY for most derivatives is found in DCM (*Φ* = 1–6%), where the emission is almost completely quenched. The overall highest AQY was observed for **H‐FO‐Ar** in the solid state (*Φ* = 35%). In THF, **NO_2_‐FO‐Ar** showed the highest AQY (*Φ* = 27%), whereas in TOL and DCM **CN‐FO‐Ar** displayed the highest AQY in this series. These results show that the headgroup has a major influence on the AQY; for example, in the solid state, derivatives with polar headgroups show ACQ, whereas the ones with less polar headgroups show AIE. Also, the AQY can be increased by the choice of the solvent (also none) for every derivative.

Furthermore, emission decay measurements were performed in TOL and in the solid state. Lifetime values were obtained by monoexponential fit of the decay profile except for **Br‐FO‐Ar**, **NO_2_‐FO‐Ar**, and **CN‐FO‐Ar** in the solid state, where a biexponential fit was necessary and therefore given as intensity‐averaged values. The lifetime values range from 2.8 to 19.2 ns and correspond to fluorescence processes. For most derivatives, the lifetime in the solid state is longer than in TOL, which is most pronounced for **H‐FO‐Ar**.

## 3 Conclusion

A series of novel unsymmetrical fluorenone derivatives bearing different headgroups (H, Br, NO_2_, NH_2_, and CN) was synthesized in overall yields of 26%–92% over two to four steps. POM, DSC, and XRD analysis revealed mesomorphic behavior for all compounds except for the amino‐substituted derivative. The Br‐, NO_2_‐, and CN‐substituted derivatives exhibited SmA phases with phase widths of up to 145 K, whereas the H‐substituted derivative displayed N and SmC phases.

The photophysical properties were investigated via absorption and emission measurements, AQY and lifetime measurements. All derivatives showed an absorption maximum around 266–292 nm with up to two additional bands or shoulders and one broad band with lower intensity located in the visible area around 432–492 nm, which is responsible for the yellow, orange, or purple color of the compounds. Except for the amino‐substituted derivative, all compounds exhibited bright yellow to orange fluorescence in the solid state (*λ*_em_ = 528–594 nm) and in solution (*λ*_em_ = 540–618 nm). Solvatochromic emission behavior was observed for all fluorescent derivatives with increasing bathochromic shift with increasing solvent polarity (toluene, THF, and CH_2_Cl_2_). Quantum yields of up to *Φ* = 35% were strongly dependent on the headgroup and the solvent (also no solvent). The derivatives with bromide and without head group exhibited AIE, whereas the more polar nitro‐ or nitrile‐substituted derivatives exhibited ACQ. Emission lifetimes were recorded in the solid state and toluene with values between 2.8 and 19.2 ns, which correspond to fluorescence processes.

The nitrile derivative exhibited a unique property where the visible color and emission color changed from yellow (*λ*_em_ = 530 nm) to orange (*λ*_em_ = 591 nm) in the solid state upon heating above 122°C. This shift was not reversible by cooling to room temperature as expected for typical thermochromism. However, by dissolving the heated form (orange) and subsequent evaporation, the initial form (yellow) is obtained. The color change was examined by temperature‐dependent emission measurements, and additionally, a crystal structure was obtained from single‐crystal x‐ray diffraction for further insight into the molecular arrangement. These results demonstrate that the mesomorphic and photophysical properties of the examined fluorenone derivatives are strongly dependent on the type of headgroup. Furthermore, the fluorescence properties can be significantly influenced by the solvent (also no solvent) or temperature.

## Author Contributions

F.F. synthesized and characterized the fluorenone derivatives, performed POM, DSC, and XRD measurements, and wrote the first draft of the manuscript. F.F. and F.M. performed the photophysical experiments. J.O. performed single‐crystal x‐ray diffraction and solved the crystal structure. Y.M. supervised and performed the photophysical experiments. F.F., F.M., and A.Z. checked the data and helped with the manuscript preparation. F.F., Y.L., and S.L. wrote the manuscript; S.L. coordinated the research.

## Conflicts of Interest

The authors declare no conflicts of interest.

## Supporting information




**Supporting File 1**: chem71542‐sup‐0001‐SuppMat.pdf

## Data Availability

All data supporting the findings of this publication are included in the Supporting Information file accompanying this article. Supporting Information: Experimental procedures, NMR spectra; Details of x‐ray crystallography, DSC, and POM; photophysical data and supplementary experiments.
