# Supplementary material for: Tailoring Liquid Crystalline Self‐Assembly and Photophysical Properties of Unsymmetrical Fluorenones via Polar Terminal Groups
Source: Chemistry. 2026 Aug 13;32(41):e71542. doi: 10.1002/chem.71542 (PMC13730022; doi:10.1002/chem.71542)
Supplement: Supplementary file 1 [file CHEM-32-e71542-s001.pdf]

Electronic Supporting Information (ESI)

Tailoring Liquid Crystalline Self-Assembly and Photophysical Properties of Unsymmetrical Fluorenones via Polar Terminal Groups

Falk Feucht*[,1], Franziska Müller[1], Johanna Osterbrink[1], Anna Zens[1], Yann Molard*[,2], Sabine Laschat*[,1]

[1]Institut für Organische Chemie, Universität Stuttgart, Pfaffenwaldring 55, D-70569, Stuttgart, Germany, [2]Univ Rennes, CNRS, ISCR─UMR 6226, ScanMAT─UAR 2025, F-35000 Rennes, France

Content

1. General Methods S1
  1.1. Synthesis S1
  1.2. Mesomorphic properties S2
  1.3. Photophysical properties S2
2. Synthesis S4
3. Mesomorphic properties S12
  3.1. POM results S12
  3.2. DSC results S13
  3.3. XRD results S15
4. Photophysical properties S18
  4.1. Absorption S18
  4.2. Emission in solid state and solution S20
  4.3. Fluorescence lifetime S29
  4.4. Temperature dependent emission S34
5. Crystal structure S35
6. NMR spectra S49
7. Mass spectra S68
8. References S70

1. General Methods

1.1. Synthesis

Methods and Chemicals

Commercially available chemicals were used without further purification. Air and moisture sensitive reagents were handled by using Schlenk technique under N_2 atmosphere in dry solvents. THF was dried by distillation over potassium. Petroleum ether, EtOAc and CH_2Cl_2 were distilled before use.

Chromatography

Purification via column chromatography was performed on silica gel (type F60, grain diameter 40-63 μm) from Silicycle. The columns were packed with the specified solvent mixtures and the crude products were applied as a solution in CH_2Cl_2 or on Celite®. For thin-layer chromatography Alugram® Xtra SIL G/UV254 prefabricated plates from Machery-Nagel were used. The compound stains were detected under UV light (λ = 254, 365 nm).

NMR spectroscopy

^{1}H NMR spectra were measured at 400 MHz or 700 MHz, ^{13}C NMR spectra at 101 MHz or 176 MHz on AVANCE III HD 400 NanoBay and AVANCE III HD 700 spectrometers from Bruker. The chemical shifts δ are reported in parts per million (ppm) and referenced to the signal of the deuterated solvent $CDCl_3$ (δ = 7.26 ppm).[1] Signal multiplicities were given with the respective coupling constants *J* in Hertz (Hz) and abbreviated as follows: singlet (s), doublet (d), triplet (t), multiplet (m). The signals were assigned using COSY, HSQC and HMBC spectra.

Mass spectrometry

Mass spectrometry (MS) and high-resolution mass spectrometry (HRMS) were performed using electron spray ionization (ESI) or atmospheric-pressure chemical ionization (APCI) on a Thermo Fisher Scientific Exactive Plus Orbitrap mass spectrometer or using electron impact ionization (EI) or chemical ionization with methane (CI) on a Thermo Fisher Scientific Exactive GC Orbitrap mass spectrometer.

Infrared spectroscopy

IR spectra were recorded using a Bruker Alpha FT-IR spectrometer. The absorption bands are given in wavenumbers $\tilde{\nu}$ (as whole numbers) and their intensities are described as follows: very strong (vs), strong (s), medium (m) weak (w).

1.2. Mesomorphic properties

Polarized optical microscopy (POM)

The mesomorphic properties were examined with a polarized optical microscope BX50 from Olympus with a heating chamber HCS302, temperature control unit mK2000B and cooling pump LN2-P4C from Instec. The microphotographs of the textures were taken with an Axiocam 105 color camera module and the software ZEN 3.10 from Zeiss. The samples were prepared on regular glass slides.

Differential scanning calorimetry (DSC)

DSC-thermograms were recorded on a DSC3 calorimeter from Mettler-Toledo. For the measurements Standard 40 µL aluminum crucibles were used and the evaluation was carried out with the STARe 16.40 software. The temperatures of the phase transitions are given as onset values of the corresponding peaks and the enthalpies ΔH were calculated from the integrals.

Small- and wide-angle X-ray diffraction (SAXS and WAXS)

The temperature dependent ($\Delta T = \pm 1$ K) measurement of small- and wide-angle X-ray diffraction patterns was performed on a Bruker AXS Nanostar C with a Siemens ceramic tube generator (CuK_{α} radiation, $\lambda = 1.5405$ Å, 1500 W) and a Bruker HI-STAR detector. The diffraction pattern of silver behenate at 25 °C was used for calibration. All samples were prepared in sealed cappilaries by Hildenberg GmbH (external diameter of 0.7 mm, wall thicknes 0.01 mm). The evaluation of the measurements was carried out with SAXSoffline from Bruker, Datasqueeze from Datasqueeze Software and OriginPro 2018 from OriginLab.

1.3. Photophysical properties

UV/Vis spectroscopy and fluorescence spectroscopy

Absorption and emission spectra and excitation/emission maps in solution and solid state were recorded on a Horiba Jobin Yvon Duetta spectrophotometer at concentrations of $c = 0.01 – 0.02$ mM. Emission measurements in DMF were carried out on a LS 55 fluorescence spectrometer from Perkin Elmer.

Quantum yields

The absolute quantum yields were measured with a C9920–03 Hamamatsu system.

Emission lifetimes

Lifetime measurements and TRPL mapping at 296 K were realized using a picosecond laser diode (Jobin Yvon deltadiode, 375 nm) and a Hamamatsu C10910-25 streak camera mounted with a slow single sweep unit. Signals were integrated on the whole emission decay. Fits were

obtained using origin software and the goodness of fit judged by the reduced $\chi 2$ value and residual plot shape.

Emission in the mesophase

Measurements of the emission in the mesophase were carried out on a Nikon 80i POM equipped with a Linkam LTS420E heating table, a Nikon Intensilight source and an ocean optics QE6500 photodetector.

Single crystal X-ray diffraction

Single crystal X-ray diffraction was performed on a XtaLAB Synergy R, DW system, HyPix-Arc 100 diffractometer. The crystal was kept at 100.00(10) K during data collection. The obtained data was processed using the standard Rigaku software package (CrysalisPro).[2] Using Olex2[3], the structure was solved with the SHELXT[4] structure solution program using Intrinsic Phasing and refined with the SHELXL[5] refinement package using Least Squares minimization (Version 2019/3).

All non-hydrogen atoms received anisotropic displacement parameters. Hydrogen atoms were generally placed at calculated positions and refined with a riding model. The visualization of the structures was performed using Mercury 2025.3.3. Displacement ellipsoids are depicted with 50 % probability. Crystallographic data for the structure reported in this paper have been deposited in the Cambridge Crystallographic Data Centre (Deposition Number: 2560662) These data can be obtained free of charge from The Cambridge Crystallographic Data Centre via www.ccdc.cam.ac.uk/data_request/cif.

2. Synthesis

General procedure 1 (GP1): Suzuki coupling

Under N_2-atmosphere the corresponding fluorenone (1 eq.), pinacolborolane **Bpin-Ar** (1 eq.), Cs_2CO_3 (3-5 eq.) and $Pd(PPh_3)_4$ (5 mol%) were dissolved in degassed THF / H_2O (10 : 1) and stirred for 18 - 72 h at 60 - 80 °C. After cooling to ambient temperature the solvent was removed under reduced pressure. The residue was dissolved in CH_2Cl_2 (50 mL) and deion. H_2O (50 mL), the phases were separated and the aqueous layer was extracted with CH_2Cl_2 (3×50 mL). The combined organic extracts were dried over $MgSO_4$ and the solvent was removed under reduced pressure. The crude product was purified via coloumn chromatography. Detailed reaction and purification conditions can be found below.

2-(4-(Decyloxy)phenyl)-4,4,5,5-tetramethyl-1,3,2-dioxaborolane (Bpin-Ar)

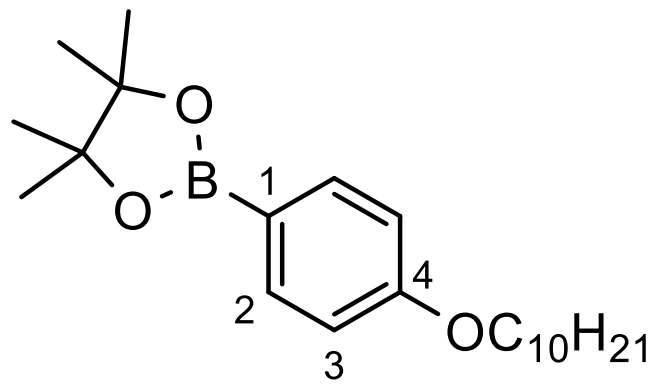

$C_{22}H_{37}BO_3$
360.28

Bpin-Ar

4-(4,4,5,5-Tetramethyl-1,3,2-dioxaborolan-2-yl)-phenol **Bpin-Ar**[1] (5.00 g, 22.72 mmol, 1 eq.) was dissolved in degassed MeCN (200 mL) under N_2-atmosphere. K_2CO_3 (9.42 g, 68.16 mmol, 3 eq.) and bromodecane (4.7 mL, 22.72 mmol, 1 eq.) were added and the reaction mixture was refluxed for 48 h. After cooling to ambient temperature the solvent was removed under reduced pressure. The residue was dissolved in EtOAc (200 mL) and deion. H_2O (100 mL), the phases were separated and the aqueous layer was extracted with EtOAc (3 × 100 mL). The combined organic layers were dried over $MgSO_4$. The solvent was removed under reduced pressure and **Bpin-Ar** was obtained as off-white solid (7.96 g, 22.09 mmol, 97 %) and used without further purification.

^{1}H NMR (400 MHz, $CDCl_3$): δ = 0.86–0.91 (m, 3H, CH_3), 1.22–1.39 (m, 24H, CH_2, $C(CH_3)_2$), 1.40–1.51 (m, 2H, $OCH_2CH_2C\mathit{H}_2$), 1.73–1.83 (m, 2H, $OCH_2C\mathit{H}_2$), 3.98 (t, J = 6.6 Hz, 2H, OCH_2), 6.86–6.91 (m, 2H, 3-H), 7.71–7.77 (m, 2H, 2-H) ppm.

^{13}C NMR (101 MHz, $CDCl_3$): δ = 14.3 (CH_3), 22.8 ($\mathit{C}H_2CH_3$), 25.0 ($C(\mathit{C}H_3)_2$), 26.2, 29.4, 29.5, 29.5, 29.7, 29.7, 32.0 (CH_2), 67.9 (OCH_2), 83.6 ($\mathit{C}(CH_3)_2$), 114.0 (C-3), 136.6 (C-2), 161.9 (C-4) ppm.

T_m = 44 °C.

The spectroscopic data are in good agreement with literature.[6]

2-Bromo-7-nitro-fluoren-9-one (NO_2-FO-Br)

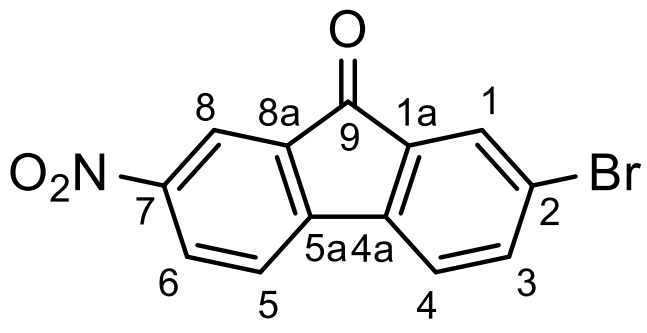

C13H6BrNO3
302.95

NO2-FO-Br

2-Bromofluorenone **H-FO-Br** (2.50 g, 9.65 mmol, 1 eq.) was suspended in deion. H_2O (30 mL) and heated to 80 °C. In a different flask conc. HNO_3 (14 M, 20 mL, 280 mmol, 29 eq.) was cooled to 0 °C and conc. H_2SO_4 (18 M, 19 mL, 347 mmol, 36 eq.) was added dropwise. Subsequently, the nitrating acid was added to the reaction mixture at 80 °C via an addition funnel. After completed addition the reaction mixture was stirred for 3 h at 120 °C. The reaction mixture was cooled to ambient temperature and poured onto ice water. The yellow solid was filtered and washed with deion. H_2O (2 × 100 mL). After recrystallization from EtOH **NO_2-FO-Br** was obtained as yellow solid (2.14 g, 9.65 mmol, 73 %).

^{1}H NMR (400 MHz, $CDCl_3$): δ = 7.55 (d, *J* = 8.0 Hz, 1H, 4-H), 7.70 (d, *J* = 8.2 Hz, 1H, 5-H), 7.74 (dd, *J* = 8.0 Hz, 1.9 Hz, 1H, 3-H), 7.89 (d, *J* = 1.8 Hz, 1H, 1-H), 8.43 (dd, *J* = 8.2 Hz, 2.2 Hz, 1H, 6-H), 8.48 (d, *J* = 2.2 Hz, 1H, 8-H) ppm.

^{13}C NMR (101 MHz, $CDCl_3$): δ = 120.0 (C-8), 121.0, 123.2 (4-C, 5-C), 125.5 (2-C), 128.5 (1-C), 130.4 (6-C), 134.7 (C-1a), 136.6 (C-8a), 138.1(C-3), 141.0 (C-4a), 149.1 (C-7, C-5a), 189.7 (C-9) ppm.

T_m = 233 °C

The spectroscopic data are in good agreement with literature.[7]

2,7-Diiodo-9*H*-fluoren-9-one (I-FO-I)

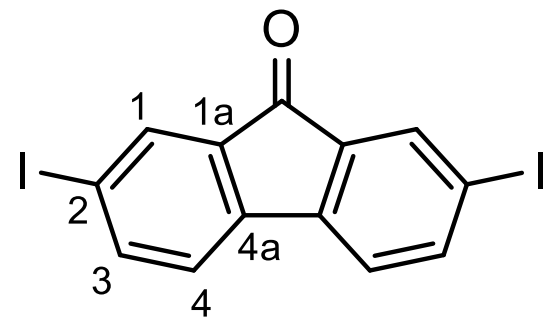

C13H6I2N
431.85

I-FO-I

A two-necked flask equipped with a reflux condenser was filled with AcOH (38 mL) and H_2O (1 mL). Subsequently, conc. H_2SO_4 (18 M, 7.7 mL, 139 mmol, 5 eq.), fluorenone (5.00 g, 27.8 mmol, 1 eq.) and H_5IO_6 (3.16 g, 13.9 mmol, 0.5 eq.) were added and the reaction mixture

was heated to 70 °C. Iodine (14.23 g, 56.1 mmol, 2.02 eq.) was added and the reaction mixture was stirred for 18 h at 85 °C. After cooling to ambient temperature the reaction mixture was poured onto deion. H_2O and saturated aqueous $Na_2S_2O_5$ solution was added until a color change from brown to yellow occurred. Then, CH_2Cl_2 (200 mL) was added, the phases were separated and the aqueous layer was extracted with CH_2Cl_2 (3 × 100 mL). The combined organic extracts were dried over $MgSO_4$ and the solvent was removed under reduced pressure. After recrystallization from AcOH **I-FO-I** was obtained as yellow solid (9.59 g, 22.2 mmol, 80 %).

^{1}H NMR (400 MHz, $CDCl_3$): δ = 7.27 (d, *J* = 7.9 Hz, 2H, 4-H), 7.83 (dd, *J* = 7.8 Hz, 1.6 Hz, 2H, 3-H), 7.96 (d, *J* = 1.6 Hz, 2H, 1-H) ppm.

^{13}C NMR (101 MHz, $CDCl_3$) δ = 94.6 (C-2), 122.3 (C-4), 133.7 (C-1), 135.0 (C-1a), 143.1 (C-4a), 143.6 (C-3), 191.1 (C=O) ppm.

T_m = 208 °C

The spectroscopic data are in good agreement with literature.[8]

7-Iodo-9-oxo-9*H*-fluorene-2-carbonitrile (CN-FO-I)

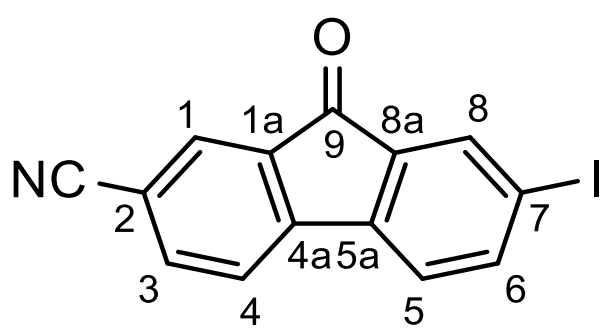

$C_{14}H_6INO$
330.94

CN-FO-I

In a pressure vial diiodofluorenone **I-FO-I** (100 mg, 231 µmol, 1 eq.), 2-aminopyridine (4.36 mg, 46.3 µmol, 20 mol%), Ag_2CO_3 (128 mg, 463 µmol, 2 eq.), $Cu(NO_3)_2 \cdot 3\,H_2O$ (11.19 mg, 46.3 µmol, 20 mol%) und NH_4HCO_3 (36.6 mg, 463 µmol, 2 eq.) were evacuated for 10 min. Under N_2 atmosphere abs. DMF (4 ml) was added. The pressure vial was evacuated for some seconds and backfilled with O_2 (this process was repeated five times). The reaction mixture was stirred for 18 h at 150 C. After cooling to ambient temperature EtOAc (50 ml) and 3 M aq. NH_3 solution (30 ml) were added and the mixture was filtered over a short Celite® pad. The phases were separated and the aqueous layer was extracted with EtOAc (3 × 30 mL). The combined organic extracts were dried over Na_2SO_4 and the solvent was removed under reduced pressure. The crude product was purified via column chromatography (silica gel, eluent: CH_2Cl_2 / petroleum ether 3 : 2, R_f = 0.36). **CN-FO-I** was obtained as yellow solid (30 mg, 91 µmol, 39 %)

^{1}H NMR (400 MHz, $CDCl_3$): δ = 7.38 (d, *J* = 7.9 Hz, 1H, 5-H), 7.65 (d, *J* = 7.8 Hz, 1H, 4-H), 7.82 (dd, *J* = 7.8 Hz, 1.5 Hz, 1H, 3-H), 7.90–7.91 (m, 1H, 1-H), 7.93 (dd, *J* = 7.9 Hz, 1.6 Hz, 1H, 6-H), 8.06 (d, *J* = 1.6 Hz, 1H, 8-H) ppm.

^{13}C NMR (101 MHz, $CDCl_3$): δ = 96.4 (C-7), 113.3 (C-2), 118.1 (CN), 121.2 (C-4), 123.1 (C-5), 127.9 (C-1), 133.8 (C-1a), 134.2 (C-8), 135.7 (C-8a), 138.9 (C-3), 142.0 (C-5a), 144.0 (C-6), 147.5 (C-4a), 190.1 (C-9) ppm.

FT-IR (ATR): $\tilde{\nu}$ = 3082 (w), 2921 (w), 2854 (w), 2232 (w), 2189 (w), 2032 (w), 1716 (vs), 1614 (m), 1593 (m), 1452 (w), 1408 (w), 1255 (w), 1173 (m), 1120 (w), 930 (w), 869 (w), 838 (m), 783 (m), 710 (w), 649 (w), 612 (w), 585 (w), 516 (m), 469 (w), 437 (w) cm^{-1}.

HRMS (ESI): *m*/*z* for $C_{14}H_6INO$ calculated: 330.9500 [M], found: 330.9502.

T_m = 231 °C

2-(4-(Decyloxy)phenyl)-fluoren-9-one (H-FO-Ar)

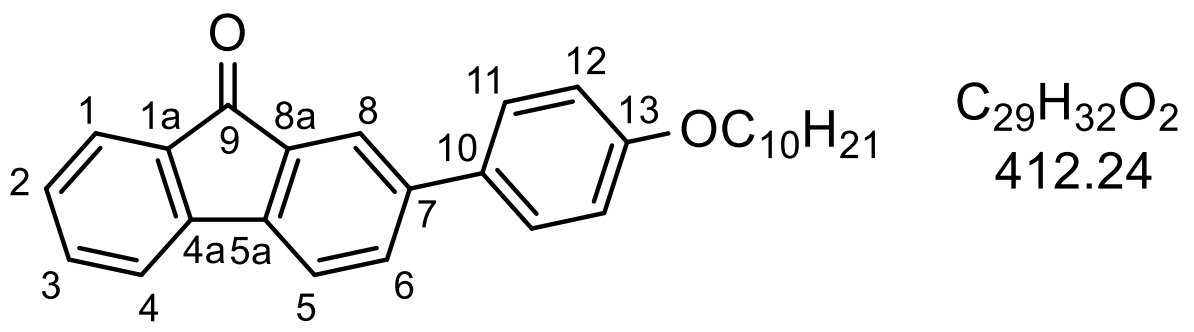

H-FO-Ar

Preparation according to GP1. 2-bromofluorenone **H-FO-Br** (1.00 g, 3.86 mmol, 1 eq.), pinacol-borolane **Bpin-Ar** (1.39 g, 3.86 mmol, 1 eq.), Cs_2CO_3 (3.77 g, 11.6 mmol, 3 eq.), $Pd(PPh_3)_4$ (199 mg, 193 µmol, 5 mol%), solvent: THF / H_2O (150 mL : 15 mL), reaction conditions: 18 h at 80 °C. Column chromatography (silica gel, eluent: petroleum ether / CH_2Cl_2 2 : 1, R_f = 0.19). **H-FO-Ar** was obtained as yellow solid (1.50 g, 3.64 mmol, 94 %).

^{1}H NMR (700 MHz, $CDCl_3$): δ = 0.89 (t, *J* = 7.0 Hz, 3H, CH_3), 1.23–1.39 (m, 12H, CH_2), 1.45–1.51 (m, 2H, $OCH_2CH_2CH_2$), 1.81 (p, *J* = 6.8 Hz, 2H, OCH_2CH_2), 4.01 (t, *J* = 6.6 Hz, 2H, OCH_2), 6.98 (d, *J* = 8.4 Hz, 2H, 12-H), 7.29 (t, *J* = 7.3 Hz, 1H, 2-H), 7.49 (t, *J* = 7.4 Hz, 1H, 3-H), 7.52–7.57 (m, 4H, 4-H, 5-H, 11-H), 7.65–7.70 (m, 2H, 1-H, 6-H), 7.87 (d, *J* = 1.8 Hz, 1H, 8-H) ppm.

^{13}C NMR (176 MHz, $CDCl_3$): δ = 14.3 (CH_3), 22.8, 26.2, 29.4, 29.5, 29.6, 29.7, 29.7, 32.0 (CH_2), 68.3 (OCH_2), 115.1 (C-12), 120.4 (C-4), 120.8 (C-5), 122.6 (C-8), 124.5 (C-1), 128.0 (C-11), 129.0 (C-2), 132.2 (C-10), 132.7 (C-6), 134.6 (C-1a), 134.9 (C-3), 135.0 (C-8a), 142.1 (C-7), 142.7 (C-5a), 144.6 (C-4a), 159.4 (C-13), 194.2 (C-9) ppm.

FT-IR (ATR): $\tilde{\nu}$ = 3066 (w), 2923 (vs), 2852 (s), 2005 (w), 1718 (vs), 1599 (s), 1522 (m), 1479 (w), 1467 (m), 1454 (s), 1389 (w), 1303 (m), 1285 (m), 1250 (s), 1216 (w), 1185 (m), 1150 (w), 1114 (w), 1071 (w), 987 (w), 940 (w), 908 (w), 824 (s), 769 (m), 743 (m), 681 (w), 649 (w), 610 (w), 528 (w), 475 (w) cm^{-1}.

HRMS (APCI): *m*/*z* for $C_{29}H_{33}O_2^+$ calculated: 413.2475 $[M+H]^+$, found: 413.2466.

T_m = 94 °C (DSC).

2-Bromo-7-(4-(decyloxy)phenyl)-fluoren-9-one (Br-FO-Ar)

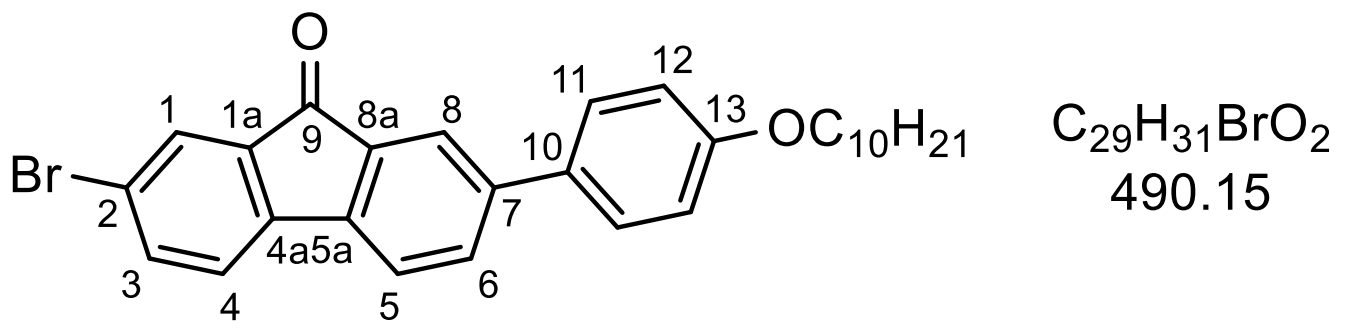

Br-FO-Ar

Preparation according to GP1. 2,7-Dibromofluorenone **Br-FO-Br** (1.00 g, 2.78 mmol, 1 eq.), pinacol-borolane **Bpin-Ar** (0.94 g, 2.78 mmol, 1 eq.), Cs_2CO_3 (4.52 g, 13.88 mmol, 5 eq.), $Pd(PPh_3)_4$ (160 mg, 139 µmol, 5 mol%), solvent: THF / H_2O (150 mL : 15 mL) reaction conditions: 72 h at 80 °C. Column chromatography (silica gel, eluent: petroleum ether / CH_2Cl_2 3 : 1 → 2 : 1, R_f = 0.38 (2 : 1)). **Br-FO-Ar** was obtained as orange solid (759 mg, 1.54 mmol, 56 %).

^{1}H NMR (700 MHz, $CDCl_3$): δ = 0.89 (t, *J* = 7.0 Hz, 3H, CH_3), 1.24–1.40 (m, 12H, CH_2), 1.44–1.50 (m, 2H, $OCH_2CH_2CH_2$), 1.78–1.83 (m, 2H, OCH_2CH_2), 4.00 (t, *J* = 6.6 Hz, 2H, OCH_2), 6.96–6.99 (m, 2H, 12-H), 7.39 (d, *J* = 7.9 Hz, 1H, 4-H), 7.52–7.54 (m, 3H, 5-H, 11-H), 7.61 (dd, *J* = 7.9 Hz, 1.9 Hz, 1H, 3-H), 7.68 (dd, *J* = 7.7 Hz, 1.8 Hz, 1H, 6-H), 7.77 (d, *J* = 1.8 Hz, 1H, 1-H), 7.85 (d, *J* = 1.7 Hz, 1H, 8-H) ppm.

^{13}C NMR (176 MHz, $CDCl_3$): δ = 14.3 (CH_3), 22.8, 26.2, 29.4, 29.5, 29.5, 29.7, 29.7, 32.0 (CH_2), 68.3 (OCH_2), 115.1 (C-12), 121.0 (C-5), 121.8 (C-4), 122.8 (C-1a), 122.9 (C-8), 127.8 (C-1), 128.0 (C-11), 132.0 (C-10), 133.0 (C-6), 134.6 (C-7), 136.2 (C-2), 137.3 (C-3), 141.9 (C-5a), 142.5 (C-8a), 143.2 (C-4a), 159.5 (C-13), 192.7 (C-9) ppm.

FT-IR (ATR): $\tilde{\nu}$ = 3033 (w), 2954 (m), 2923 (s), 2852 (m), 2251 (w), 2209 (w), 2172 (w), 2155 (w), 2129 (w), 2069 (w), 2047 (w), 2030 (w), 1973 (w), 1894 (w), 1713 (vs), 1607 (m), 1596 (m), 1518 (m), 1467 (m), 1453 (s), 1435 (m), 1408 (w), 1304 (w), 1288 (m), 1254 (s), 1209 (w), 1184 (m), 1150 (w), 1120 (w), 1096 (w), 1051 (w), 1020 (w), 906 (s), 822 (s), 784 (s), 733 (s), 650 (m), 627 (w), 587 (w), 544 (w), 529 (w), 491 (w), 435 (w), 417 (w) cm^{-1}.

HRMS (ESI): *m*/*z* for $C_{29}H_{32}BrO_2^+$ calculated: 491.1580 $[M+H]^+$, found: 491.1562.

T_m = 90 °C (DSC).

7-(4-(Decyloxy)phenyl)-2-nitro-fluoren-9-one (NO_2-FO-Ar)

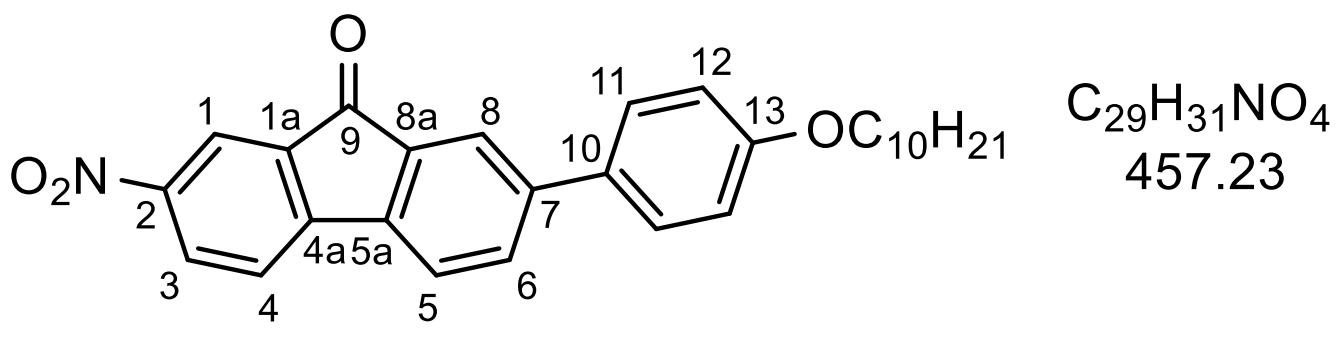

NO_2-FO-Ar

Preparation according to GP1. **NO_2-FO-Br** (1.00 g, 3.29 mmol, 1 eq.), pinacolborolane **Bpin-Ar** (1.18 g, 3.29 mmol, 1 eq.), Cs_2CO_3 (5.36 g, 16.44 mmol, 5 eq.), $Pd(PPh_3)_4$ (190 mg, 164 µmol, 5 mol%), solvent: THF / H_2O (150 mL : 15 mL), reaction conditions: 18 h at 80 °C. Column chromatography (silica gel, eluent: petroleum ether / CH_2Cl_2 1 : 1, R_f = 0.33). **NO_2-FO-Ar** was obtained as orange solid (1.23 g, 2.69 mmol, 82 %).

^{1}H NMR (400 MHz, $CDCl_3$): δ = 0.86–0.92 (m, 3H, CH_3), 1.24–1.42 (m, 12H, CH_2), 1.42–1.53 (m, 2H, $OCH_2CH_2C\mathit{H}_2$), 1.71–1.90 (m, 2H, $OCH_2C\mathit{H}_2$), 4.01 (t, *J* = 6.6 Hz, 2H, OCH_2), 6.96–7.02 (m, 2H, 12-H), 7.53–7.59 (m, 2H, 11-H), 7.68 (d, *J* = 8.0 Hz, 2H, 4-H, 5-H), 7.78 (dd, *J* = 7.8 Hz, 1.7 Hz, 1H, 6-H), 7.96 (d, *J* = 1.7 Hz, 1H, 8-H), 8.41 (dd, *J* = 8.2 Hz, 2.2 Hz, 1H, 3-H), 8.47 (d, *J* = 2.1 Hz, 1H, 1-H) ppm.

^{13}C NMR (101 MHz, $CDCl_3$): δ = 14.3 (CH_3), 22.8, 26.2, 29.4, 29.5, 29.5, 29.7, 29.7, 32.0 (CH_2), 68.4 (OCH_2), 115.2 (C-12), 119.7 (C-1), 120.7, 122.3 (C-4, C-5), 123.2 (C-8), 128.1 (C-11), 130.2 (C-3), 131.4 (C-10), 133.3 (C-6), 135.4, 135.9 (C-1a, C-8a), 140.3 (C-5a), 144.2 (C-7), 148.6 (C-2), 150.0 (C-4a), 159.9 (C-13), 191.2 (C-9) ppm.

FT-IR (ATR): $\tilde{\nu}$ = 3424 (w), 3096 (w), 3038 (w), 2920 (s), 2852 (s), 2015 (w), 1718 (vs), 1597 (s), 1517 (vs), 1455 (s), 1443 (m), 1422 (w), 1392 (w), 1338 (vs), 1307 (m), 1292 (m), 1280 (m), 1251 (s), 1215 (m), 1181 (m), 1153 (m), 1124 (m), 1110 (m), 1059 (m), 1021 (m), 968 (w), 918 (w), 876 (w), 824 (s), 798 (w), 786 (m), 765 (w), 733 (m), 705 (w), 678 (w), 650 (w), 637 (w), 592 (w), 558 (w), 529 (w), 482 (w), 417 (w) cm^{-1}.

HRMS (CI): *m*/*z* for $C_{29}H_{32}NO_4^+$ calculated: 458.2326 $[M+H]^+$, found: 458.2328.

T_m = 124 °C (DSC).

2-Amino-7-(4-(decyloxy)phenyl)-fluoren-9-one (NH_2-FO-Ar)

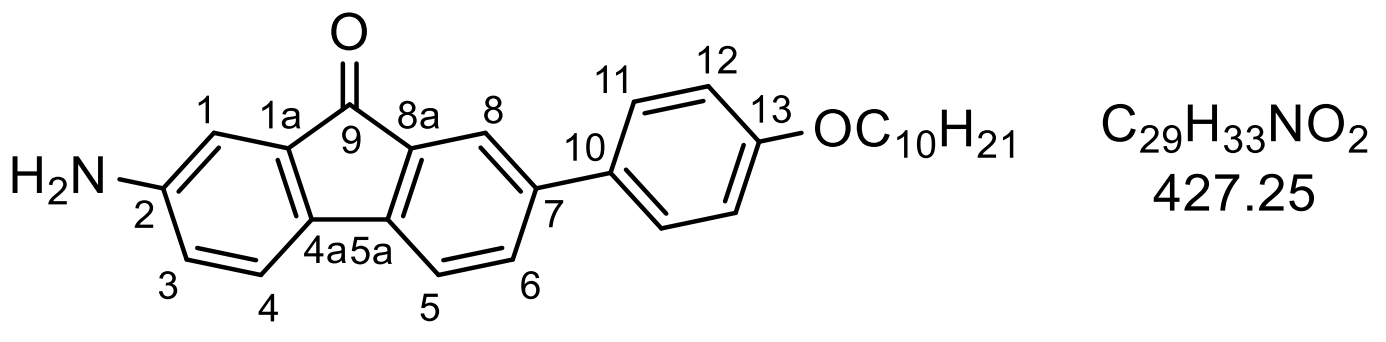

NH_2-FO-Ar

Under N_2 atmosphere nitrofluorenone **NO_2-FO-Ar** (100 mg, 219 µmol, 1 eq.) was dissolved in abs. THF (100 ml). NH_4HCO_2 (138 mg, 2.19 mmol, 10 eq.) and Pd/C (10 wt%, 11.6 mg, 10.9 µmol, 5 mol%) were added and the reaction mixture was stirred for 1 h at 60 °C. After cooling to ambient temperature, the reaction mixture was filtered over Celite® and the solvent was removed under reduced pressure. The crude product was purified via column chromatography (silica gel, eluent: petroleum ether / EtOAc / CH_2Cl_2 3 : 1 : 1 + 1 % NEt_3, R_f = 0.45). **NH_2-FO-Ar** was obtained as violet solid (49 mg, 115 µmol, 52 %).

^{1}H NMR (700 MHz, $CDCl_3$): δ = 0.89 (t, J = 7.2 Hz, 5.5 Hz, 3H, CH_3), 1.23–1.41 (m, 12H, CH_2), 1.44–1.51 (m, 2H, $OCH_2CH_2CH_2$), 1.78–1.84 (m, 2H, OCH_2CH_2), 3.87 (brs, 2H, NH_2), 4.00 (t, J = 6.7 Hz, 5.4 Hz, 2H, OCH_2), 6.71–6.76 (m, 1H, 3-H), 6.94–7.01 (m, 3H, 1-H, 12-H), 7.29 (d, J = 8.3 Hz, 3.7 Hz, 1H, 4-H), 7.36–7.40 (m, 1H, 5-H), 7.50–7.55 (m, 2H, 11-H), 7.58–7.63 (m, 1H, 6-H), 7.78 (s, 1H, 8-H) ppm.

^{13}C NMR (176 MHz, $CDCl_3$): δ = 14.3 (CH_3), 22.8, 26.2, 29.4, 29.5, 29.6, 29.7, 29.7, 32.0 (CH_2), 68.3 (OCH_2), 111.3 (C-1), 115.0 (C-12), 119.5 (C-5), 119.8 (C-3), 121.5 (C-4), 122.6 (C-8), 127.8 (C-11), 132.5 (C-10), 132.8 (C-6), 134.8 (C-8a), 134.9 (C-4a), 136.4 (C-1a), 140.3 (C-7), 143.8 (C-5a), 147.6 (C-2), 159.1 (C-13), 194.5 (C-9) ppm.

FT-IR (ATR): $\tilde{\nu}$ = 3839 (w), 3791 (w), 3746 (w), 3679 (w), 3456 (w), 3358 (m), 2922 (s), 2852 (m), 2564 (w), 2515 (w), 2428 (w), 2357 (w), 2295 (w), 2209 (w), 2192 (w), 2180 (w), 2145 (w), 2107 (w), 2057 (w),2039 (w), 2030 (w), 2009 (w), 1978 (w), 1948 (w), 1914 (w), 1904 (w), 1882 (w), 1704 (s), 1603 (s), 1520 (m), 1493 (w), 1466 (vs), 1390 (w), 1364 (w), 1314 (m), 1287 (w), 1272 (m), 1248 (s), 1185 (m), 1143 (w), 1118 (w), 1070 (w), 1025 (w), 973 (w), 904 (w), 823 (s), 785 (m), 721 (w), 650 (w), 600 (w), 541 (w), 502 (w), 419 (w), 408 (w) cm^{-1}.

HRMS (ESI): m/z for $C_{29}H_{34}NO_2^+$ calculated: 428.2584 $[M+H]^+$, found: 428.2571.

T_m = 224 °C (DSC).

7-(4-(Decyloxy)phenyl)-9-oxo-9*H*-fluorene-2-carbonitrile (CN-FO-Ar)

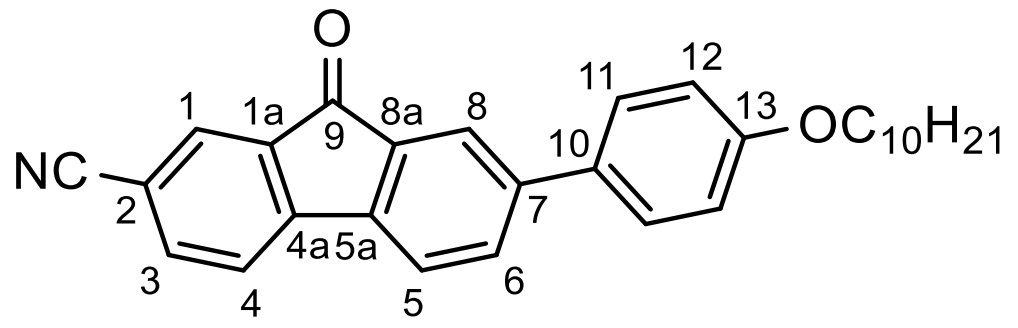

$C_{30}H_{31}NO_2$
437.24

CN-FO-Ar

Preparation according to GP1. **CN-FO-I** (28 mg, 84.6 µmol, 1 eq.), pinacolborolane **Bpin-Ar** (30.5 mg, 84.6 µmol, 1 eq.), Cs_2CO_3 (82.7 mg, 254 µmol, 3 eq.), $Pd(PPh_3)_4$ (4.36 mg, 4.23 µmol, 5 mol%), solvent: THF / H_2O (10:1, 40 mL) reaction conditions: 18 h at 60 °C. Column chromatography (silica gel, eluent: CH_2Cl_2 / petroleum ether 3 : 2, R_f = 0.39). **CN-FO-Ar** was obtained as yellow solid (32 mg, 73.1 µmol, 86 %).

^{1}H NMR (400 MHz, $CDCl_3$): δ = 0.86–0.91 (m, 3H, CH_3), 1.22–1.42 (m, 12H, CH_2), 1.43–1.53 (m, 2H, $OCH_2CH_2CH_2$), 1.76–1.86 (m, 2H, OCH_2CH_2), 4.01 (t, *J* = 6.5 Hz, 2H, OCH_2), 6.97–7.01 (m, 2H, 12-H), 7.53–7.58 (m, 2H, 11-H), 7.64 (dd, *J* = 7.8 Hz, 2.6 Hz, 2H, 4-H, 5-H), 7.76 (dd, *J* = 7.8 Hz, 1.8 Hz, 1H, 6-H), 7.80 (dd, *J* = 7.7 Hz, 1.5 Hz, 1H, 3-H), 7.90 (d, *J* = 1.5 Hz, 1H, 1-H), 7.93 (d, *J* = 1.7 Hz, 1H, 8-H) ppm.

^{13}C NMR (176 MHz, $CDCl_3$): δ = 14.3 (CH_3), 22.8, 26.2, 29.4, 29.5, 29.5, 29.7, 29.7, 32.0 (CH_2), 68.3 (OCH_2), 112.4 (C-2), 115.2 (C-12), 118.4 (CN), 120.9 (C-4), 122.0 (C-5), 123.1 (C-8), 127.7 (C-1), 128.1 (C-11), 131.6 (C-10), 133.3 (C-6), 135.0, 135.1 (C-1a, C-8a), 138.8 (C-3), 140.8 (C-5a), 144.0 (C-7), 148.3 (C-4a), 159.8 (C-13), 191.7 (C-9) ppm.

FT-IR (ATR): $\tilde{\nu}$ = 2956 (m), 2921 (vs), 2852 (s), 2230 (m), 2197 (w), 2183 (w), 2160 (w), 1718 (vs), 1605 (s), 1520 (m), 1467 (s), 1393 (w), 1308 (m), 1279 (m), 1255 (s), 1181 (m), 1126 (m), 1024 (m), 820 (m), 785 (s), 655 (w), 522 (w) cm^{-1}.

HRMS (EI): *m*/*z* for $C_{30}H_{31}NO_2$ calculated: 437.2349 [M], found: 437.2349.

T_m = 142 °C (DSC).

3. Mesomorphic properties

3.1. POM results

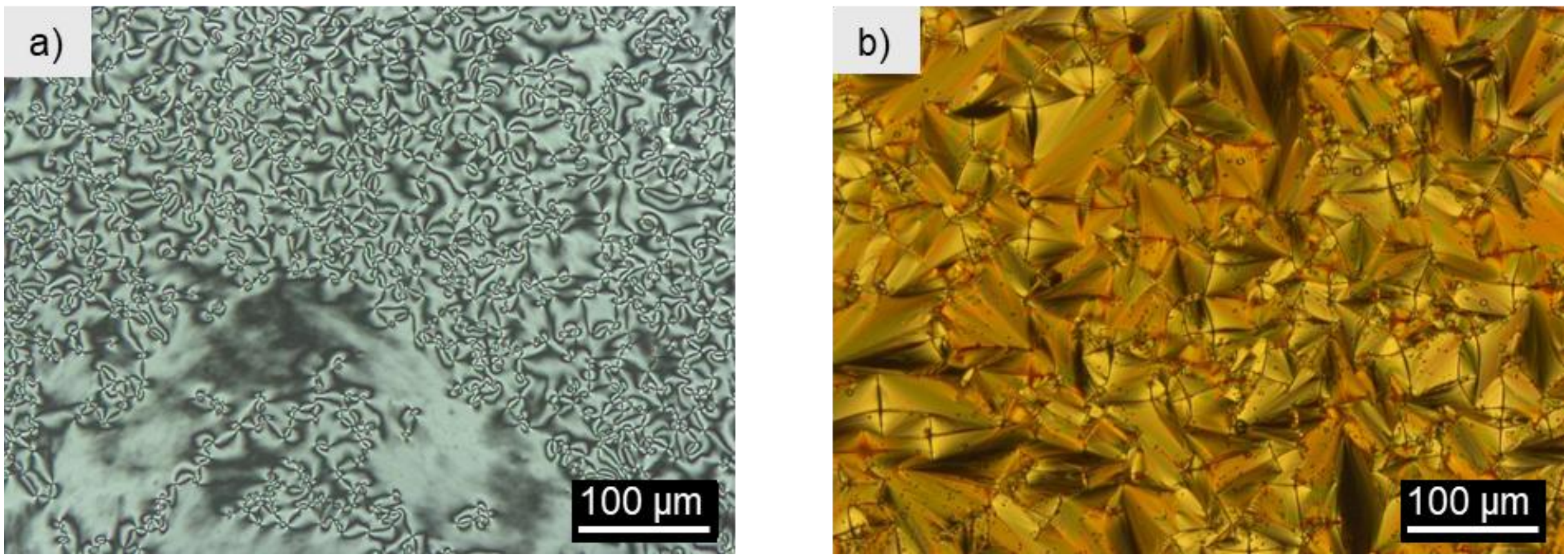

Figure S1: POM microphotography obtained upon cooling (5 K·min^{-1}) of a) **H-FO-Ar** at 72 °C, b) **Br-FO-Ar** at 205 °C.

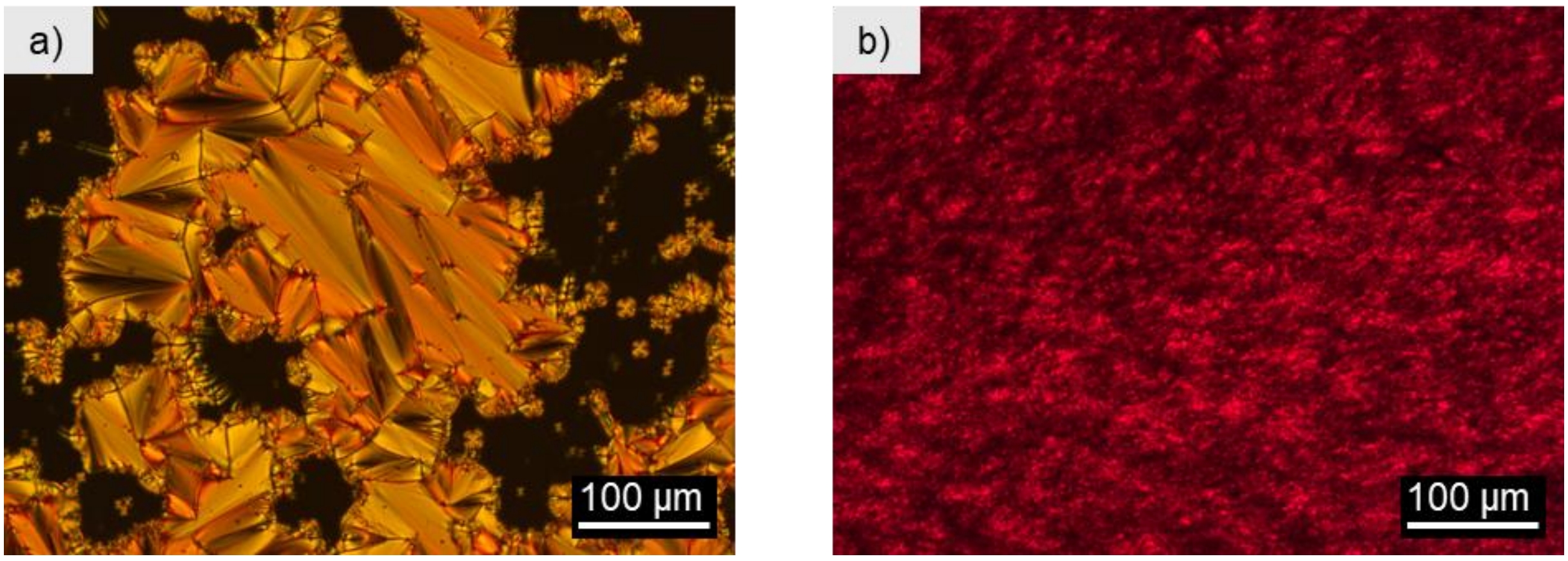

Figure S2: POM microphotography obtained upon cooling (5 K·min^{-1}) of a) **NO_2-FO-Ar** at 215 °C, b) **NH_2-FO-Ar** at 217 °C.

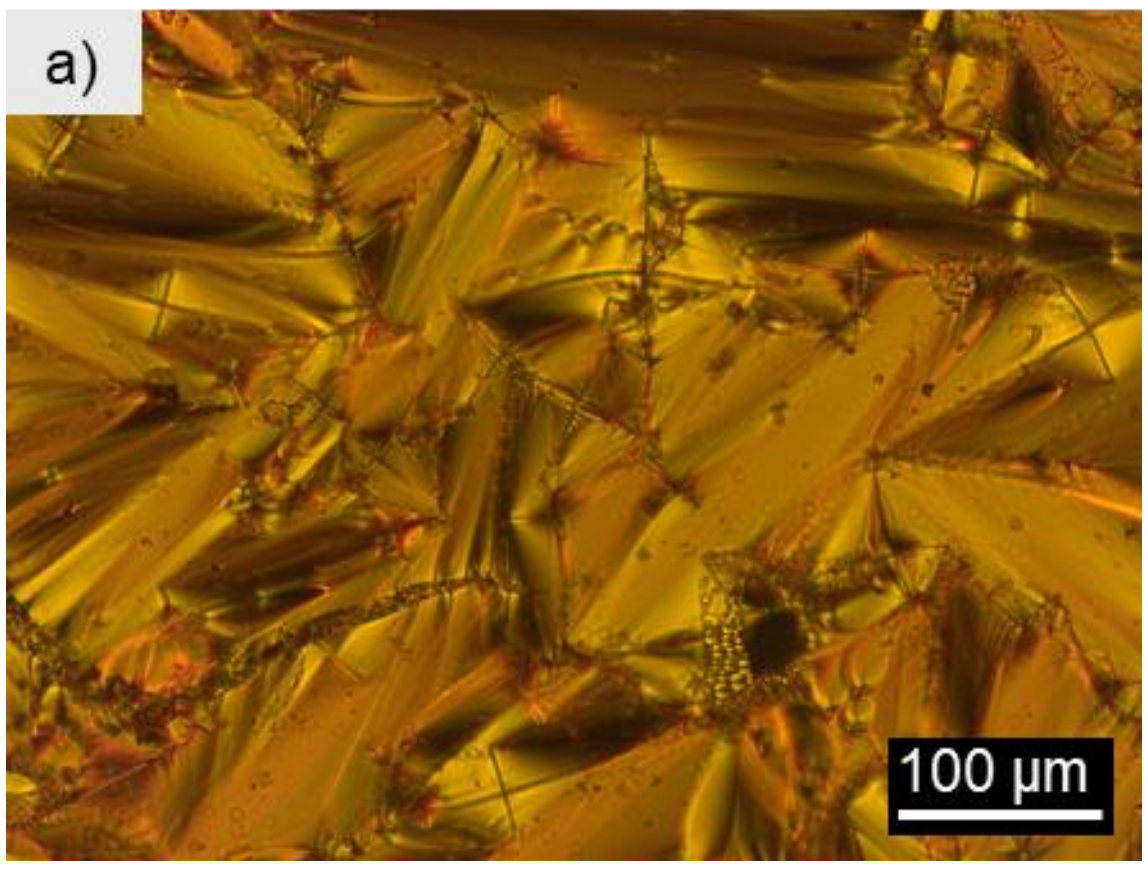

Figure S3: POM microphotography obtained upon cooling (5 K·min^{-1}) of a) **CN-FO-Ar** at 195 °C.

3.2. DSC results

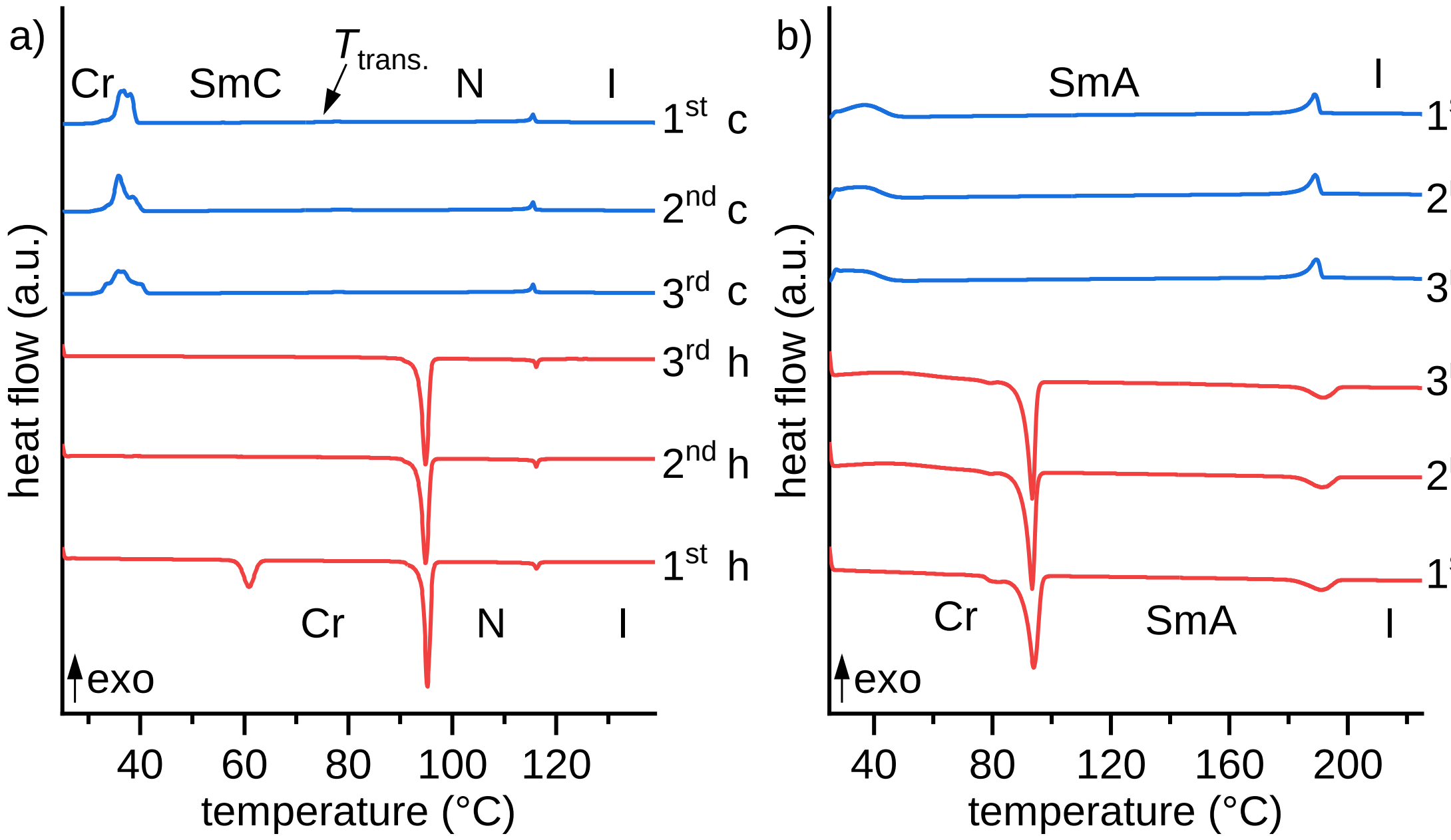

Figure S4: DSC thermogram of a) **H-FO-Ar** (heating rate 10 K/min) and b) **Br-FO-Ar** (heating rate 10 K/min); h: heating, c: cooling.

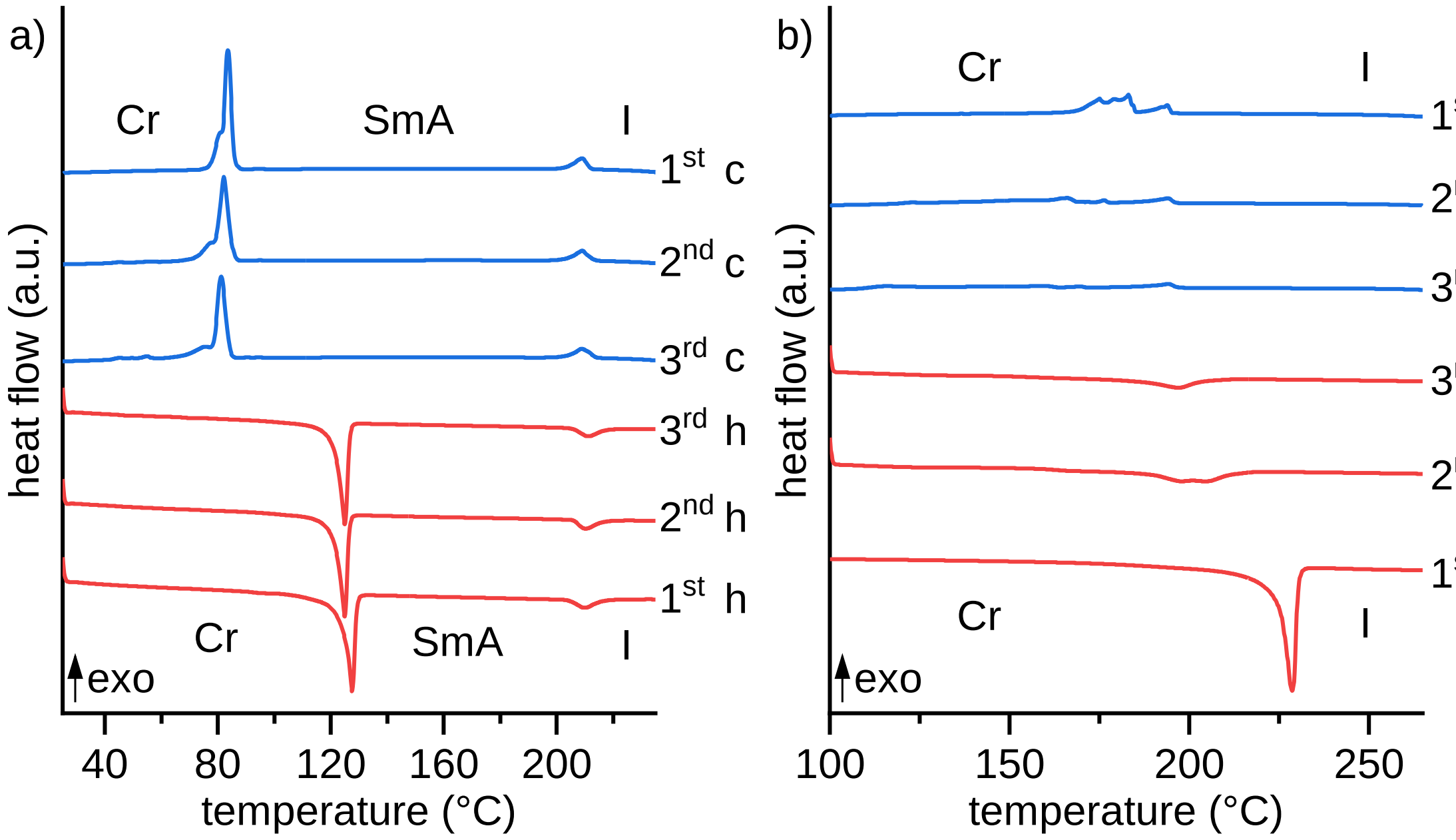

Figure S5: DSC thermogram of a) **NO_2-FO-Ar** and b) **NH_2-FO-Ar**; heating rate 10 K/min, h: heating, c: cooling.

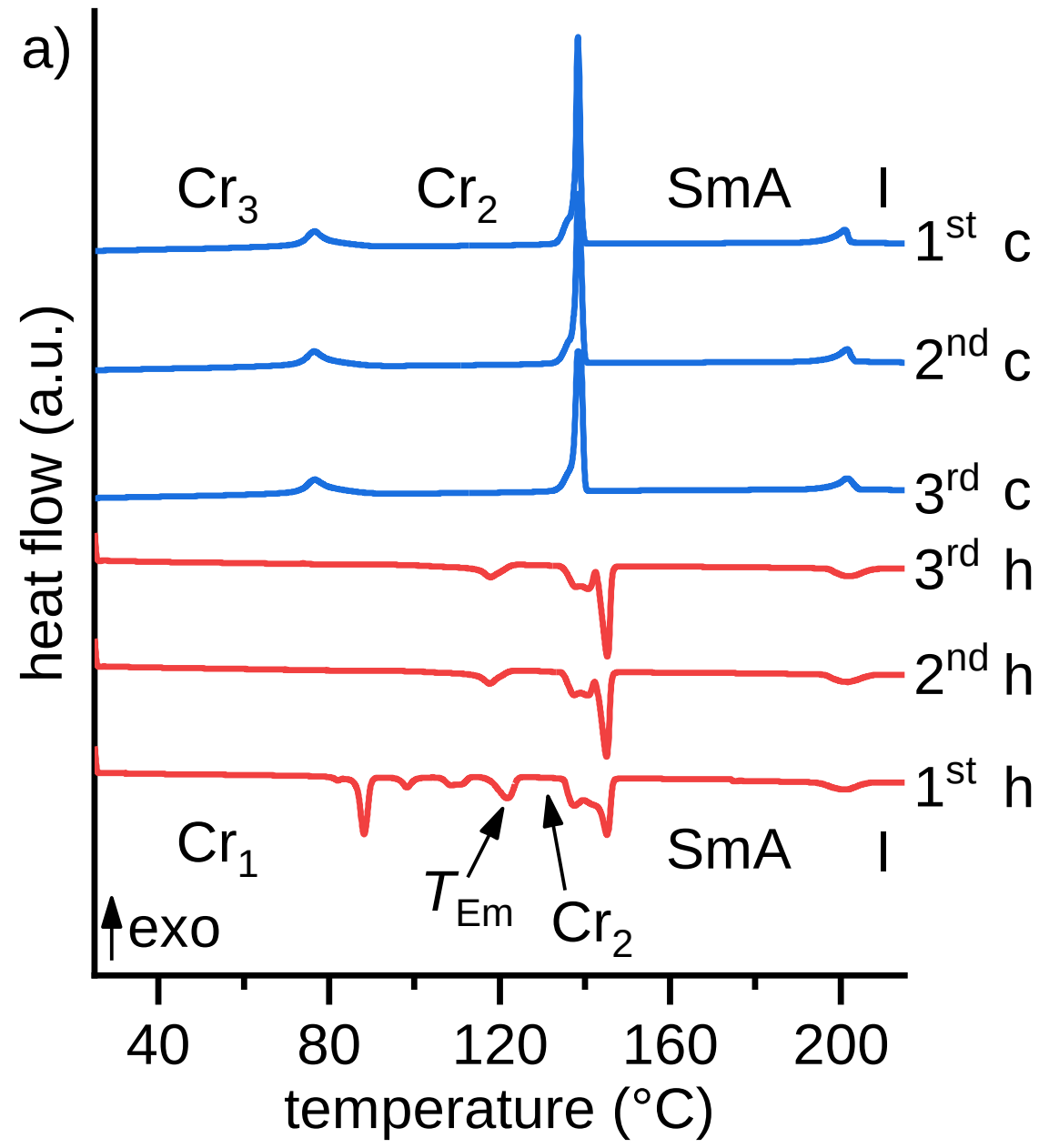

Figure S6: DSC thermogram of **CN-FO-Ar** heating rate 10 K/min, T_{Em}: change of emission color h: heating, c: cooling.

Table S1: Phase transition temperatures and -enthalpies.

Compound	Phase transitions / °C (ΔH / kJ·mol^{-1})	Cycle
**H-FO-Ar**	Cr 38 (14.1) SmC 79 (0.1) N 116 (0.7) I	2. cooling
**Br-FO-Ar**	Cr 46 (9.2) SmA 191 (4.3) I	2. cooling
**NO_2-FO-Ar**	Cr 85 (21.4) SmA 213 (3.0) I	2. cooling
**NH_2-FO-Ar**	Cr 224 (-34.6) I	1. heating
**CN-FO-Ar**	Cr_1 81 (5.0) Cr_2 140 (17.7) SmA 203 (2.7) I	2. cooling

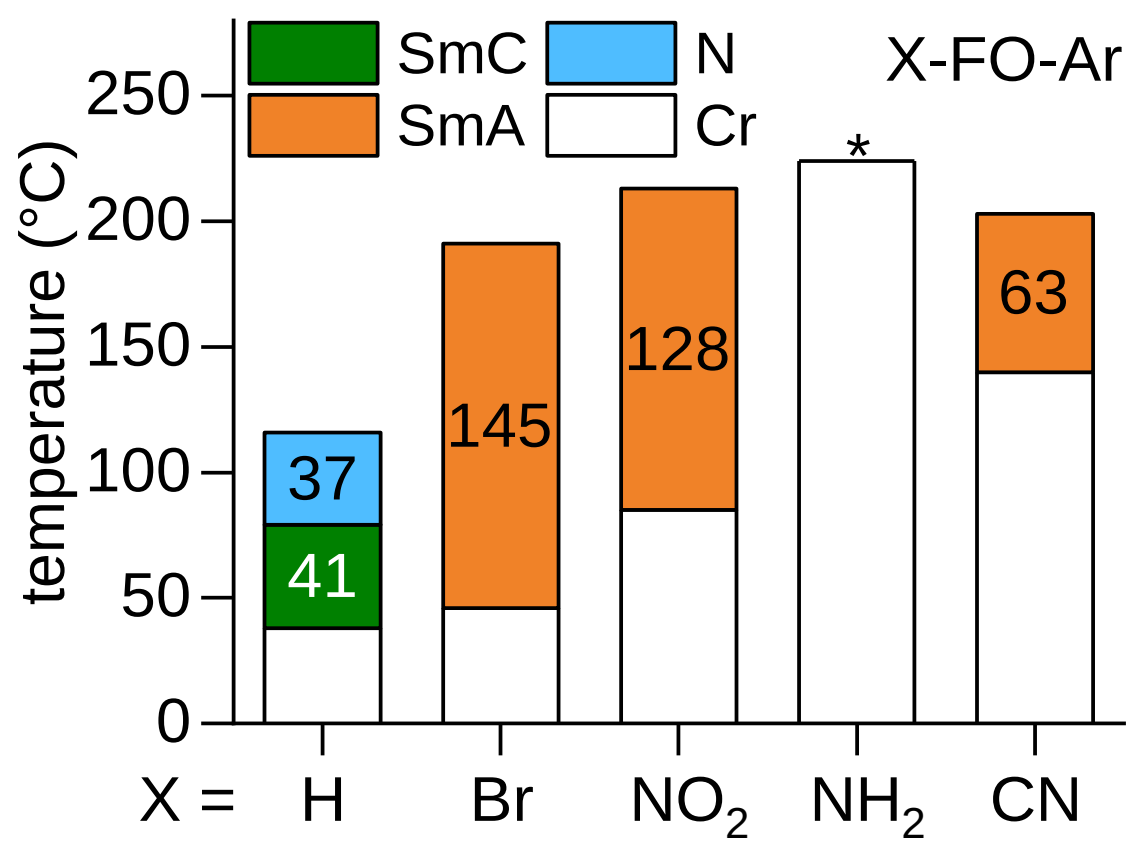

Figure S7: Phase widths determined via DSC from the second cooling cycle (* determined from the first heating cycle due to decomposition).

3.3. XRD results

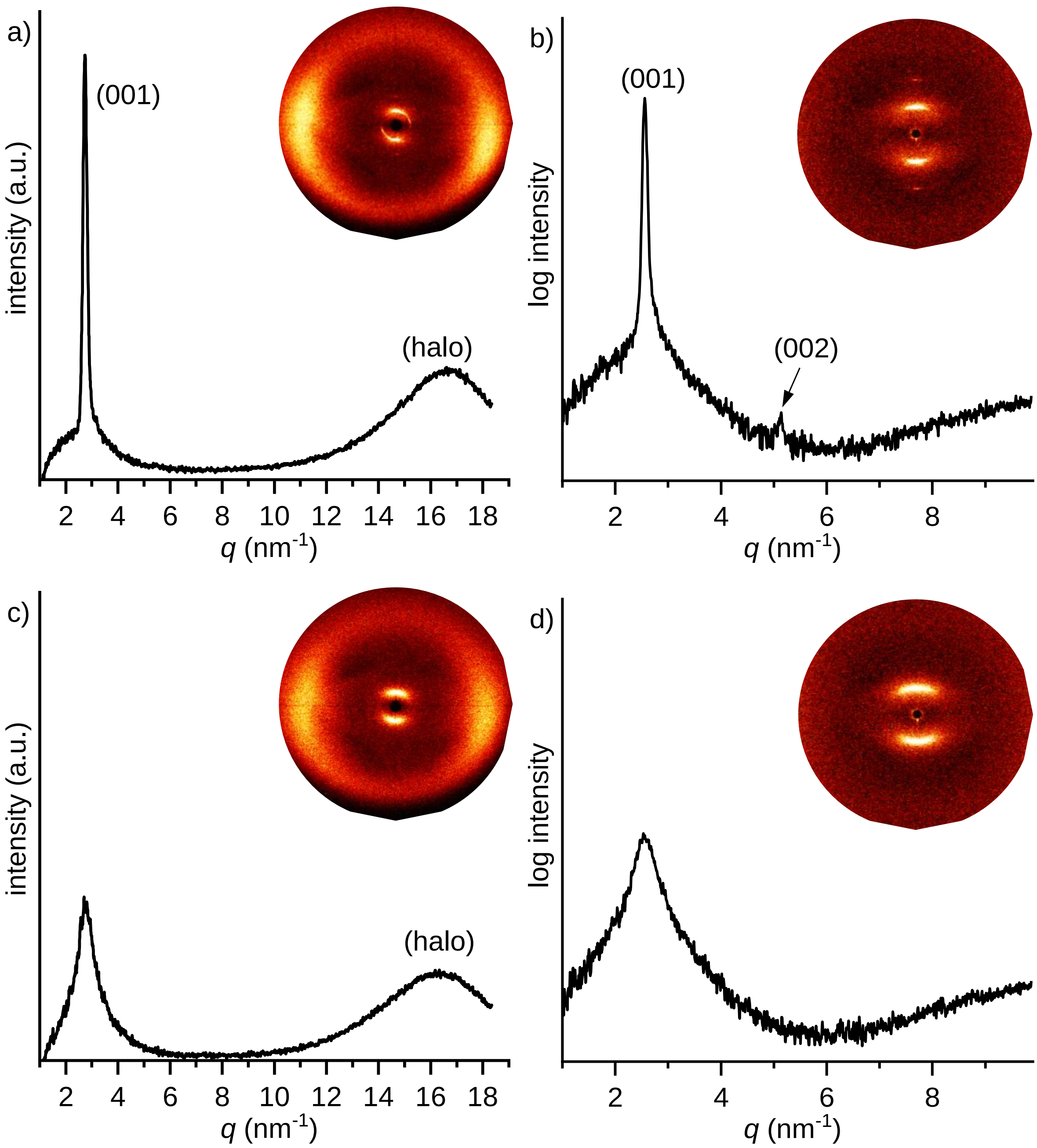

Figure S8: Diffractograms of **H-FO-Ar** with diffraction patterns a) WAXS at 90 °C, b) SAXS at 90 °C, c) WAXS at 50 °C, d) SAXS at 50 °C.

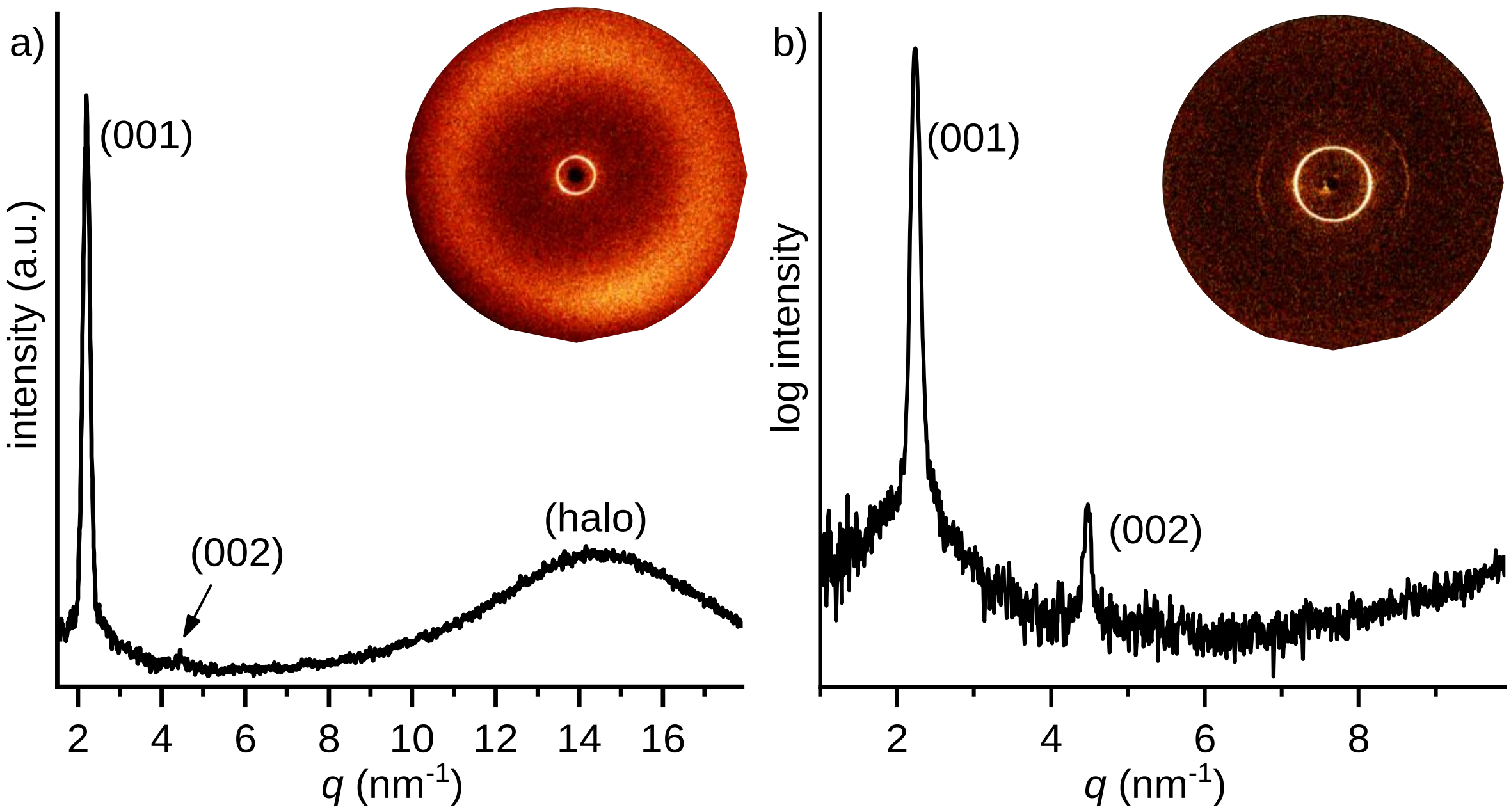

Figure S9: Diffractograms of **Br-FO-Ar** at 140 °C with diffraction patterns a) WAXS, b) SAXS.

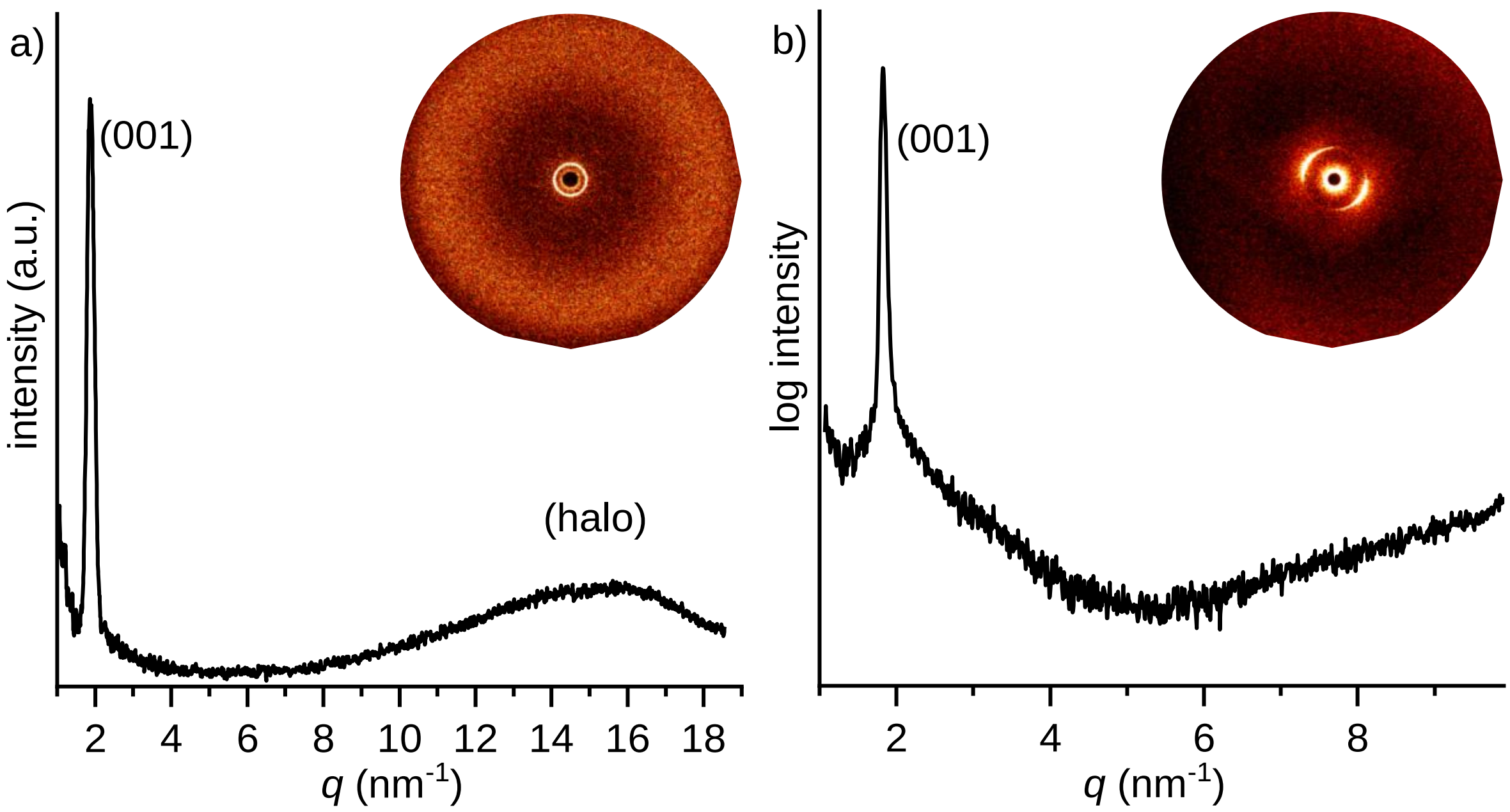

Figure S10: Diffractograms of **NO_2-FO-Ar** at 169 °C with diffraction patterns a) WAXS, b) SAXS.

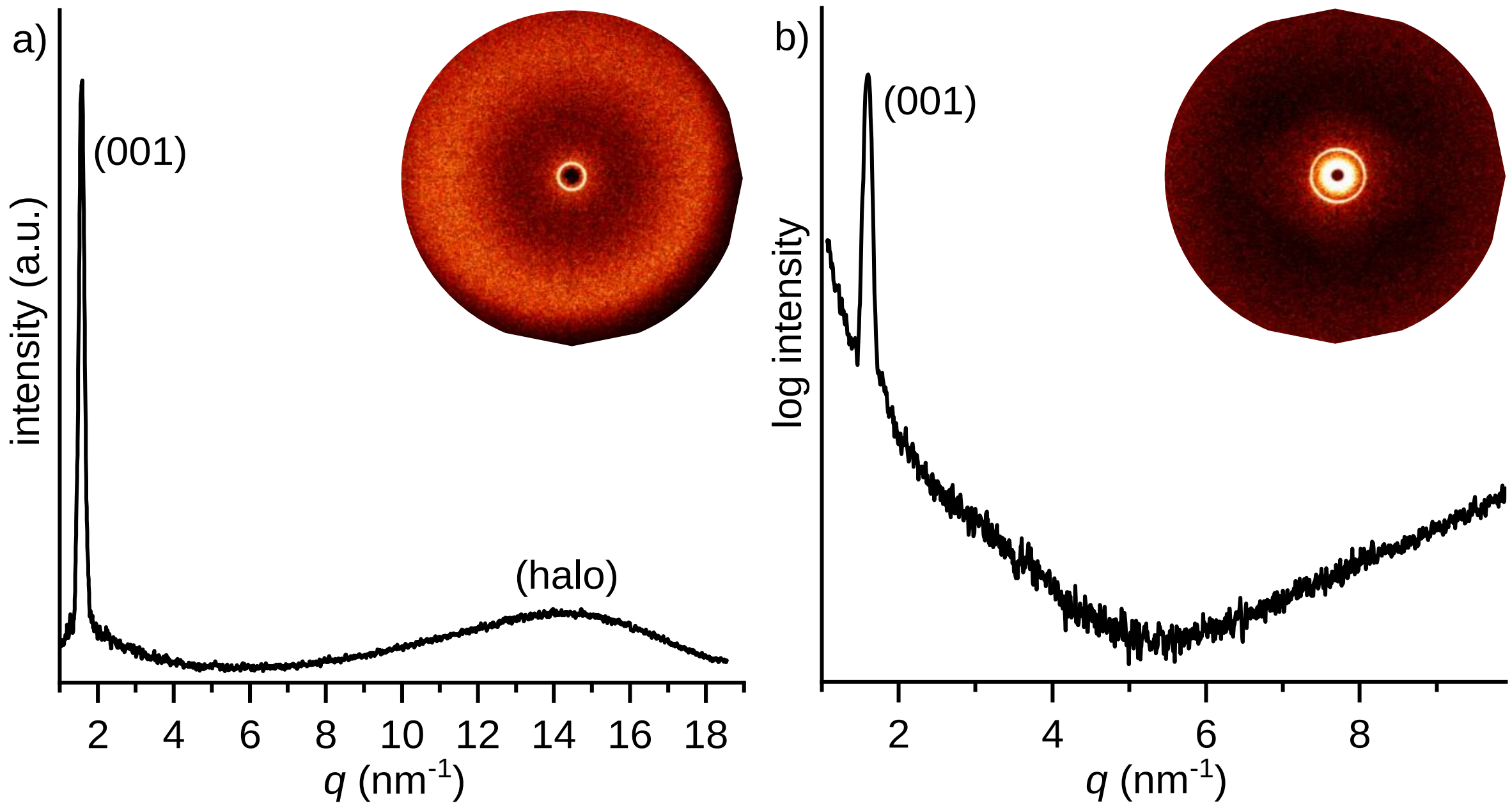

Figure S11: Diffractograms of **CN-FO-Ar** at 169 °C with diffraction patterns a) WAXS, b) SAXS.

Table S2: Detailed X-ray data (reflexes determined from SAXS except halo).

Compound	Mesophase	q / nm^{-1}	(hkl)	d_{exp} / Å	d_{calc} / Å
**H-FO-Ar**	N at 90 °C	2.56	-	24.54	-
		16.26	(halo)	3.86	-
	SmC at 50 °C	2.56	(001)	24.54	-
		5.12	(002)	12.27	12.27
		16.61	(halo)	3.78	-
**Br-FO-Ar**	SmA at 140 °C	2.24	(001)	28.05	-
		4.48	(002)	14.02	14.02
		14.48	(halo)	4.34	-
**NO_2-FO-Ar**	SmA at 169 °C	1.83	(001)	34.33	-
		15.05	(halo)	4.17	-
**CN-FO-Ar**	SmA at 169 °C	1.60	(001)	39.27	-
		13.94	(halo)	4.51	-

4. Photophysical properties

4.1. Absorption

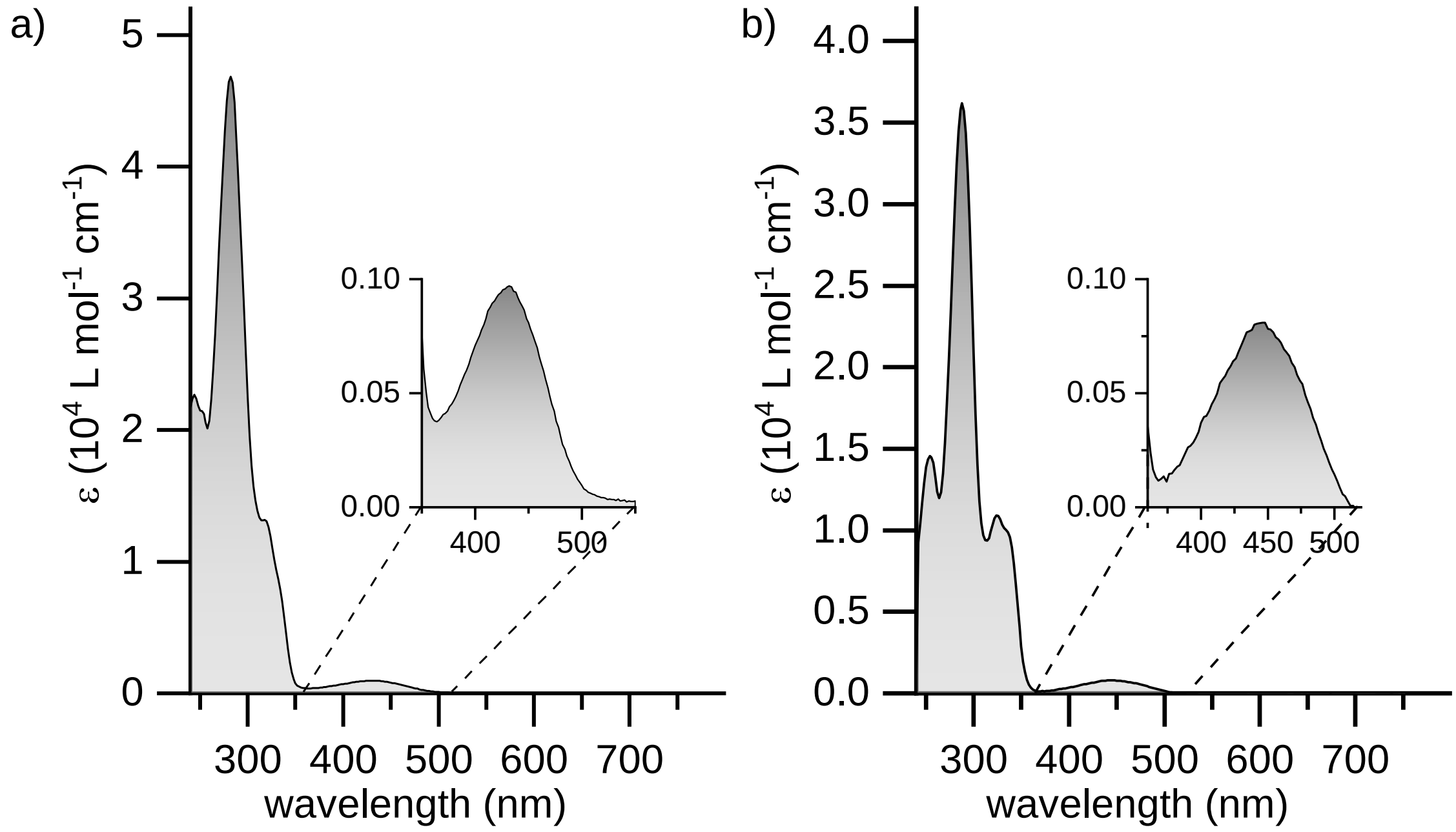

Figure S12: Absorption spectra in DCM of a) **H-FO-Ar** (c = 0.02 mM) and b) **Br-FO-Ar** (c = 0.01 mM).

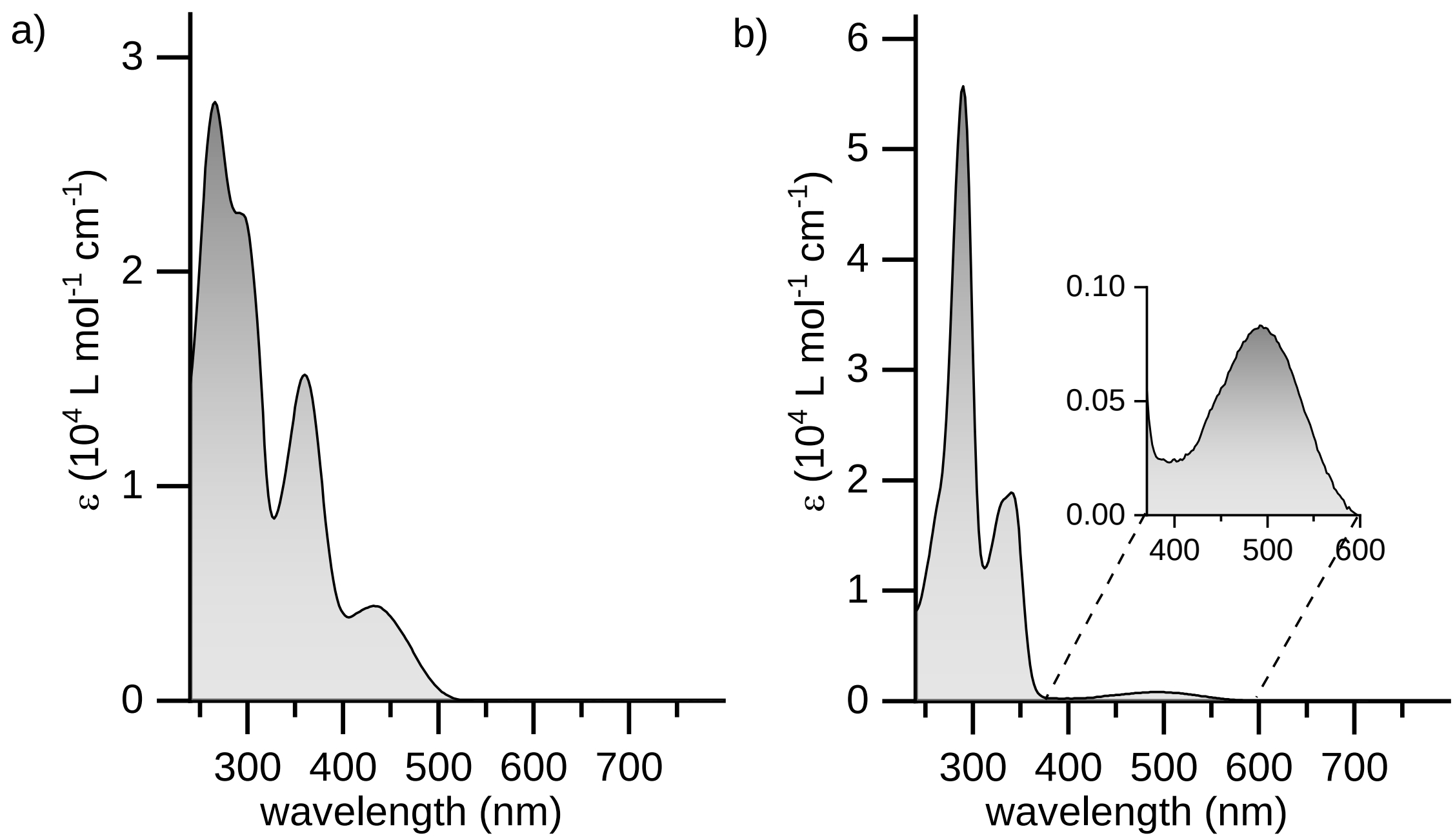

Figure S13: Absorption spectra in DCM of a) **NO_2-FO-Ar** (c = 0.02 mM) and b) **NH_2-FO-Ar** (c = 0.02 mM).

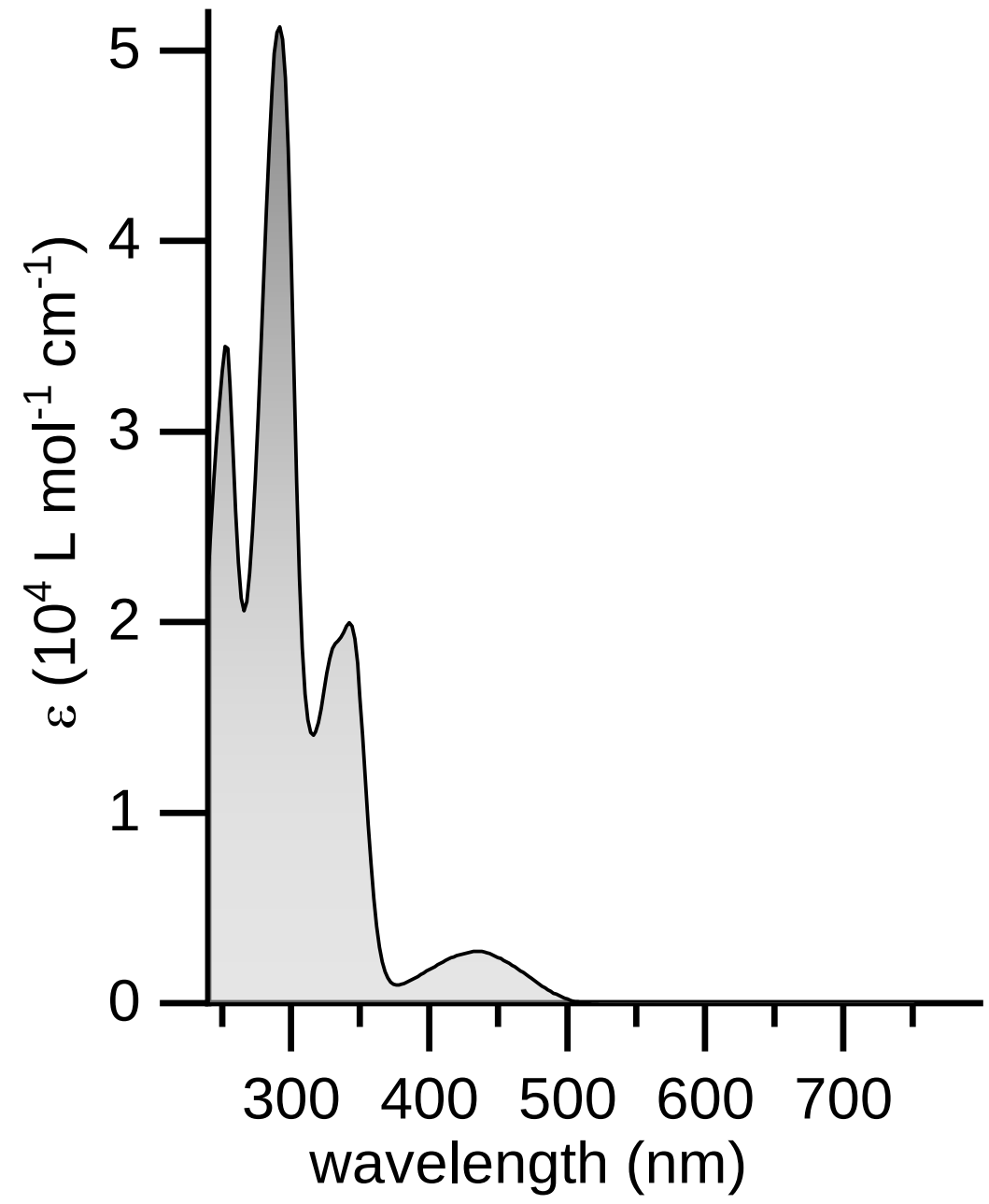

Figure S14: Absorption spectrum of **CN-FO-Ar** in DCM (c = 0.02 mM).

Table S3: Absorption maxima λ_{abs} in DCM with respecting extinction coefficients.

compound	$\lambda_{abs,1}$ / nm (ε_1 / mol^{-1}cm^{-1})	$\lambda_{abs,2}$ / nm (ε_2 / mol^{-1}cm^{-1})	$\lambda_{abs,3}$ / nm (ε_3 / mol^{-1}cm^{-1})	$\lambda_{abs,4}$ / nm (ε_4 / mol^{-1}cm^{-1})
**H-FO-Ar**	244 (23000)	282 (47000)	318* (13000)	432 (1000)
**Br-FO-Ar**	252 (15000)	286 (36000)	322 (11000)	446 (1000)
**NO_2-FO-Ar**	266 (28000)	292* (23000)	360 (15000)	432 (4000)
**NH_2-FO-Ar**	-	290 (56000)	340 (19000)	492 (1000)
**CN-FO-Ar**	252 (34000)	292 (51000)	342 (20000)	434 (3000)

*Shoulder.

Table S4: Emission maxima λ_{em} and Stokes shifts $\Delta\bar{\nu}$ in DCM.

compound	λ_{em} / nm FWHM / [nm], (cm^{-1})	$\Delta\bar{\nu}$ nm (cm^{-1})
**H-FO-Ar**	588 [141] (3928)	156 (6144)
**Br-FO-Ar**	597 [147] (3851)	151 (5670)
**NO_2-FO-Ar**	618 [141] (3630)	186 (6974)
**NH_2-FO-Ar**	-	-
**CN-FO-Ar**	604 [143] (3752)	170 (6483)

4.2. Emission in solid state and solution

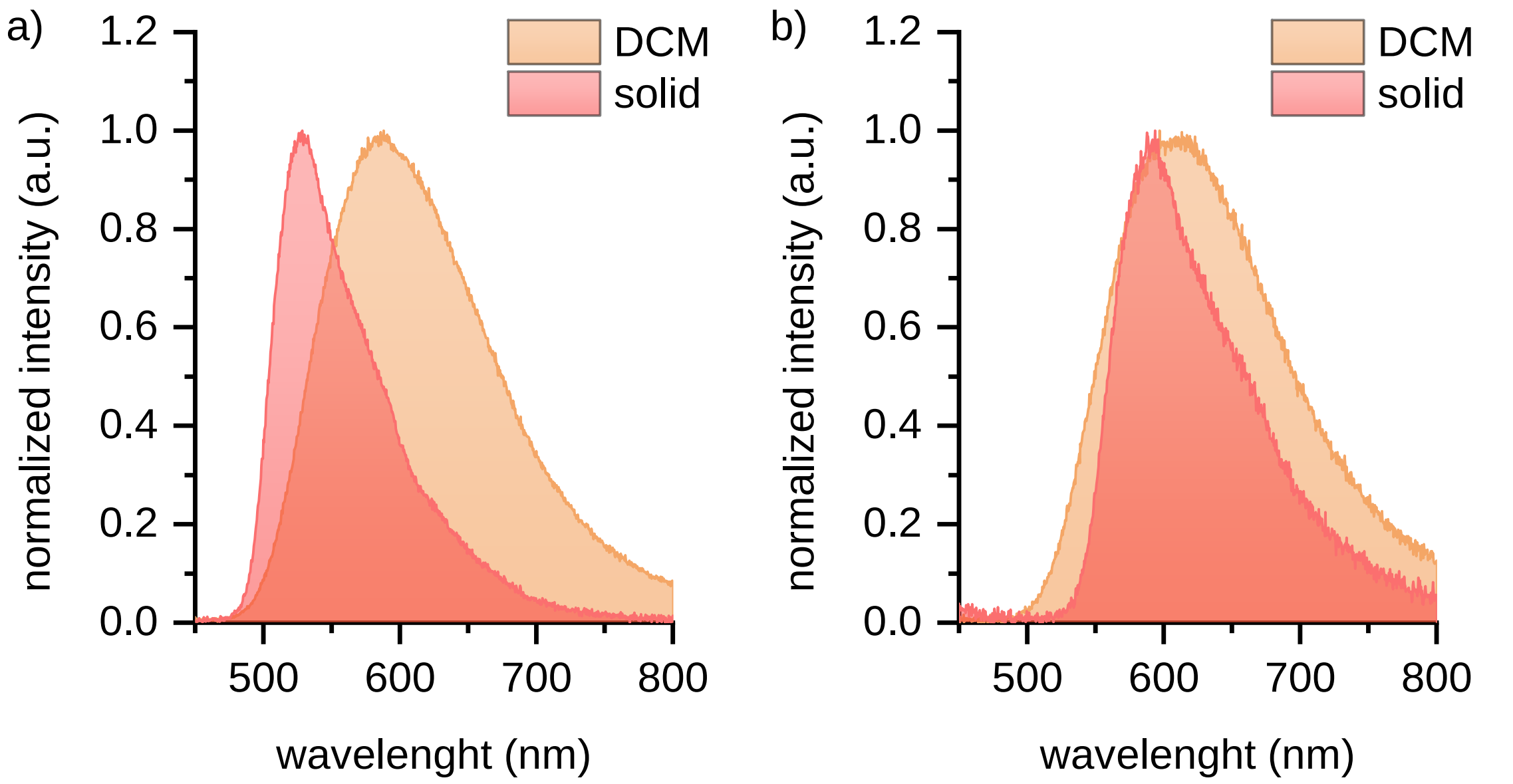

Figure S15: Emission spectra in solid state and DCM of a) **H-FO-Ar** ($\lambda_{exc,solid}$ = 365 nm, $\lambda_{exc,DCM}$ = 400 nm, c = 0.02 mM) and b) **Br-FO-Ar** ($\lambda_{exc,solid}$ = 370 nm, $\lambda_{exc,DCM}$ = 400 nm, c = 0.01 mM).

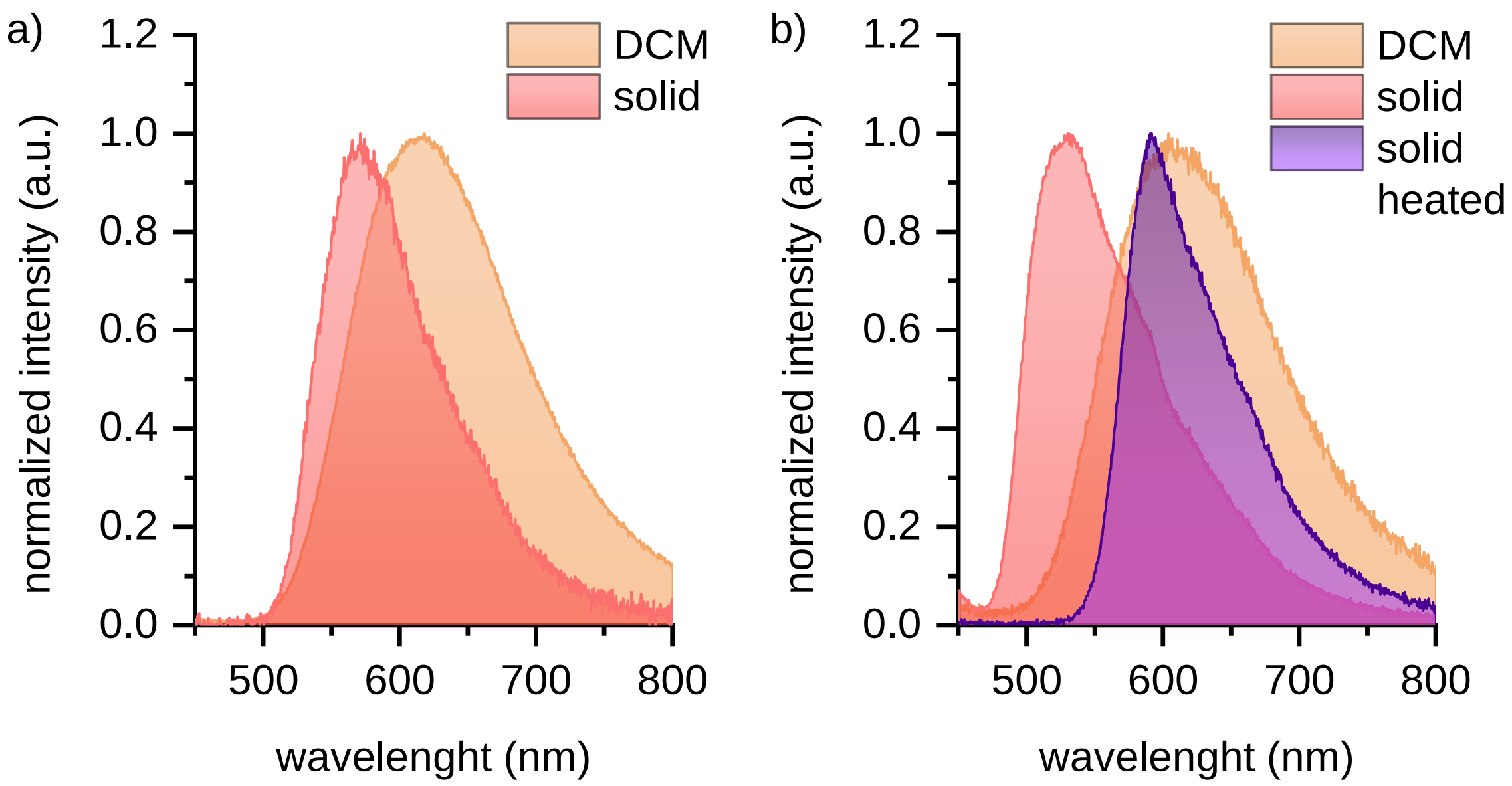

Figure S16: Emission spectra of a) **NO_2-FO-Ar** in solid state and DCM ($\lambda_{exc,solid}$ = 370 nm, $\lambda_{exc,DCM}$ = 360 nm, c = 0.02 mM) and b) **CN-FO-Ar** in solid state, in solid state after heating and in DCM ($\lambda_{exc,solid}$ = 370 nm, $\lambda_{exc,solidheated}$ = 370 nm, $\lambda_{exc,DCM}$ = 280 nm, c = 0.01 mM).

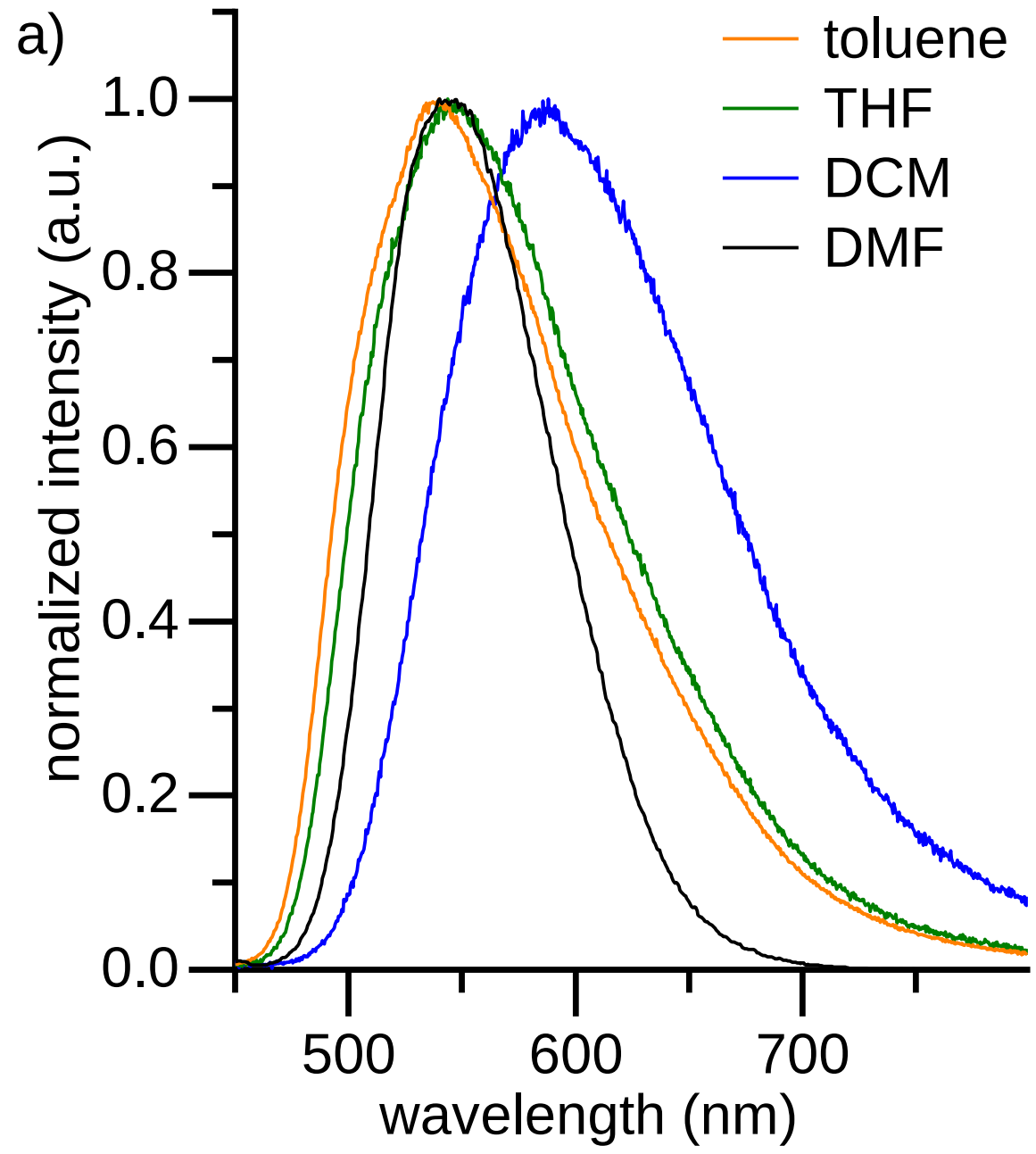

Figure S17: Emission spectra in DCM, THF and toluene of a) **H-FO-Ar** ($\lambda_{exc,DMF}$ = 400 nm, $\lambda_{exc,DCM}$ = 400 nm, $\lambda_{exc,THF}$ = 400 nm, $\lambda_{exc,Toluene}$ = 375 nm) and b) **Br-FO-Ar** ($\lambda_{exc,DMF}$ = 400 nm, $\lambda_{exc,DCM}$ = 400 nm, $\lambda_{exc,THF}$ = 400 nm, $\lambda_{exc,Toluene}$ = 350 nm).

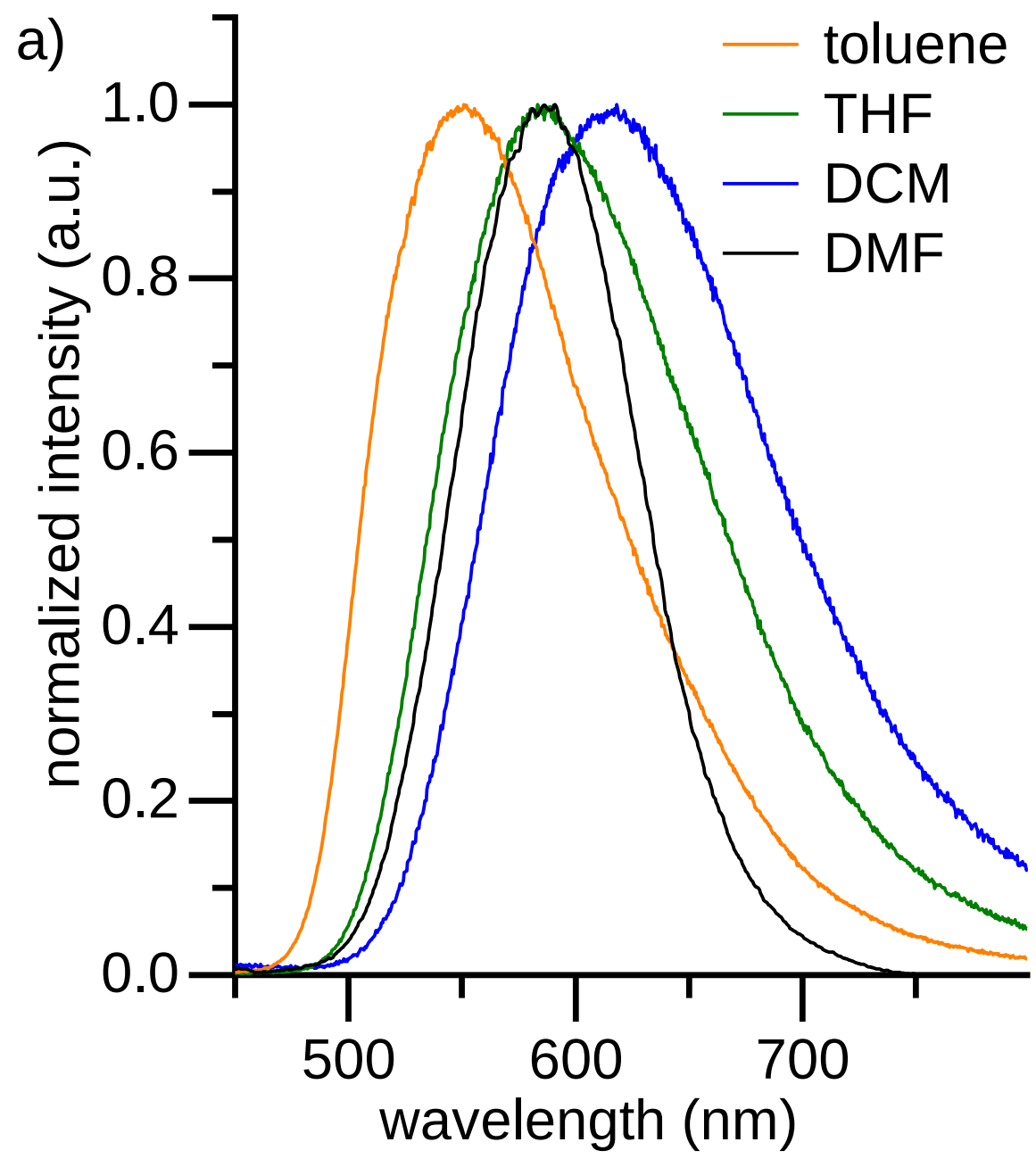

Figure S18: Emission spectra in DCM, THF and toluene of a) **NO_2-FO-Ar** ($\lambda_{exc,DMF}$ = 400 nm, $\lambda_{exc,DCM}$ = 360 nm, $\lambda_{exc,THF}$ = 400 nm, $\lambda_{exc,Toluene}$ = 380 nm) and b) **CN-FO-Ar** ($\lambda_{exc,DMF}$ = 400 nm, $\lambda_{exc,DCM}$ = 280 nm, $\lambda_{exc,THF}$ = 400 nm, $\lambda_{exc,Toluene}$ = 340 nm).

Table S5: Detailed emission properties. Emission maxima λ_{em} with respective full width at half maximum (FWHM), absolute quantum yields Φ, fluorescence lifetimes τ, χ^2 values and rate constants for radiative and nonradiative deactivation k_r and k_{nr} in solution and solid state.

	solvent	$E_T(30)$[b]	**H-FO-Ar**	**Br-FO-Ar**	**NO_2-FO-Ar**	**CN-FO-Ar**
λ_{em} / nm FWHM/ [nm] (cm^{-1})	solid	-	528 [81] (2730)	594 [103] (2771)	571 [96] (2837)	530 [102] (3453)
	toluene	33.9	540 [121] (4002)	556 [124] (3862)	551 [119] (3767)	554 [124] (3929)
	THF	37.4	544 [123] (3966)	575 [130] (3871)	583 [133] (3731)	578 [132] (3826)
	DCM	40.7	588 [141] (3928)	597 [147] (3851)	618 [141] (3630)	604 [143] (3752)
	DMF	43.2	540 [88] (2896)	563 [90] (2797)	591 [92] (2677)	572 [94] (2852)
	solid^h	-	-	-	-	591 [87] (2336)
Φ / % [τ / ns] (χ^2)	solid[a]	-	35 [6.1] (0.99)	16 [3.6] (1.11)	2 [7.4] (1.09)	10 [5.9] (1.38)
	toluene	33.9	14 [19.2] (1.17)	7 [2.8] (1.15)	6 [7.7] (1.09)	20 [10.7] (1.00)
	THF	37.4	15	6	27	17
	DCM	40.7	2	1	4	6
	solid^h	-	-	-	-	13 [7.8] (1.06)
k_r | k_{nr} / 10^7 s^{-1}	toluene	33.9	5.73 | 10.64	4.44 | 23.33	0.27 | 13.20	1.68 | 15.15
	solid	-	0.73 | 4.47	2.50 | 33.15	0.78 | 12.24	1.86 | 7.45
	solid^h	-	-	-	-	1.67 | 11.14

[a] emission decays were fitted with two components for **Br-FO-Ar**, **NO_2-FO-Ar**, **CN-FO-Ar**. Average lifetimes τ_{av} were calculated as $\tau_{av} = (a_1\tau_1^2+a_2\tau_2^2)/(a_1\tau_1+a_2\tau_2)$, [b] $E_T(30)$ values in kcal mol^{-1} taken from the literature.[9]

Table S6: Absolute quantum yields in solid state, toluene, THF and DCM.

compound	**H-FO-Ar**	**Br-FO-Ar**	**NO_2-FO-Ar**	**CN-FO-Ar**
solvent	Φ / %	Φ / %	Φ / %	Φ / %
solid	35	16	2	10
toluene	14	7	6	20
THF	15	6	27	17
DCM	2	1	4	6
solid[h]	-	-	-	13

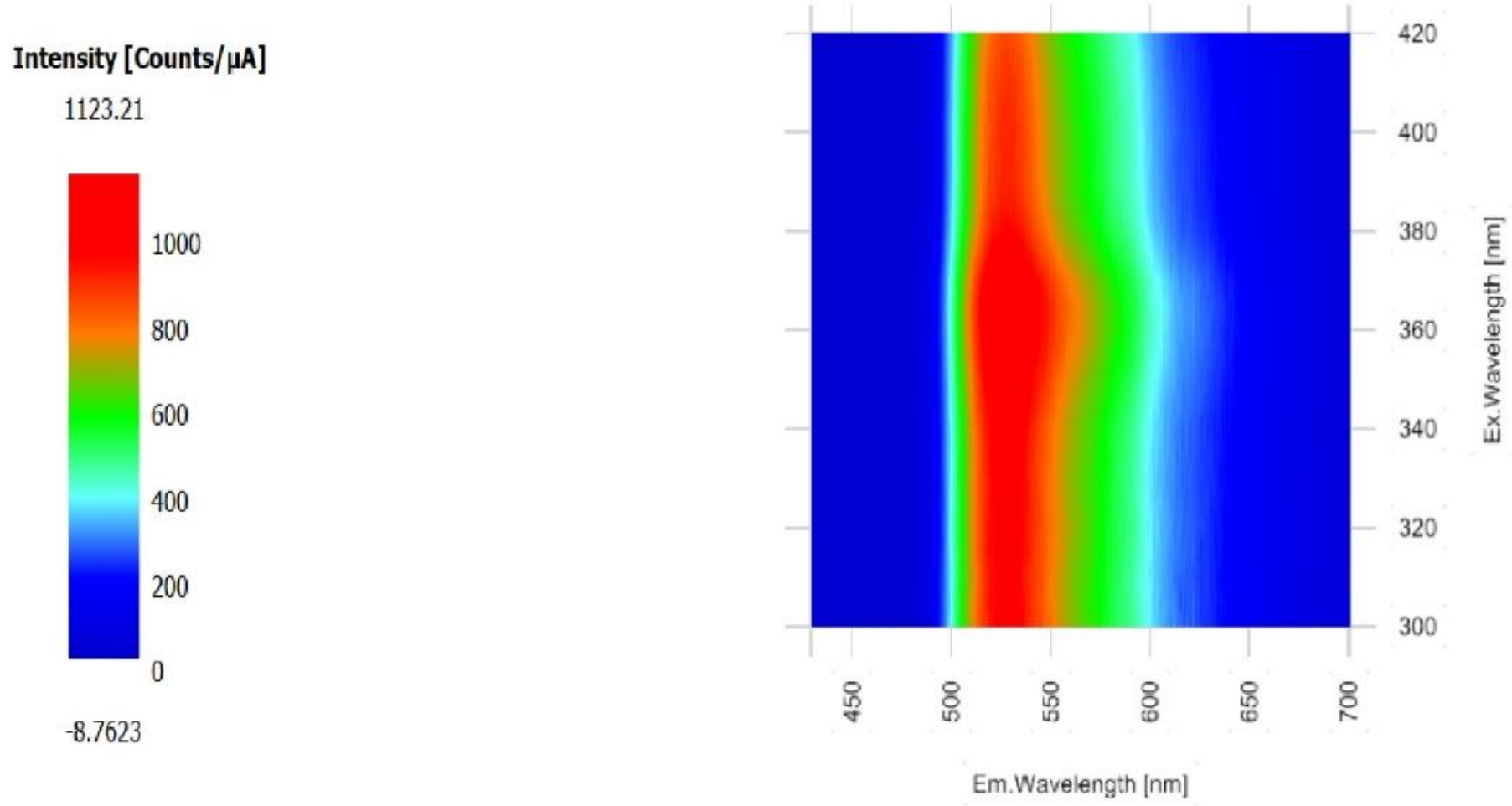

Figure S19: Excitation-emission map **H-FO-Ar** (solid).

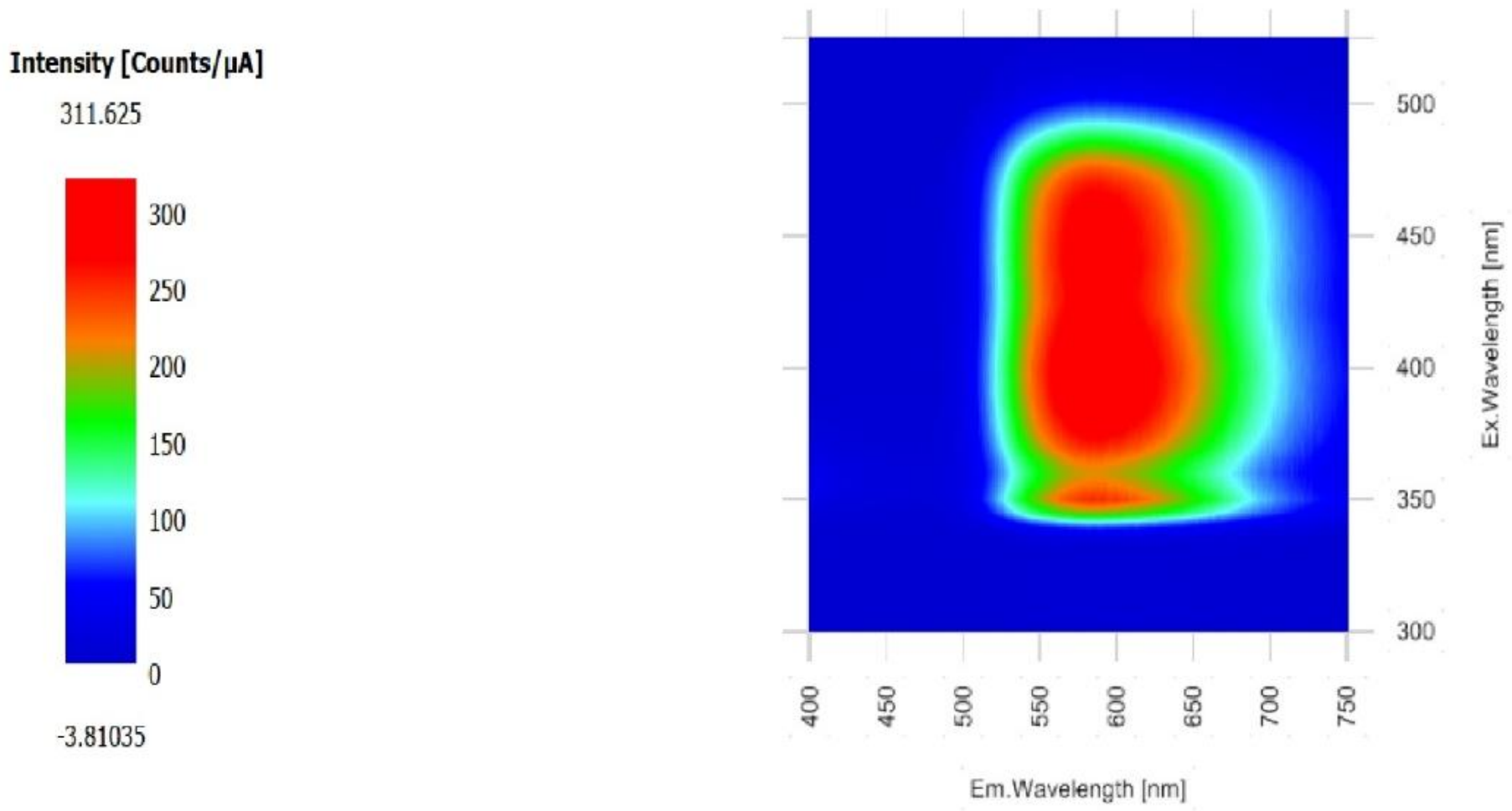

Figure S20: Excitation-emission map **H-FO-Ar** (DCM).

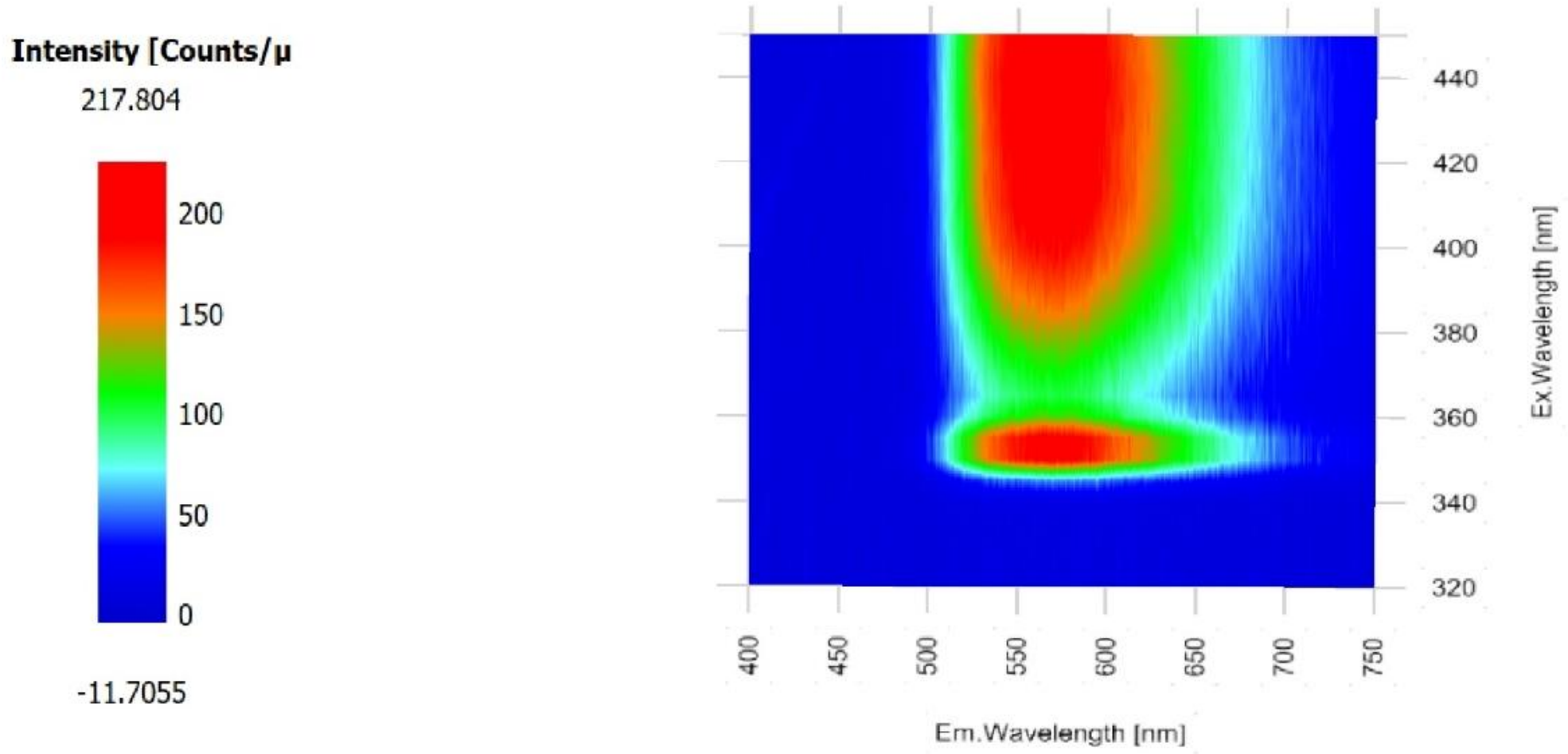

Figure S21: Excitation-emission map **H-FO-Ar** (THF).

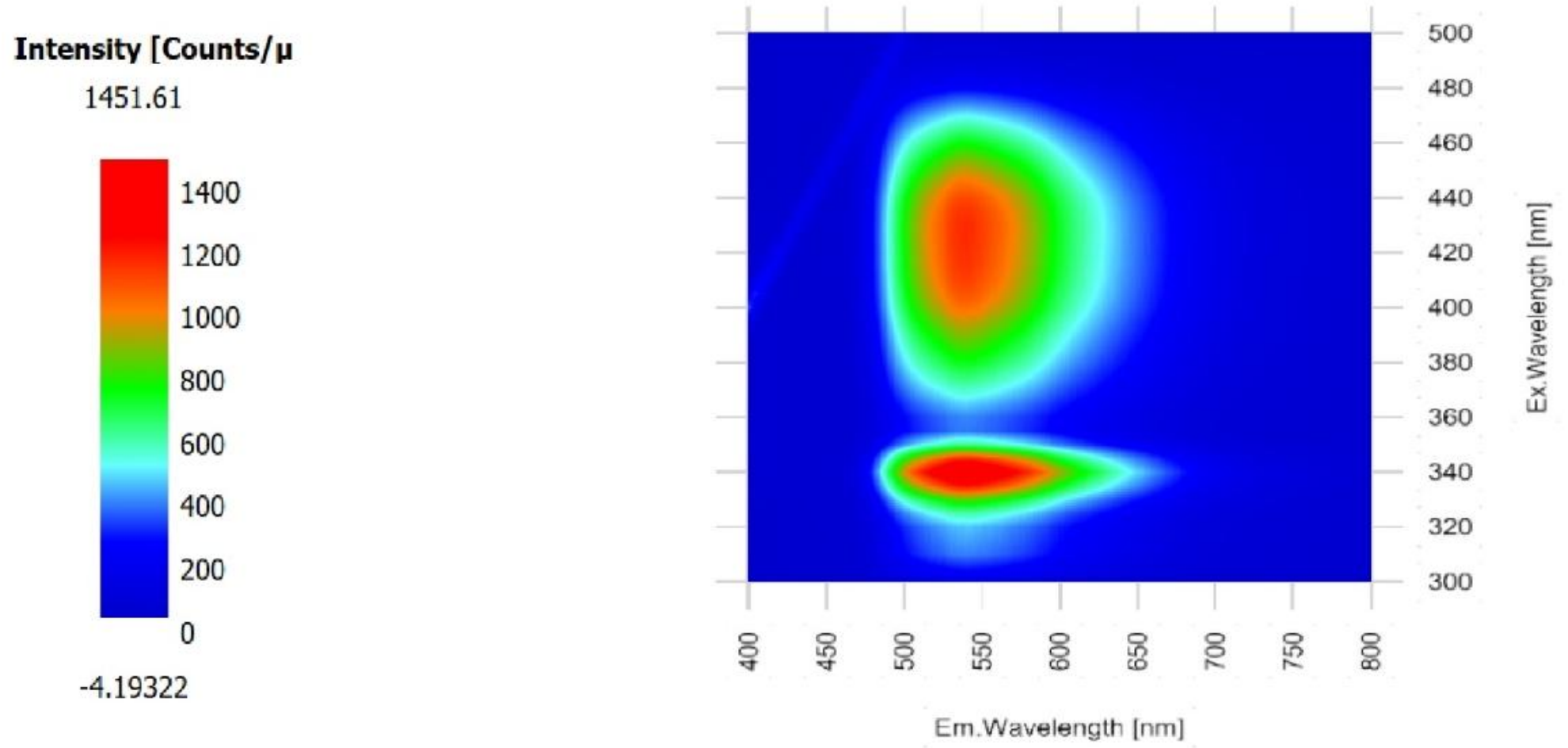

Figure S22: Excitation-emission map **H-FO-Ar** (toluene).

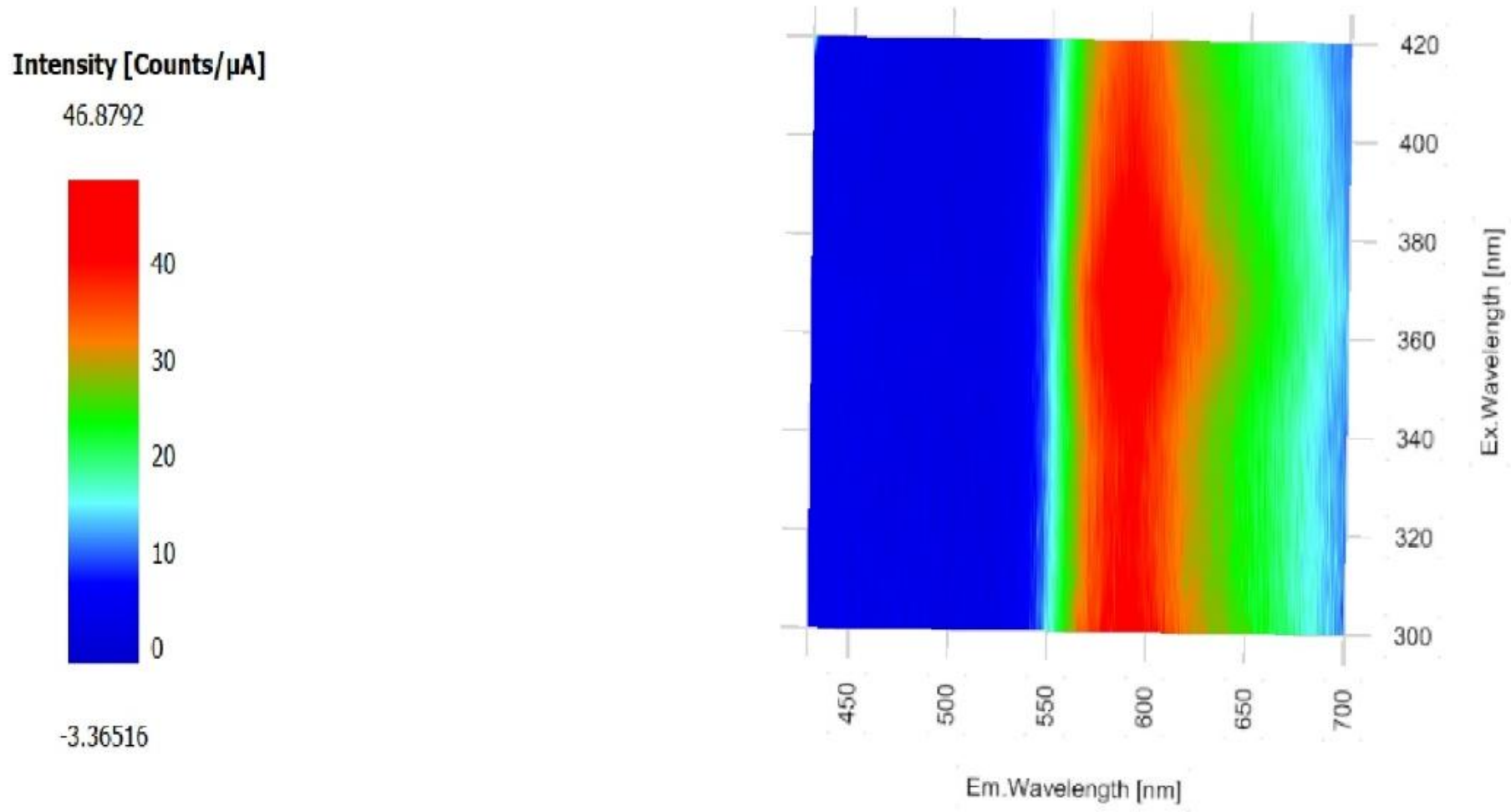

Figure S23: Excitation-emission map **Br-FO-Ar** (solid).

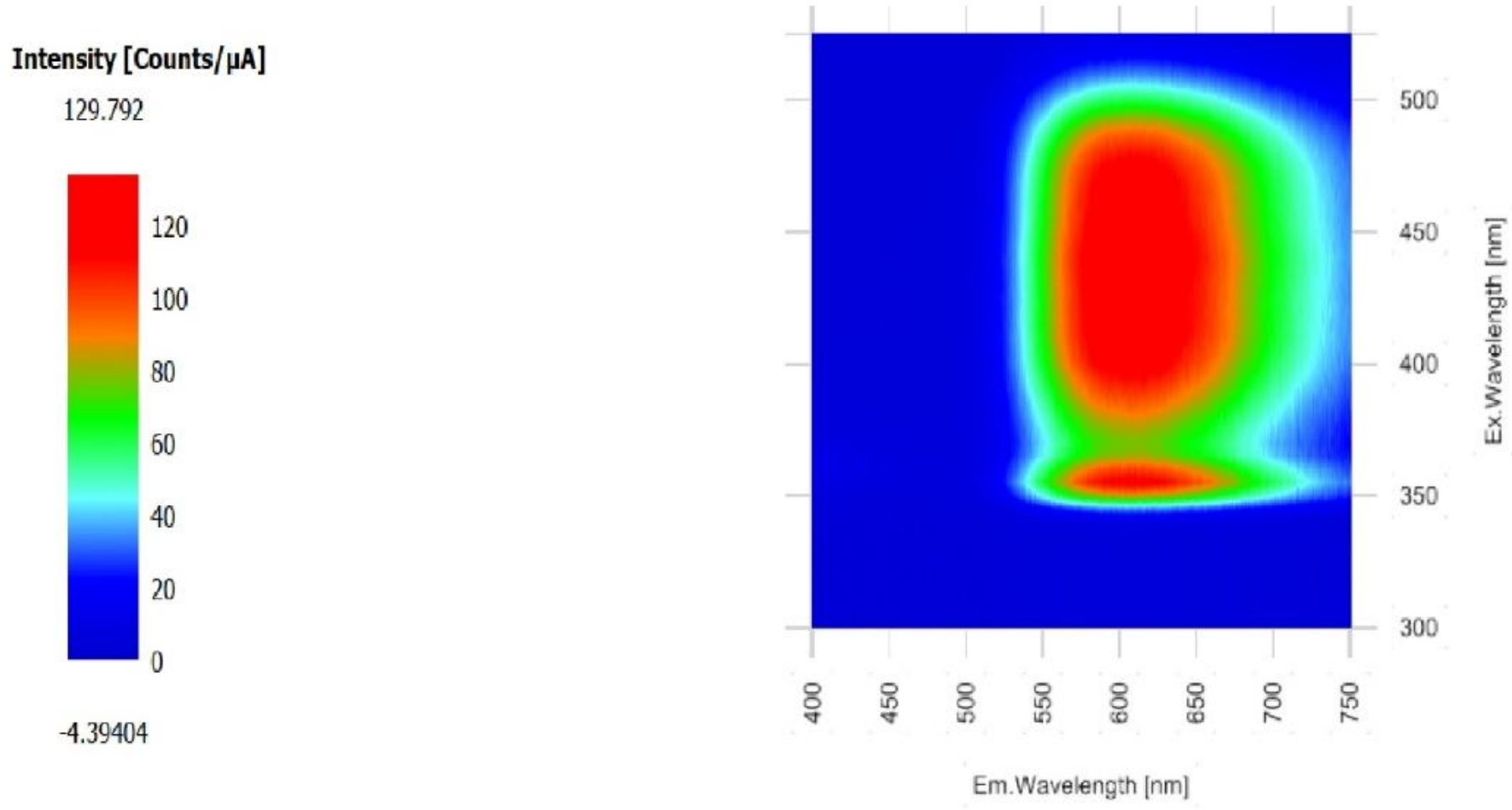

Figure S24: Excitation-emission map **Br-FO-Ar** (DCM).

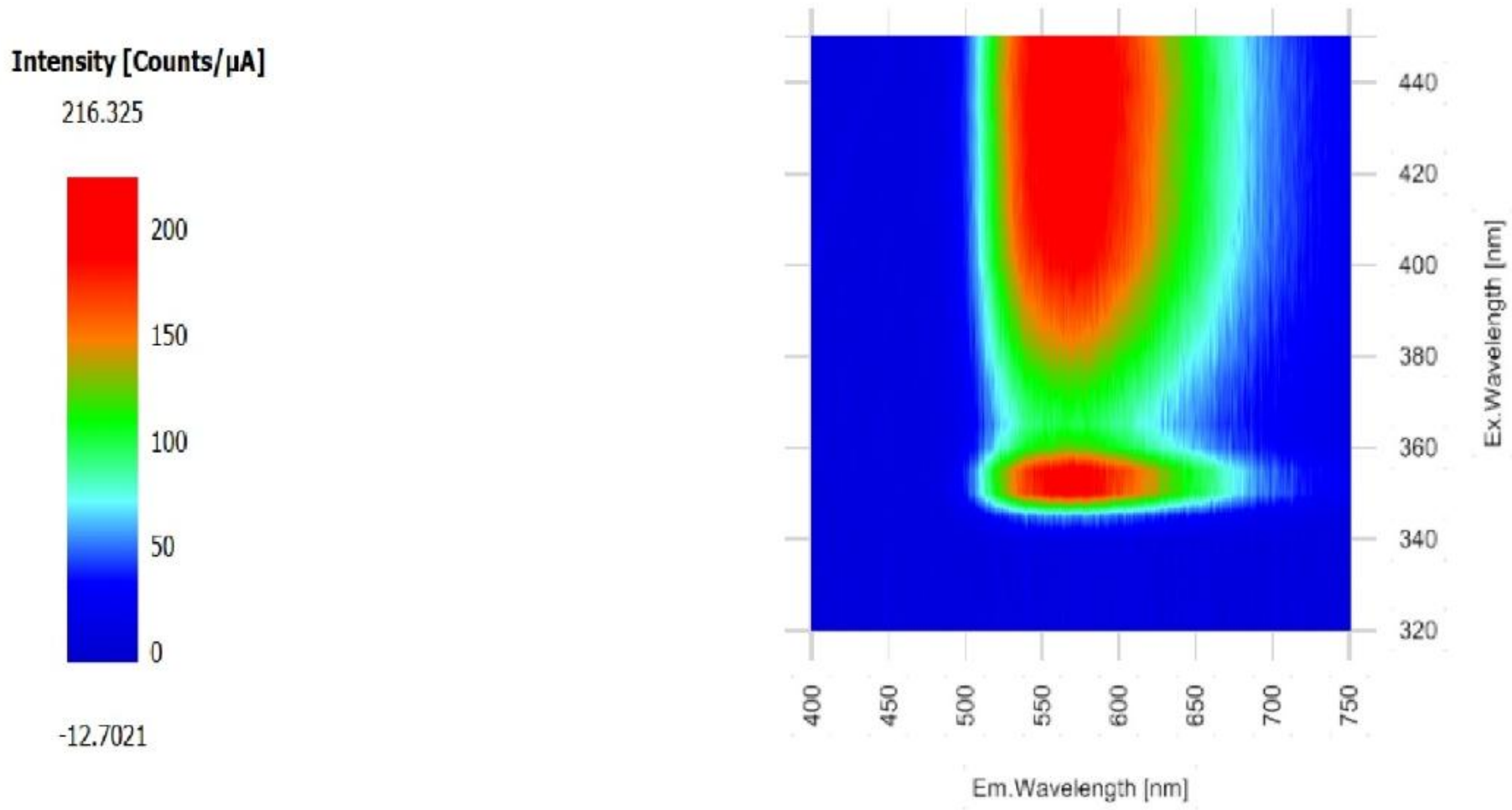

Figure S25: Excitation-emission map **Br-FO-Ar** (THF).

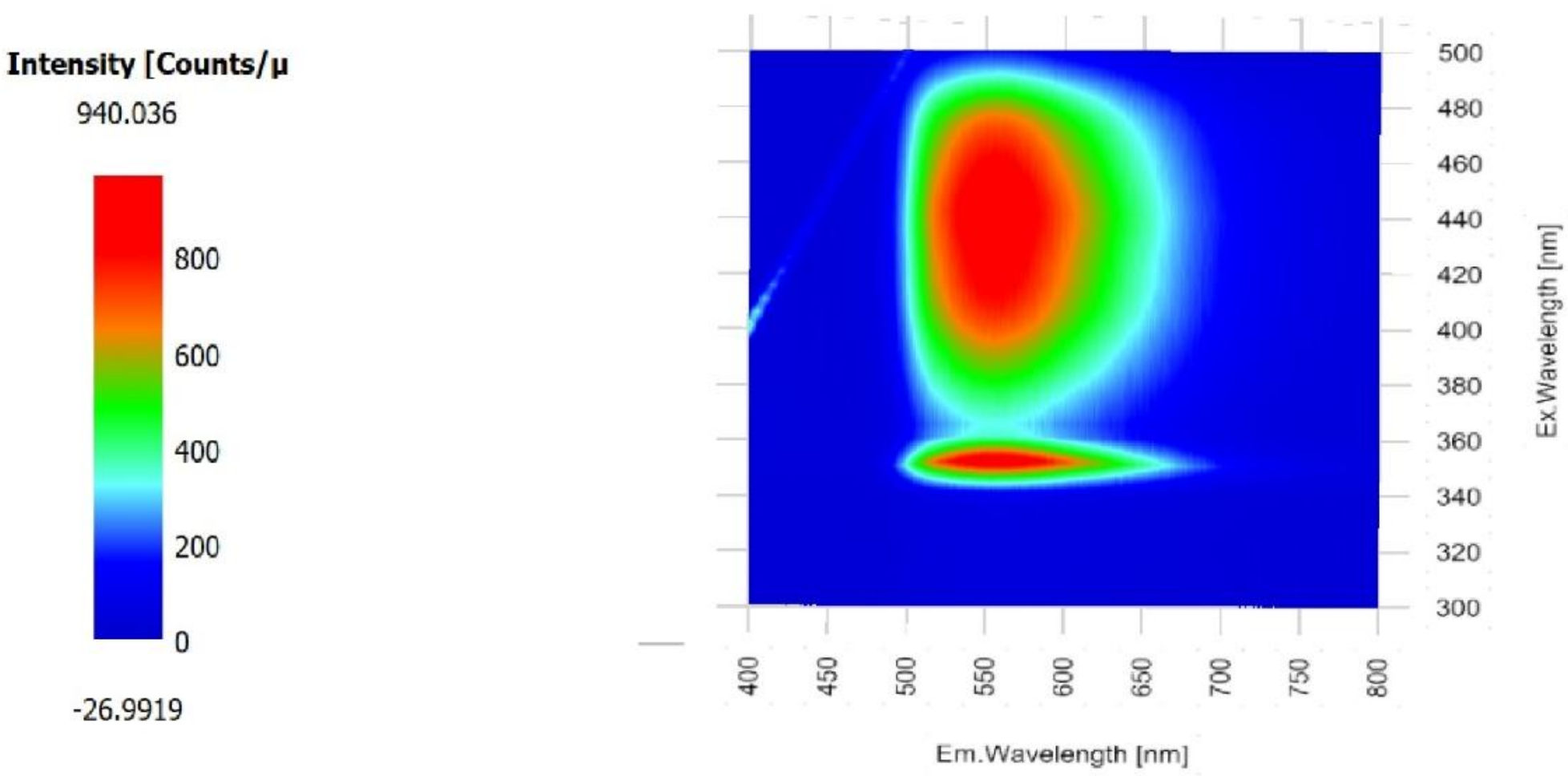

Figure S26: Excitation-emission map **Br-FO-Ar** (toluene).

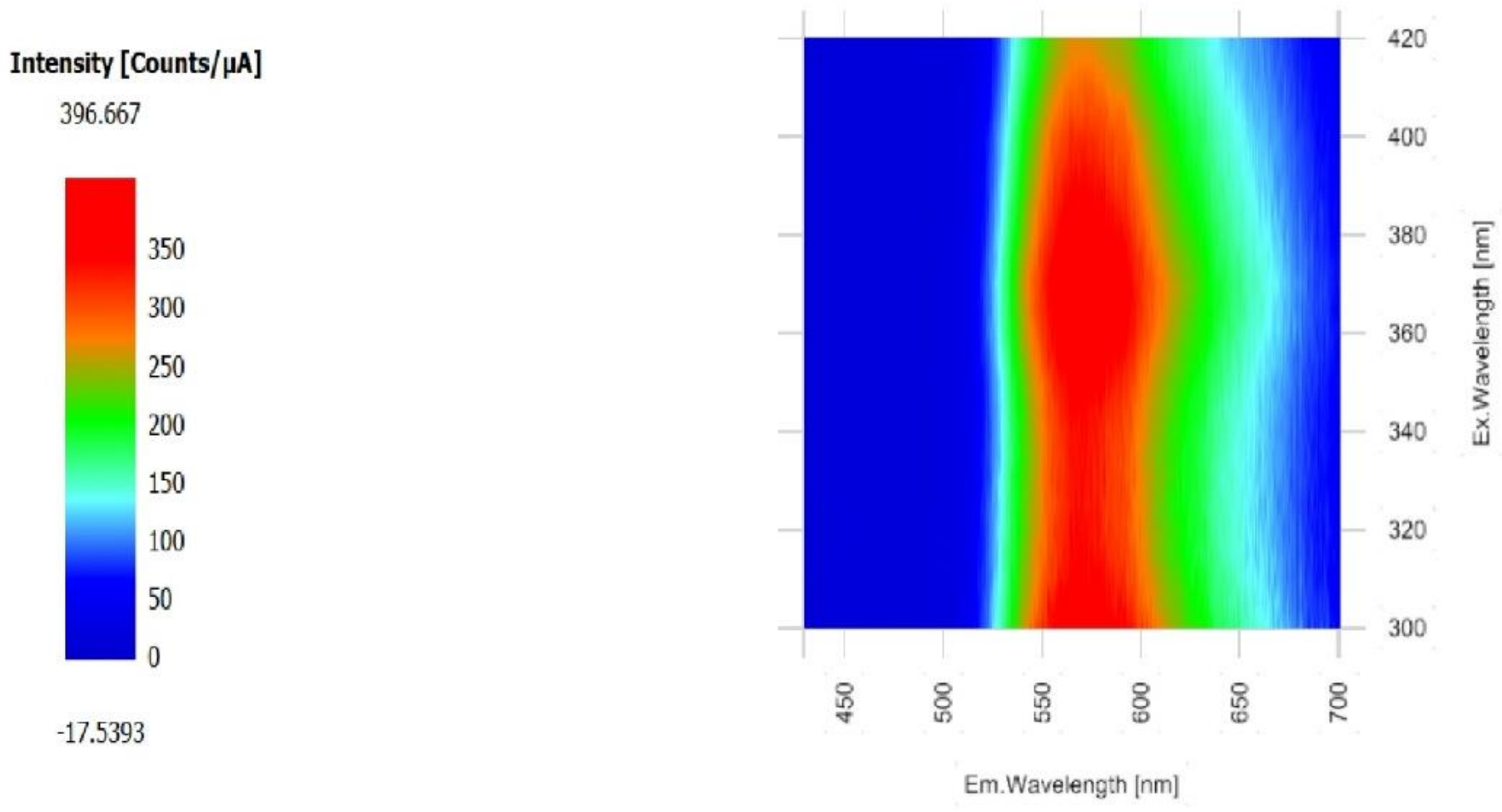

Figure S27: Excitation-emission map **NO_2-FO-Ar** (solid).

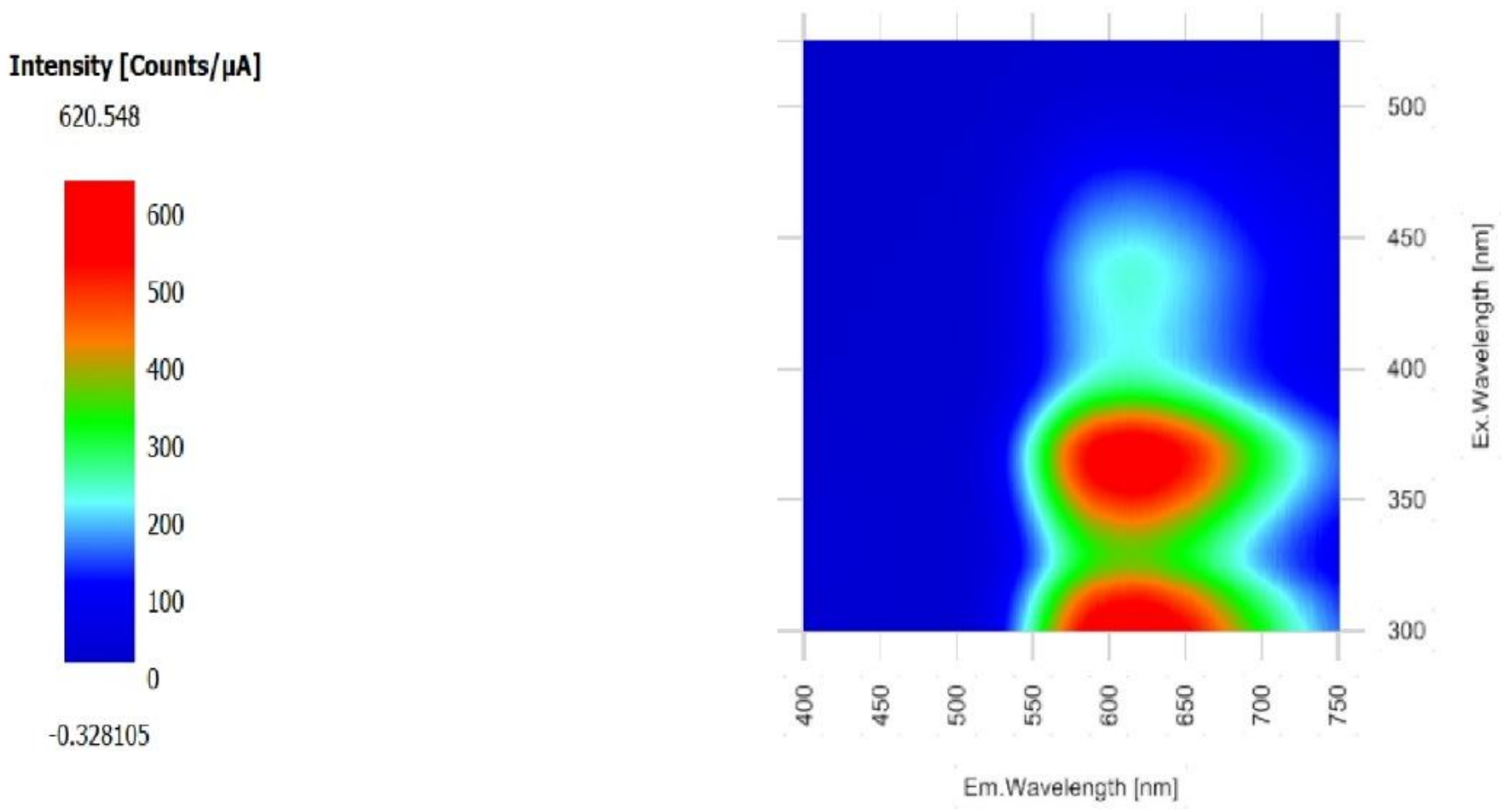

Figure S28: Excitation-emission map **NO_2-FO-Ar** (DCM).

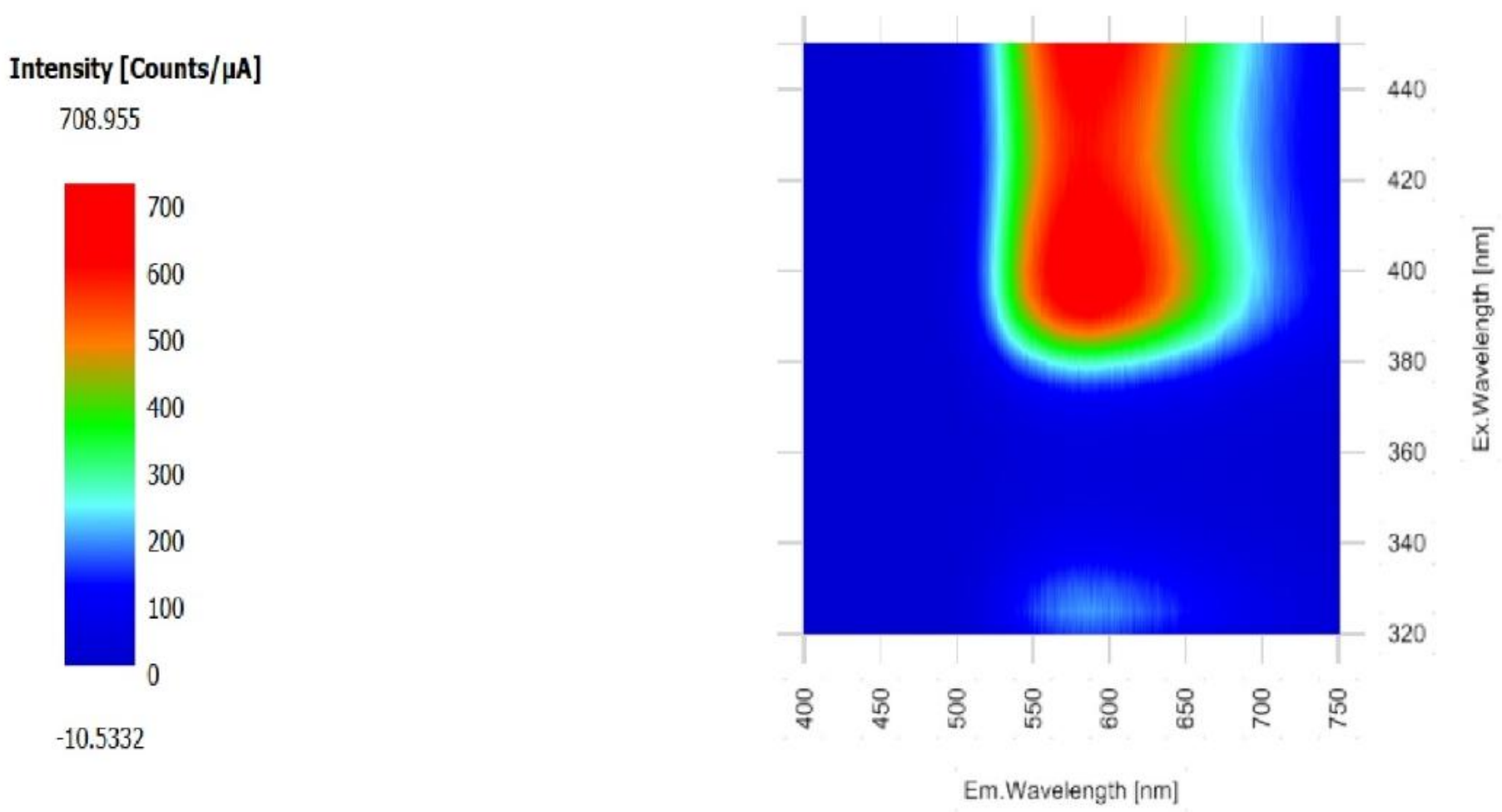

Figure S29: Excitation-emission map **NO_2-FO-Ar** (THF).

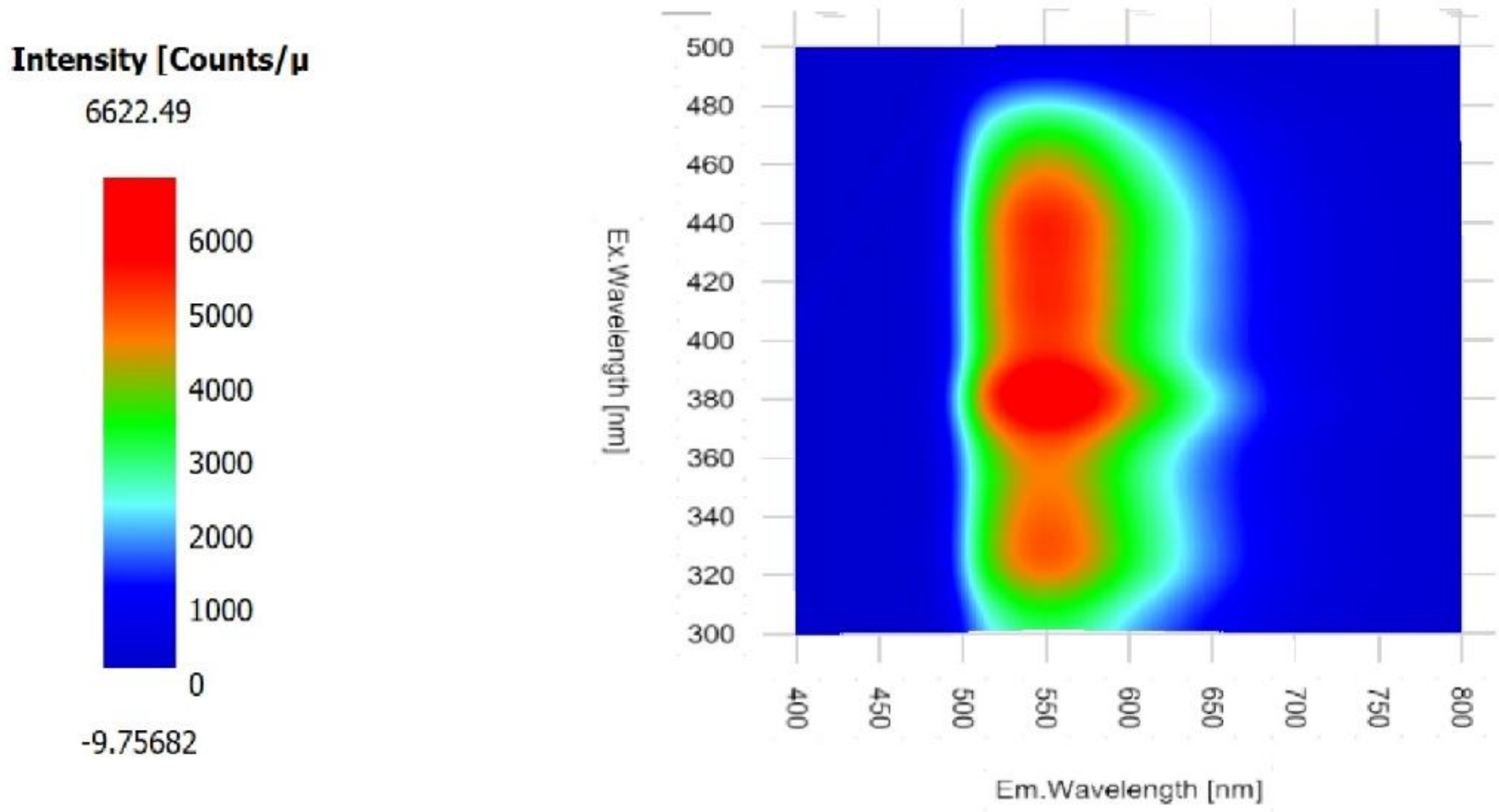

Figure S30: Excitation-emission map **NO_2-FO-Ar** (toluene).

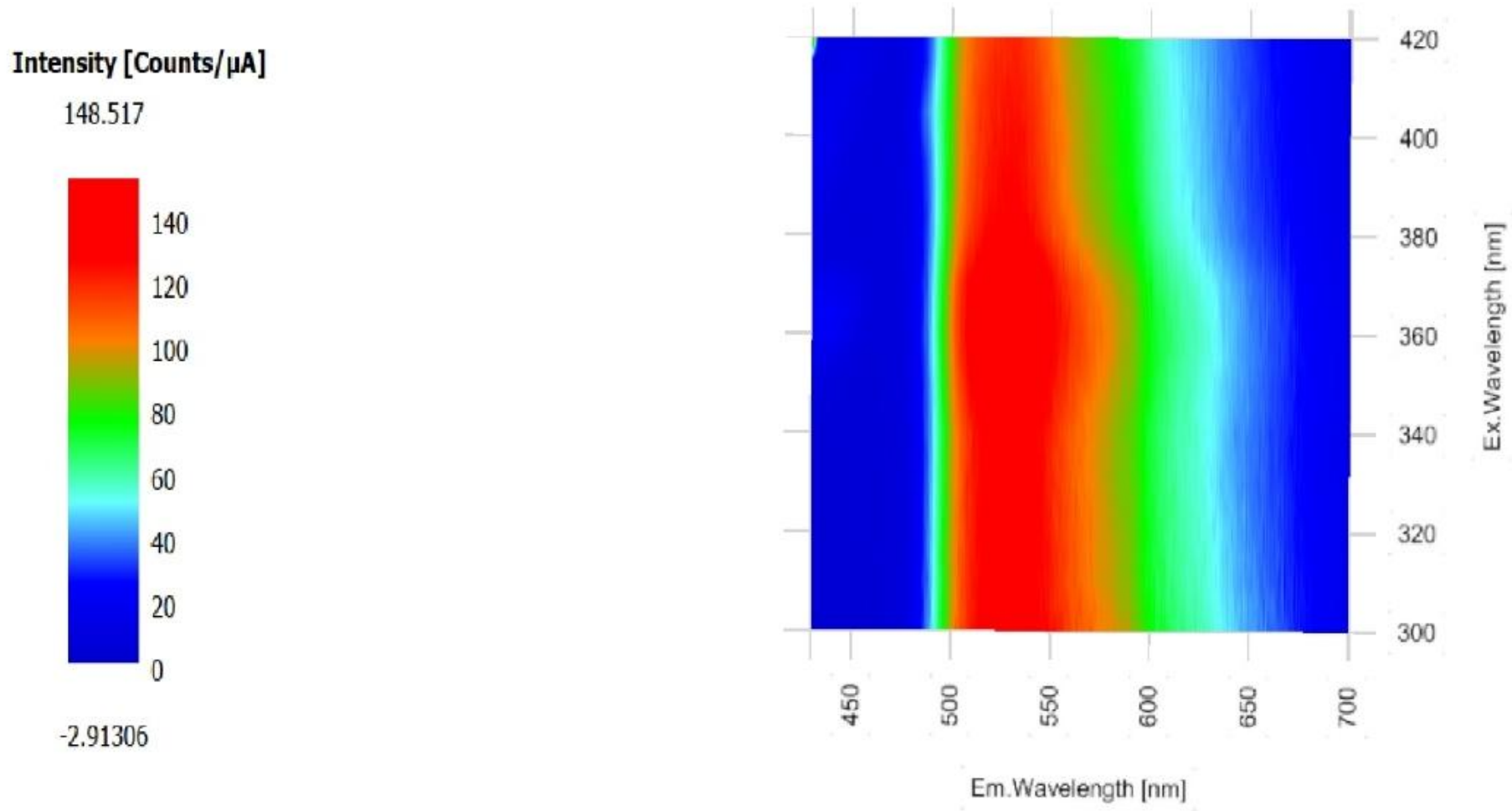

Figure S31: Excitation-emission map **CN-FO-Ar** (solid, nonheated).

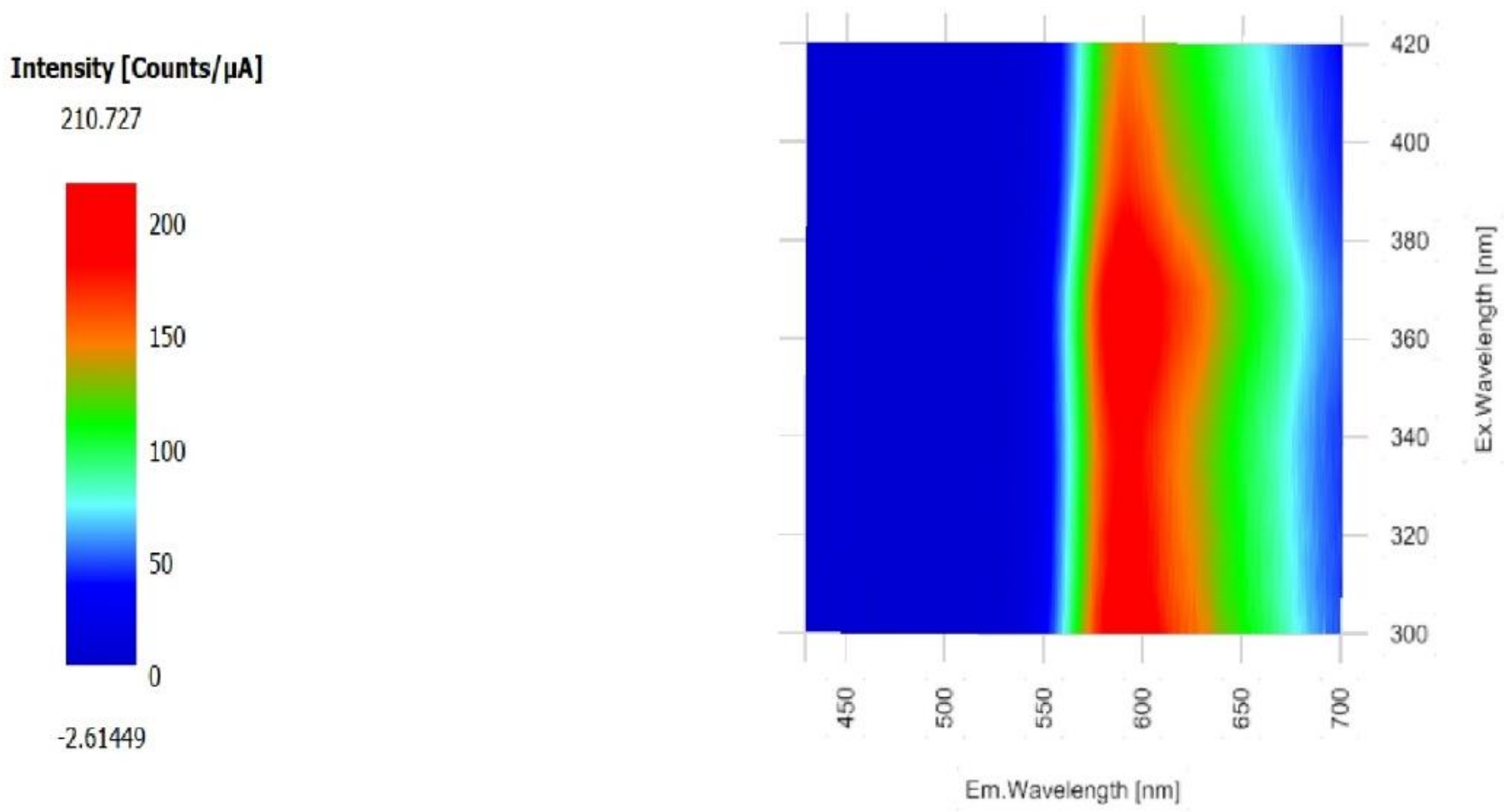

Figure S32: Excitation-emission map **CN-FO-Ar** (solid, heated).

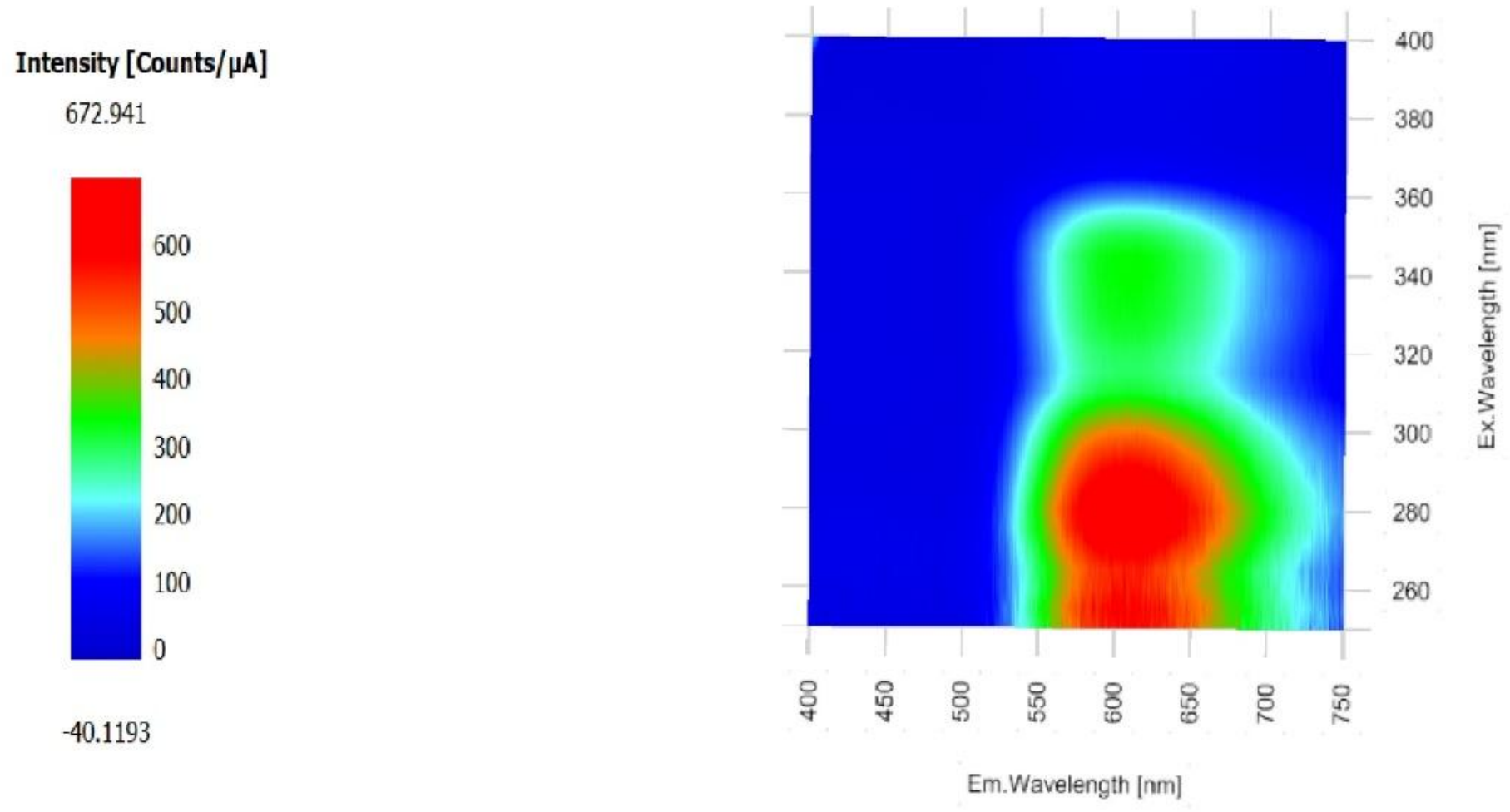

Figure S33: Excitation-emission map **CN-FO-Ar** (DCM).

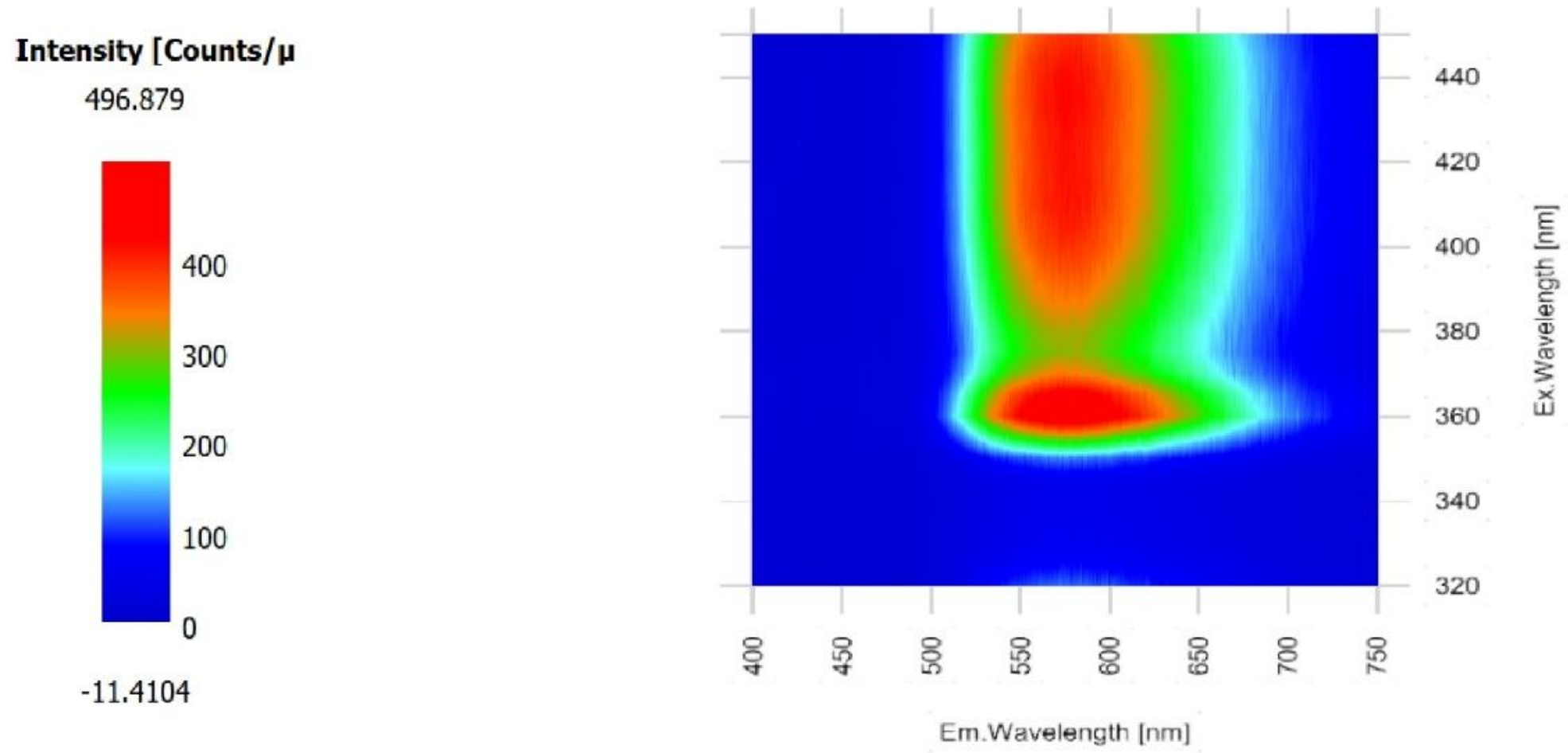

Figure S34: Excitation-emission map **CN-FO-Ar** (THF).

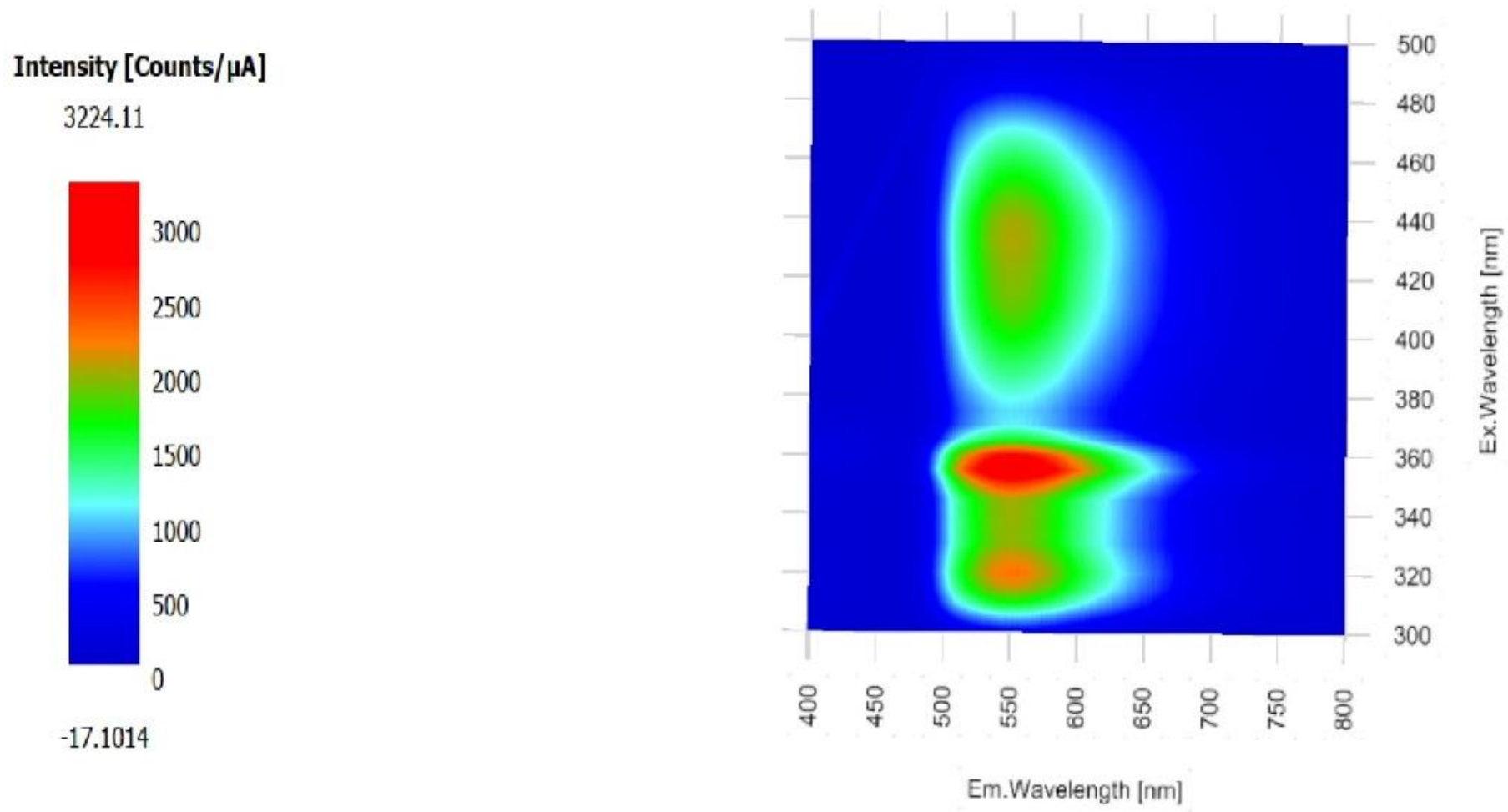

Figure S35: Excitation-emission map **CN-FO-Ar** (toluene).

4.3. Fluorescence lifetime

Table S7: Quantum yields Φ, lifetimes τ, χ^2 values and rate constants for radiative and nonradiative deactivation k_r and k_{nr} in toluene or in solid state.

compound	solvent	Φ in %	τ / ns	χ^2	k_r / 10^7 s^{-1}	k_{nr} / 10^7 s^{-1}
**H-FO-Ar**	toluene	35	6.11	0.99	5.73	10.64
	solid	14	19.22	1.17	0.73	4.47
**Br-FO-Ar**	toluene	16	3.60	1.11	4.44	23.33
	solid[a]	7	2.81	1.15	2.50	33.15
**NO_2-FO-Ar**	toluene	2	7.42	1.09	0.27	13.20
	solid[a]	6	7.68	1.09	0.78	12.24
**CN-FO-Ar**	toluene	10	5.94	1.38	1.68	15.15
	solid[a]	20	10.73	1.00	1.86	7.45
	solid[h]	13	7.81	1.06	1.67	11.14

[a] emission decays were fitted with two components. Average lifetimes τ_{av} were calculated as $\tau_{av} = (a_1\tau_1^2+a_2\tau_2^2)/(a_1\tau_1+a_2\tau_2)$.

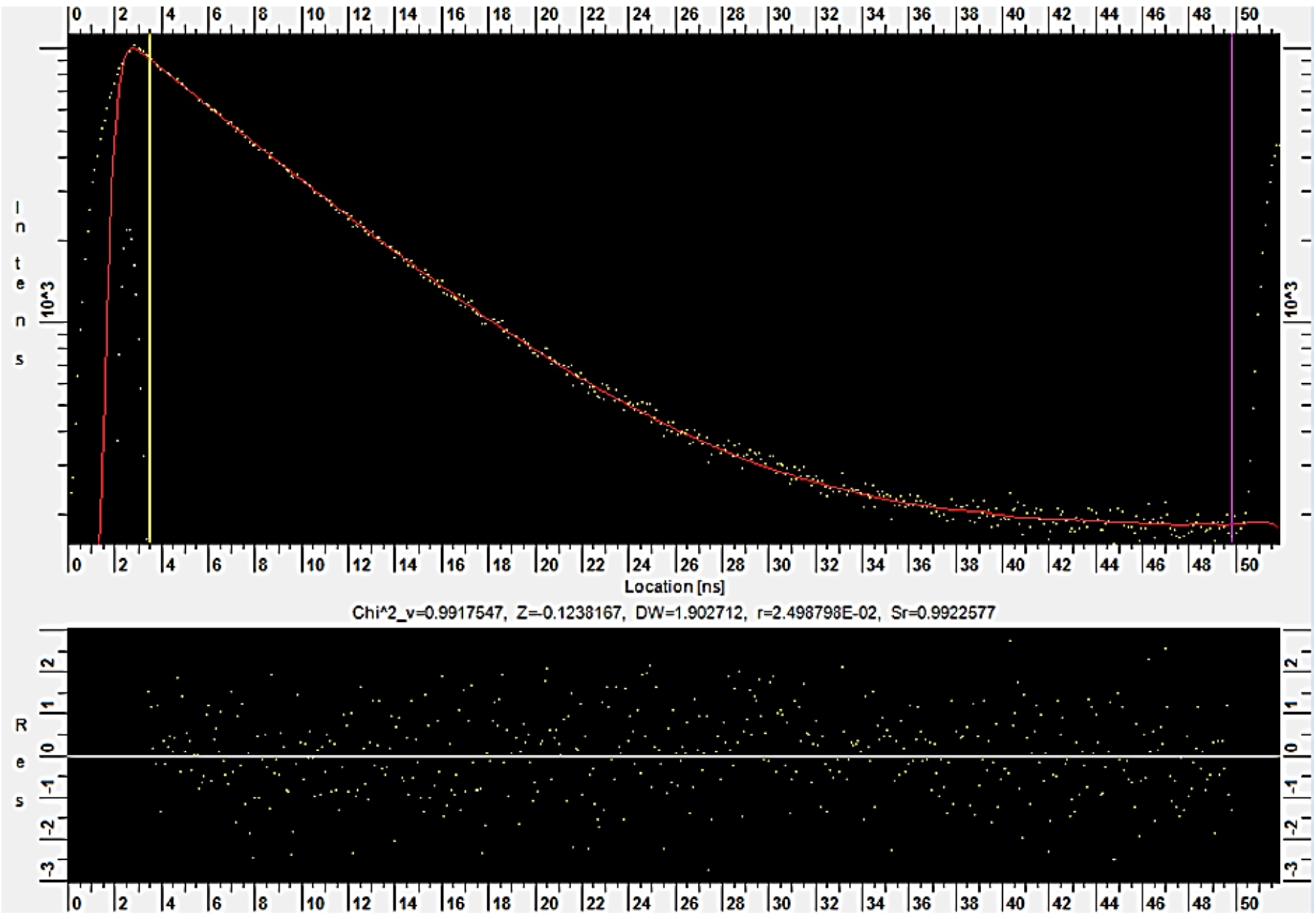

Figure S36: Emission-lifetime measurements in toluene of **H-FO-Ar** (red: monoexponential fit) and residual distribution of the fit.

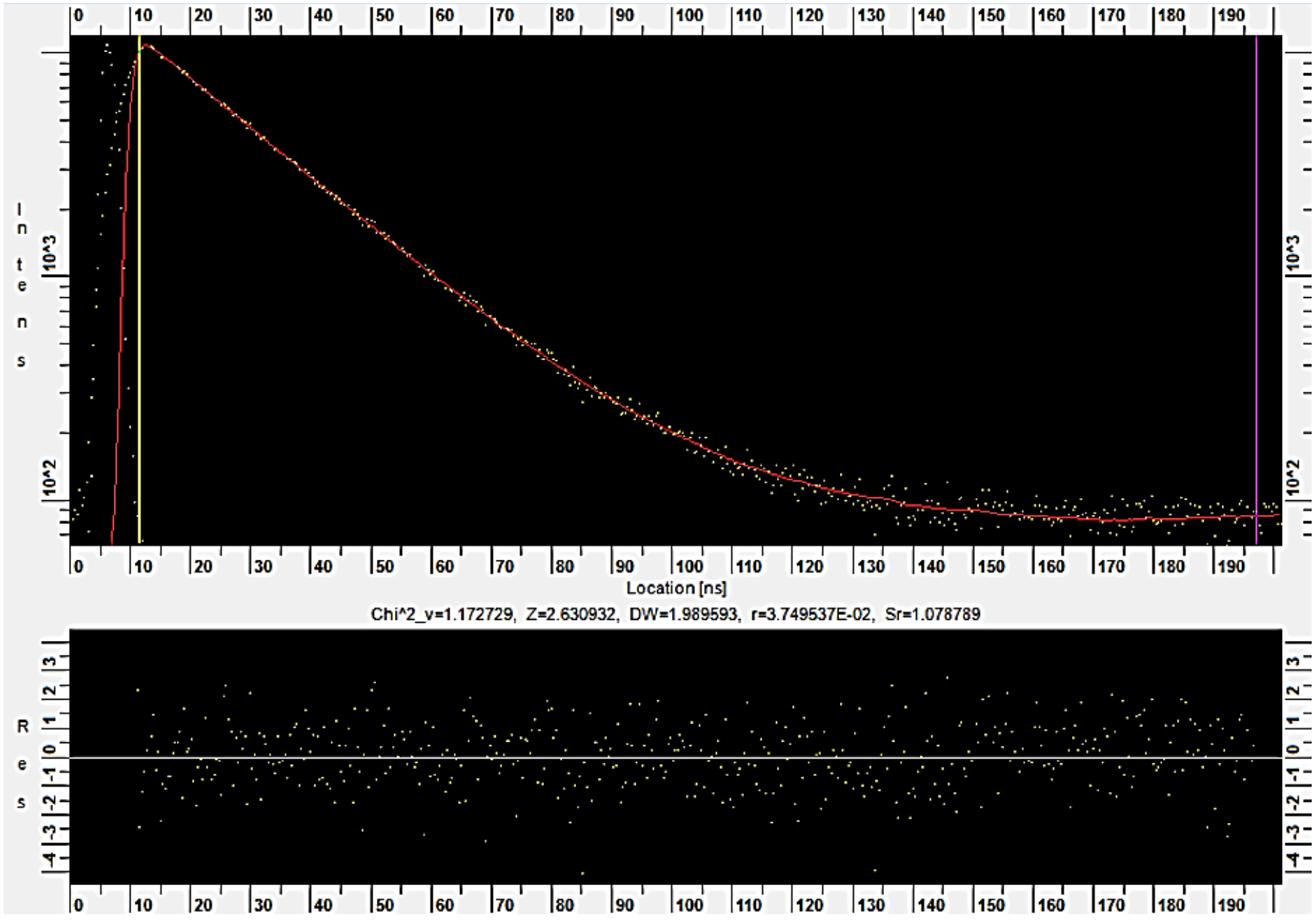

Figure S37: Emission-lifetime measurements in the solid state of **H-FO-Ar** (red: monoexponential fit) and residual distribution of the fit.

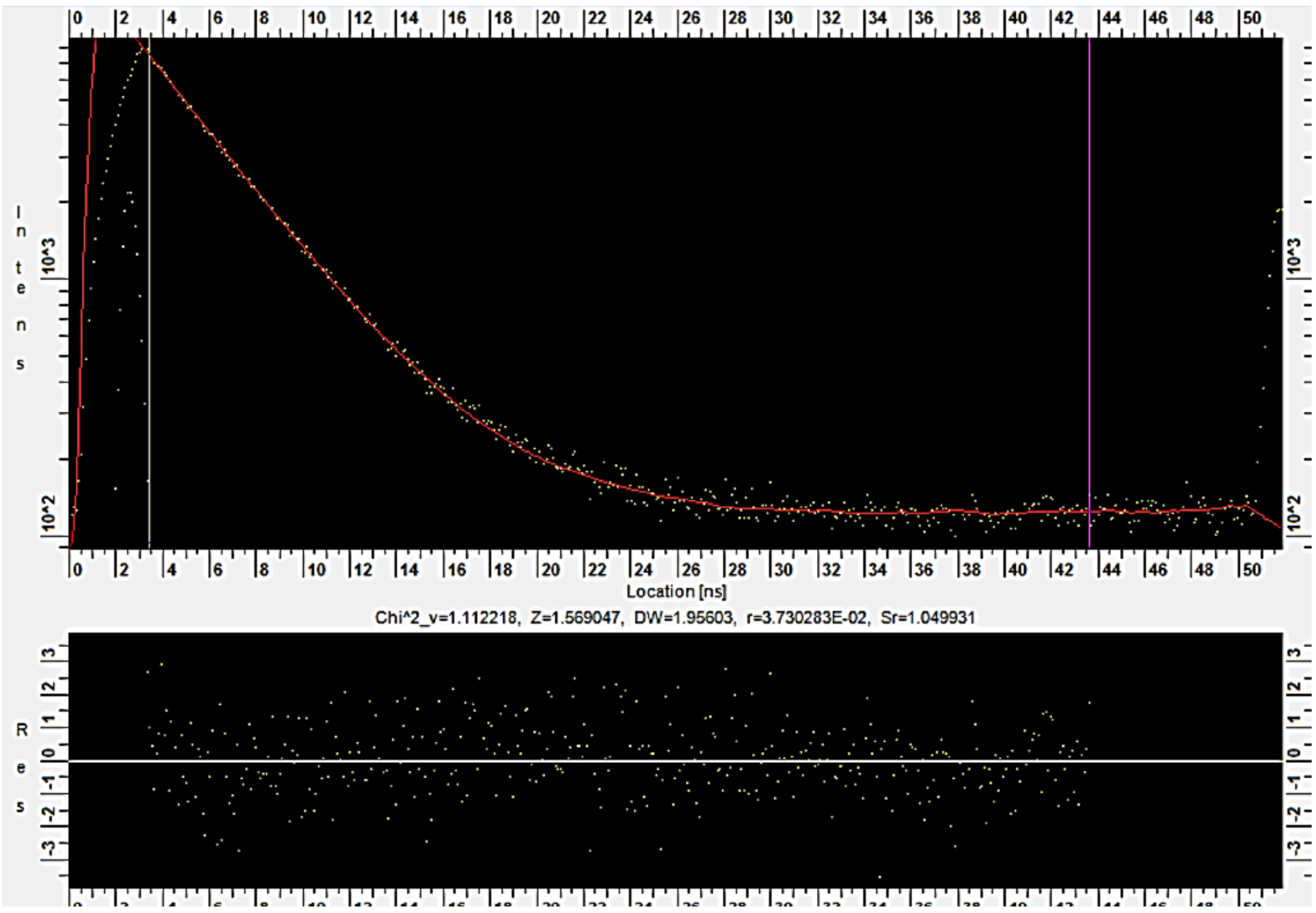

Figure S38: Emission-lifetime measurements in toluene of **Br-FO-Ar** (red: monoexponential fit) and residual distribution of the fit.

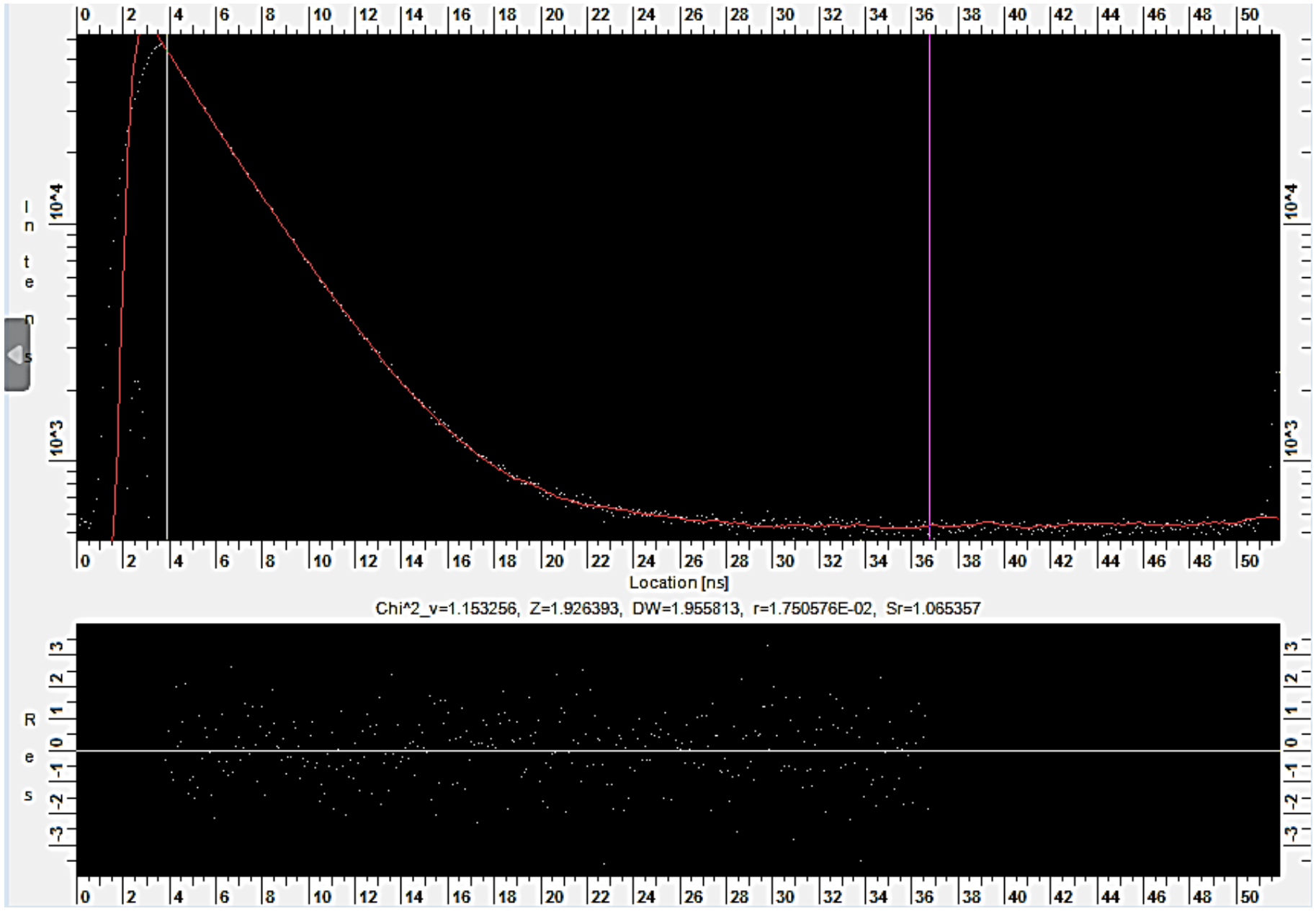

Figure S39: Emission-lifetime measurements in the solid state of **Br-FO-Ar** (red: biexponential fit) and residual distribution of the fit.

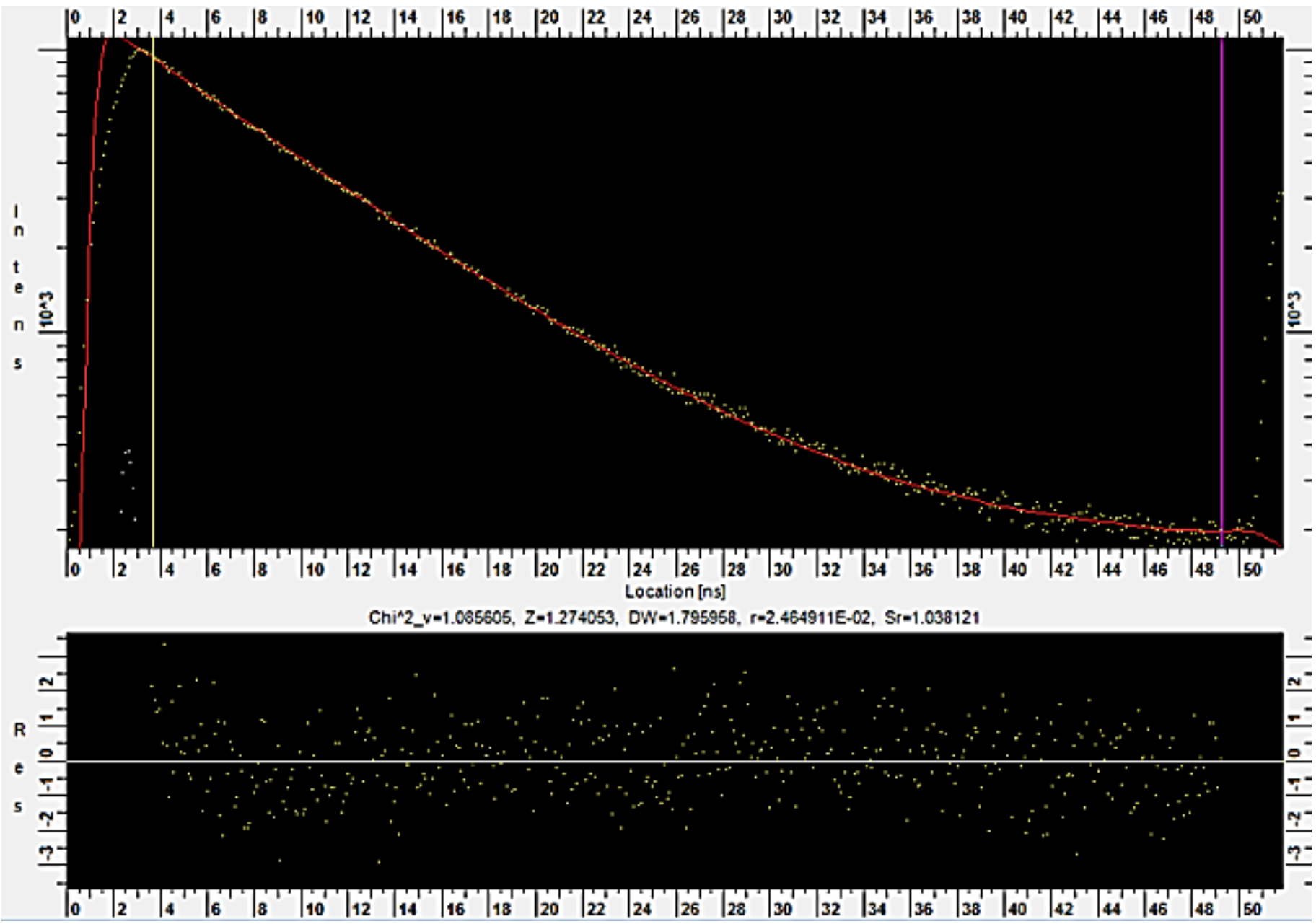

Figure S40: Emission-lifetime measurements in toluene of **NO_2-FO-Ar** (red: monoexponential fit) and residual distribution of the fit.

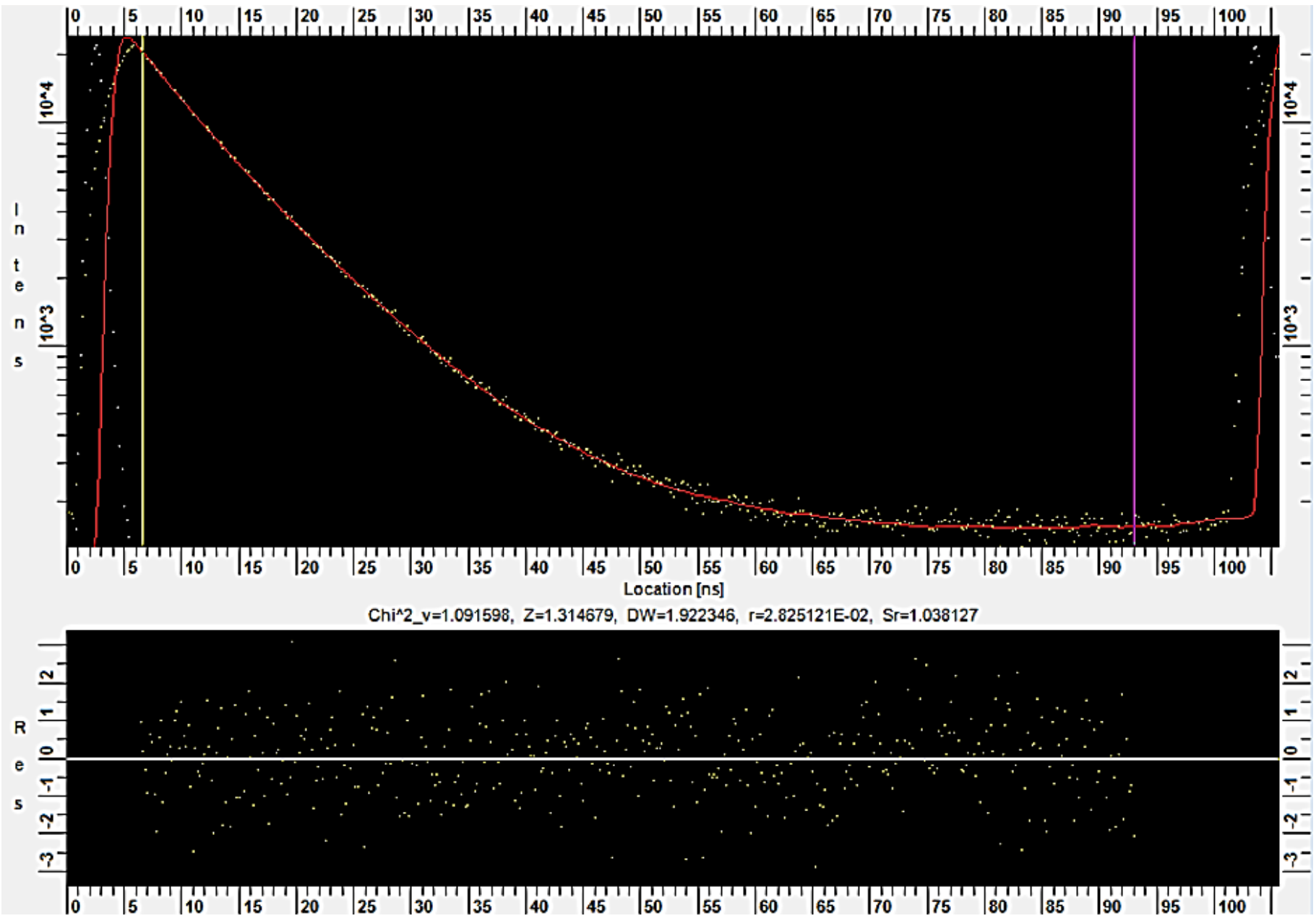

Figure S41: Emission-lifetime measurements in the solid state of **NO_2-FO-Ar** (red: biexponential fit) and residual distribution of the fit.

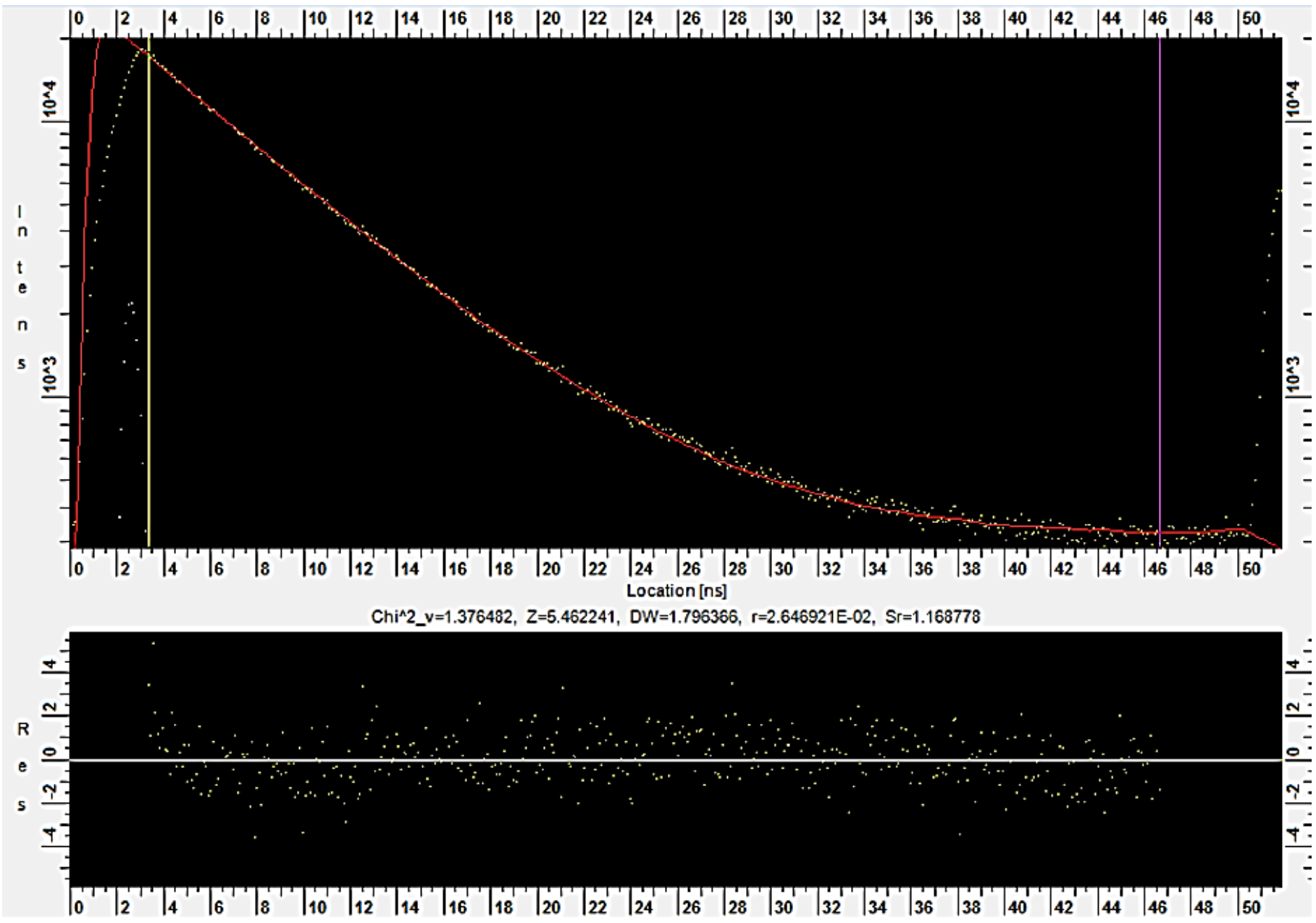

Figure S42: Emission-lifetime measurements in toluene of **CN-FO-Ar** (red: monoexponential fit) and residual distribution of the fit.

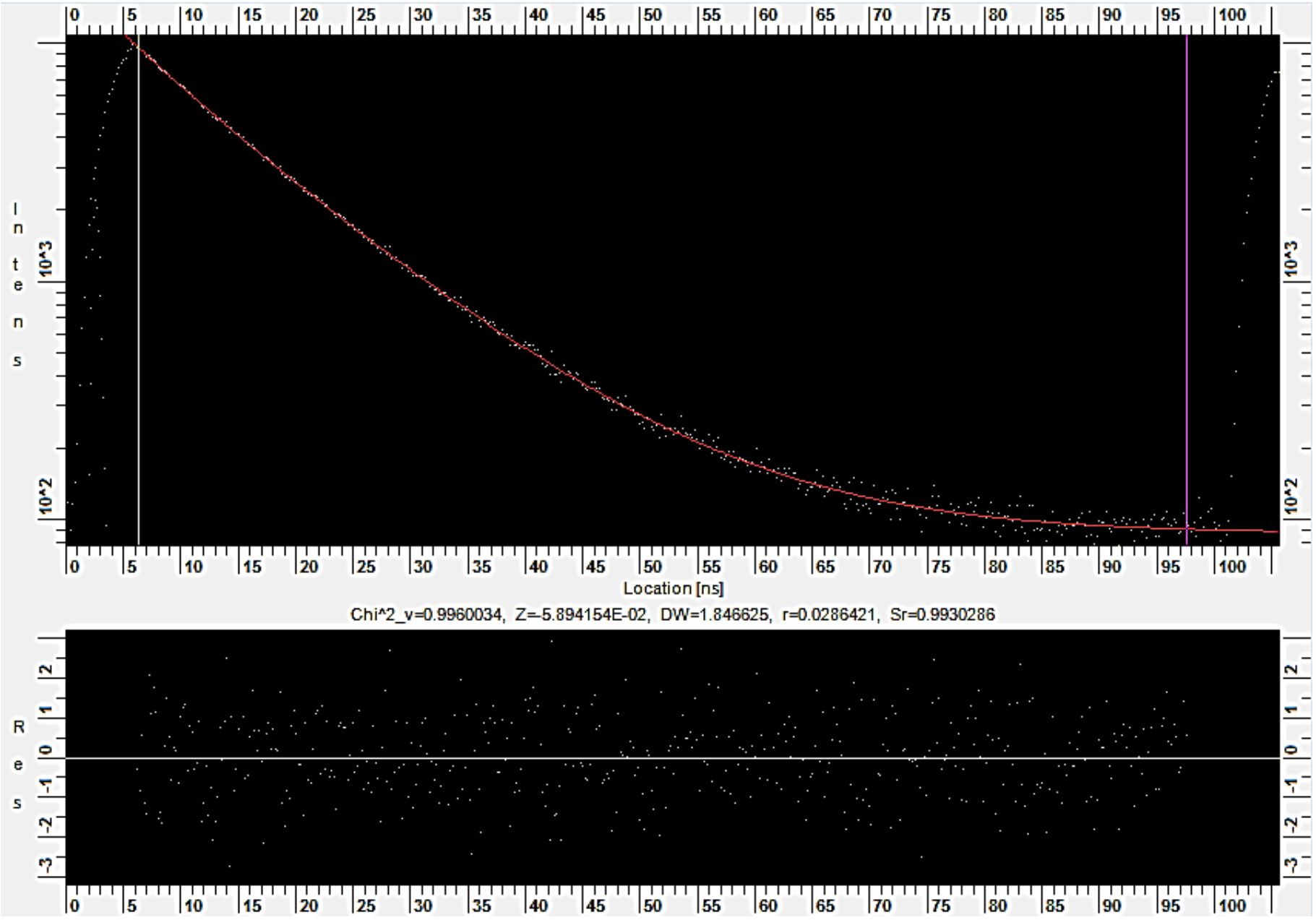

Figure S43: Emission-lifetime measurements in the solid state of **CN-FO-Ar** (red: biexponential fit) and residual distribution of the fit.

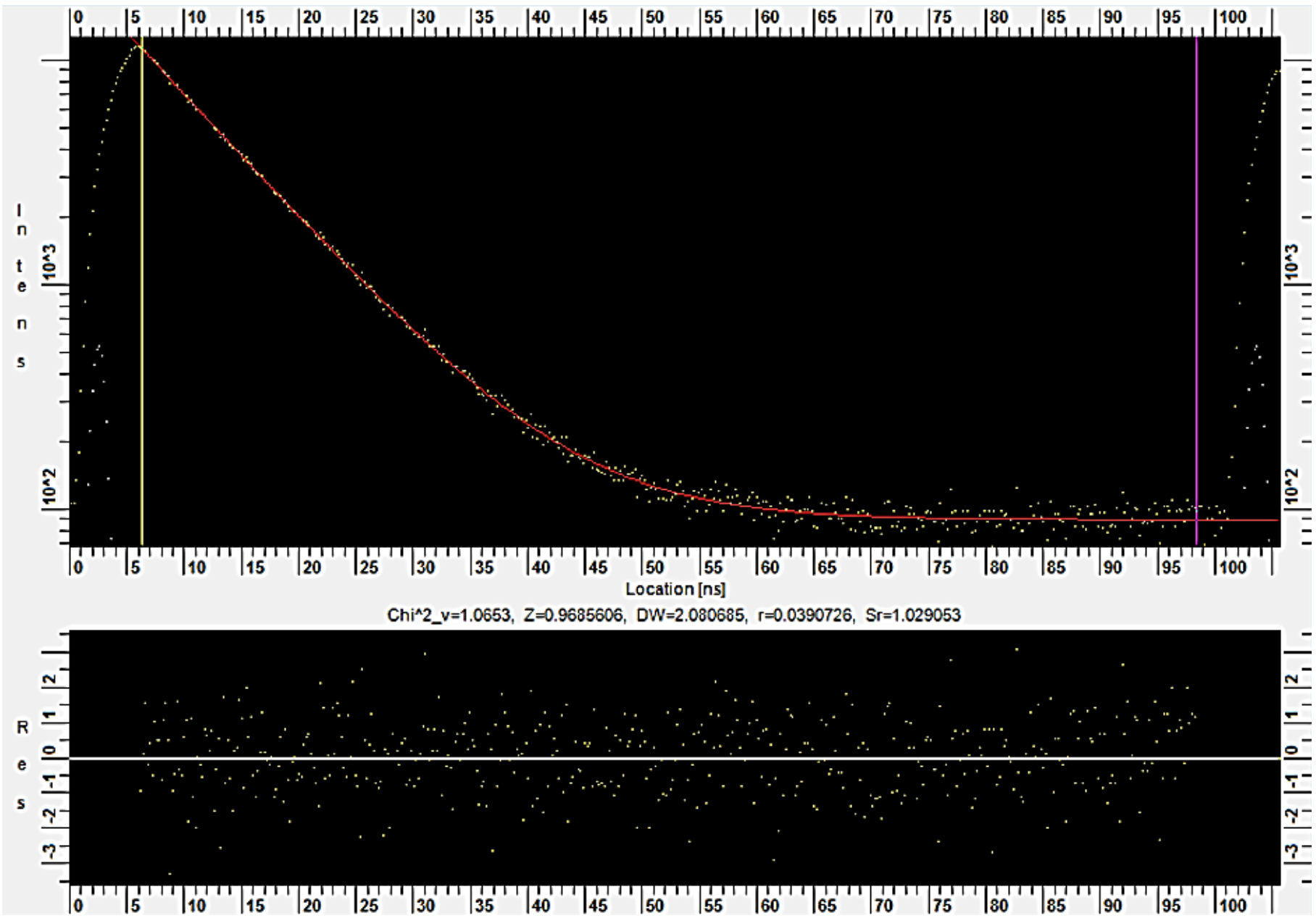

Figure S44: Emission-lifetime measurements in the solid state of **CN-FO-Ar^{h}** (red: monoexponential fit) and residual distribution of the fit.

4.4. Temperature dependent emission

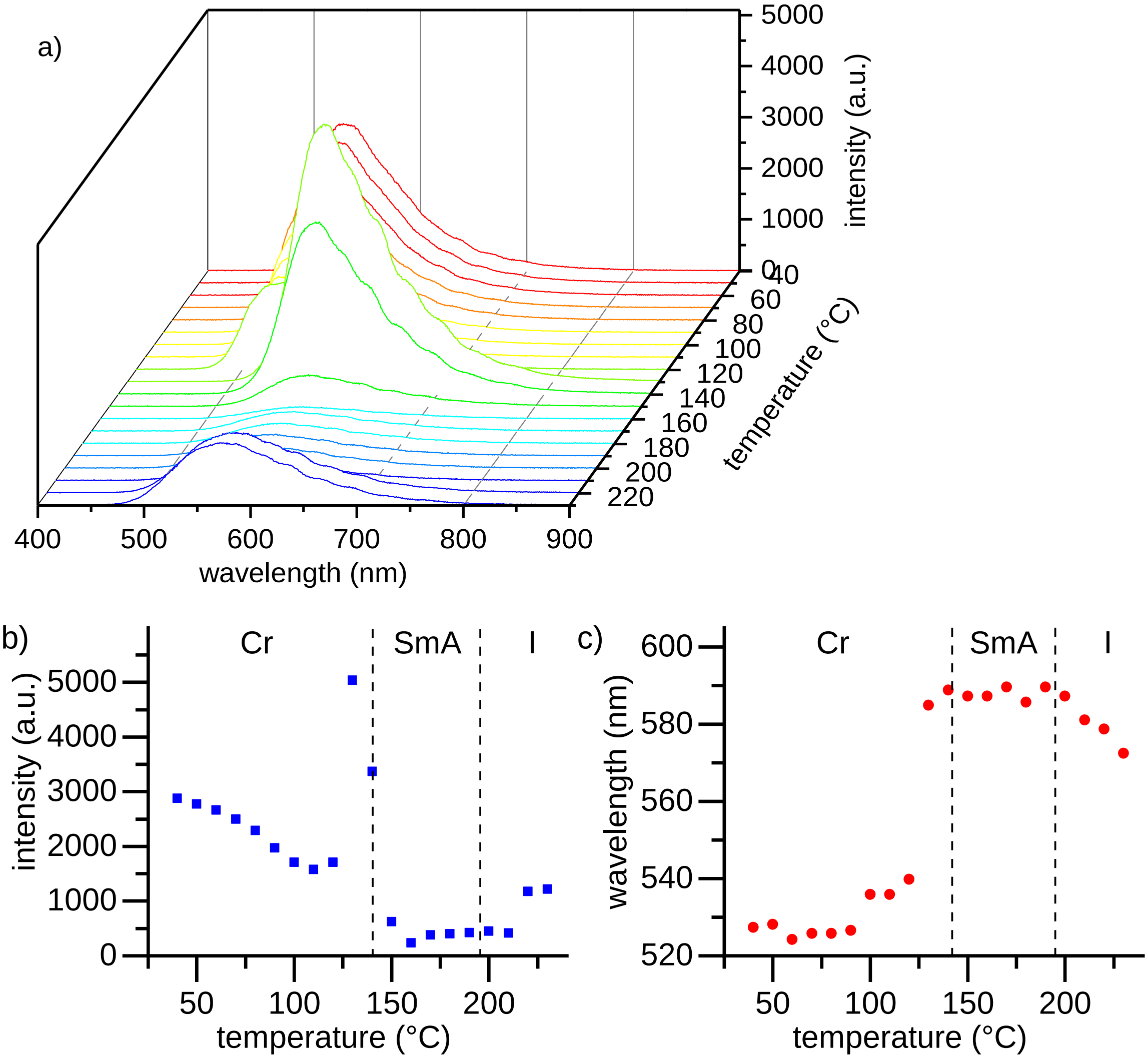

Figure S45: CN-FO-Ar a) temperature dependent emission spectra, b) intensities and c) wavelengths of emission maxima against the temperature with indicated phase transition temperatures.

5. Crystal structure

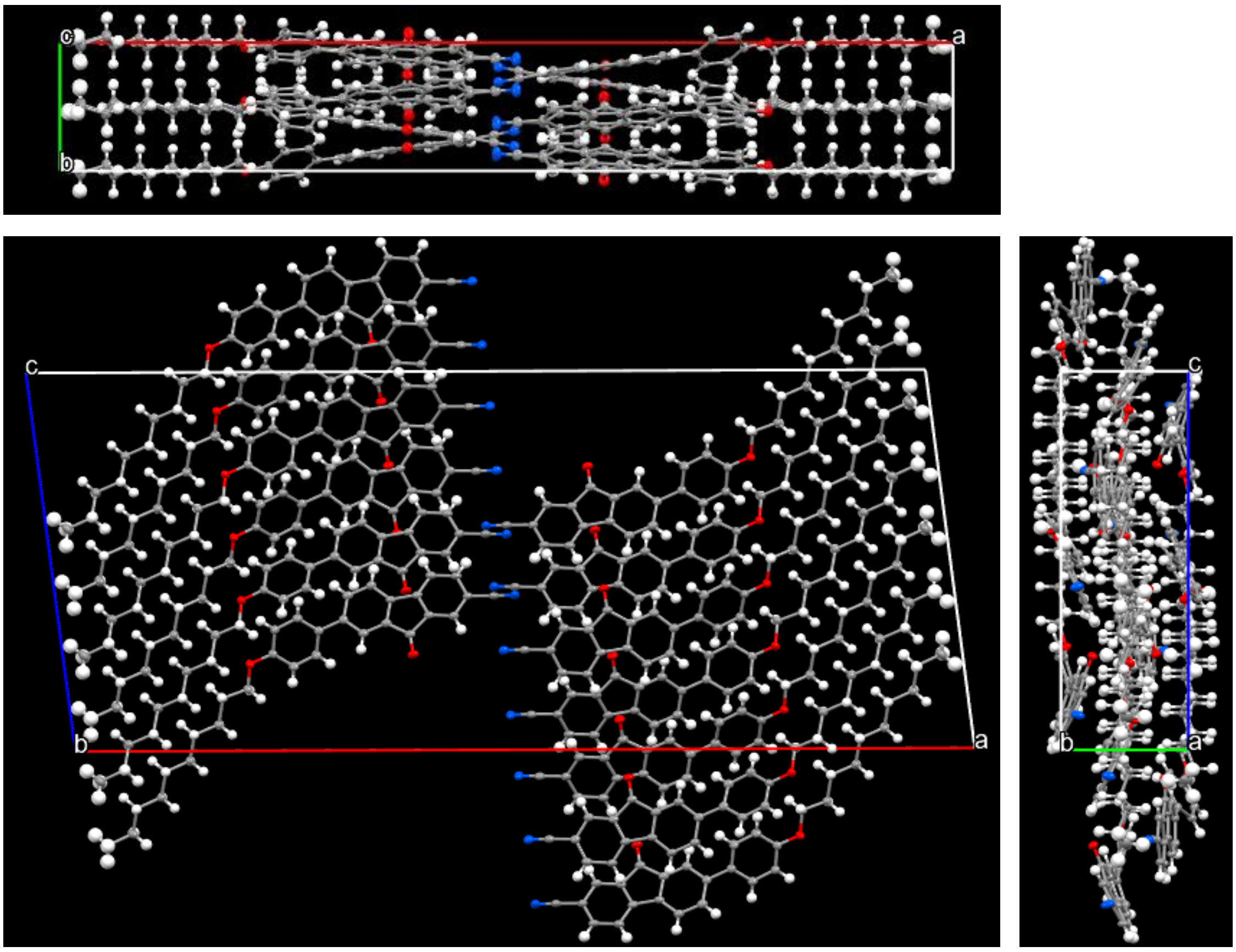

Figure S46: Crystal structure of **CN-FO-Ar** obtained from single crystal X-ray diffraction; top: front view (along c-axis), bottom left: top view (along b- axis), bottom right: side view (along c-axis). Displacement ellipsoids drawn at the 50% probability level. Color code: O red, N blue, C grey, H white.

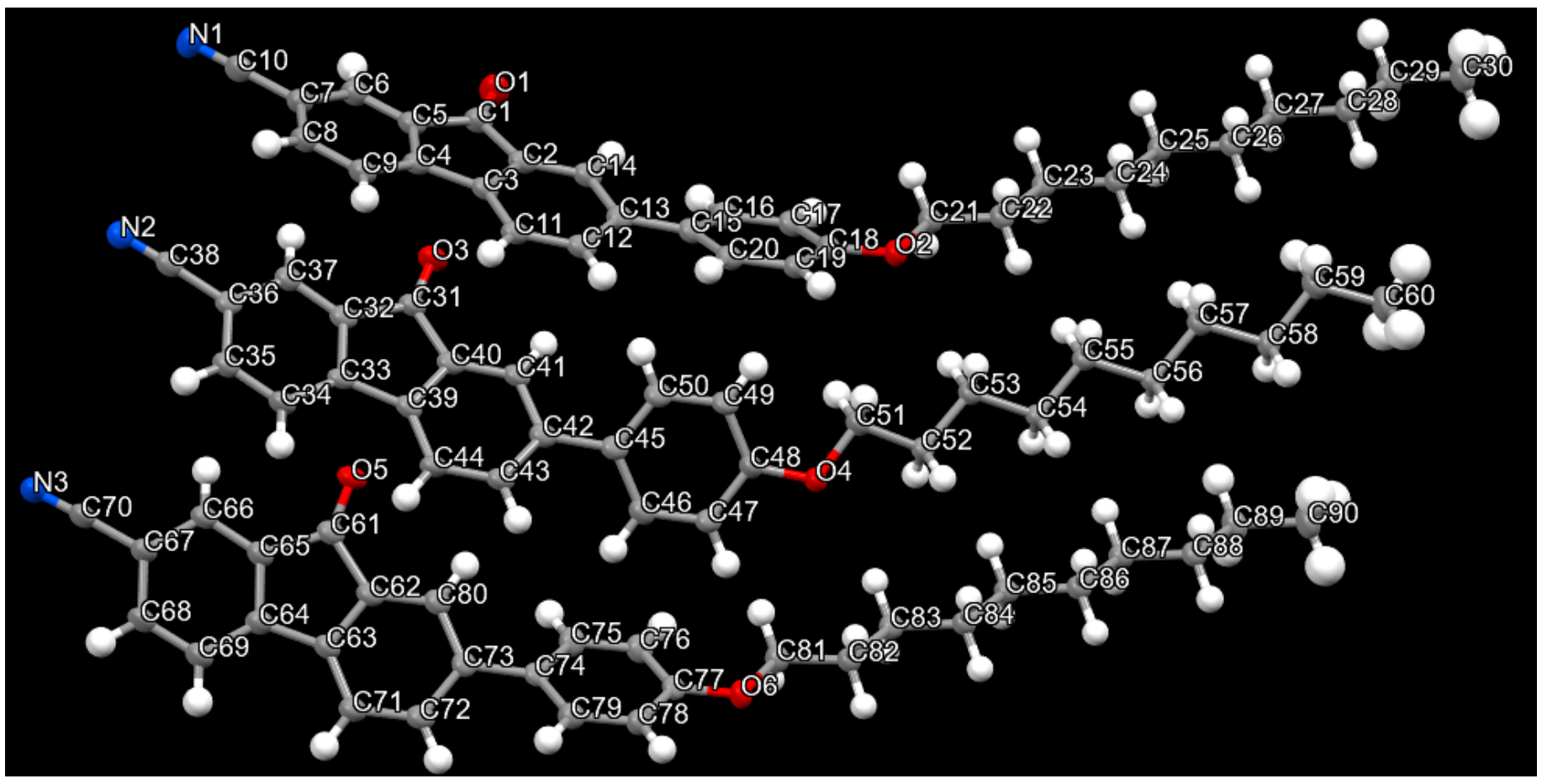

Figure S47: Structure of **CN-FO-Ar** with labeled atoms from single crystal X-ray diffraction. Displacement ellipsoids drawn at the 50% probability level. Color code: O red, N blue, C grey, H white.

Table S8: Crystal data and structure refinement for **CN-FO-Ar**.

CCDC number	2560662
Empirical formula	$C_{30}H_{31}NO_2$
Formula weight	437.56
Temperature/K	100.00(10)
Crystal system	monoclinic
Space group	$P2_1/c$
a/Å	49.2387(13)
b/Å	6.9724(2)
c/Å	20.9172(6)
α/°	90
β/°	97.208(3)
γ/°	90
Volume/Å^3	7124.4(3)
Z	12
ρ_{calc}g/cm^3	1.224
μ/mm^{-1}	0.589
F(000)	2808
Crystal size/mm^3	0.1 × 0.1 × 0.1
Radiation	Cu Kα (λ = 1.54184)
2Θ range for data collection/°	5.428 to 158.974
Index ranges	-61 ≤ h ≤ 61, -6 ≤ k ≤ 8, -25 ≤ l ≤ 26
Reflections collected	66427
Independent reflections	14648 [R_{int} = 0.0676, R_{sigma} = 0.0354]
Data/restraints/parameters	14648/0/895
Goodness-of-fit on F^2	1.101
Final R indexes [I>=2σ (I)]	R_1 = 0.1233, wR_2 = 0.3263
Final R indexes [all data]	R_1 = 0.1325, wR_2 = 0.3319
Largest diff. peak/hole / e Å^{-3}	0.60/-0.49

Table S9: Fractional Atomic Coordinates (×10^4) and Equivalent Isotropic Displacement Parameters (Å^2×10^3) for **CN-FO-Ar**. U_{eq} is defined as 1/3 of the trace of the orthogonalised U_{IJ} tensor.

Atom	*x*	*y*	*z*	U(eq)
O6	7931.6(5)	9590(4)	2709.2(13)	28.8(6)
O4	7927.5(5)	4999(4)	4364.3(13)	30.1(6)
O2	7935.9(5)	-304(4)	6029.1(13)	29.6(6)
O3	6109.1(6)	3207(5)	4257.2(13)	34.8(7)
O5	6105.1(6)	7529(4)	2475.4(13)	33.4(6)
O1	6093.2(6)	707(5)	5791.4(14)	42.2(8)
N2	4909.4(7)	1910(6)	2623.3(16)	34.3(8)

N3	4890.5(7)	8674(7)	916.7(17)	41.8(10)
C77	7662.2(7)	9684(5)	2445.4(18)	23.9(7)
N1	4892.6(7)	-1258(7)	4308.6(17)	41.5(9)
C74	7119.2(8)	9545(5)	1837.7(18)	24.5(7)
C48	7663.9(8)	4633(6)	4090.9(18)	27.6(8)
C86	9023.0(8)	9600(6)	5544.5(17)	26.1(8)
C73	6828.5(8)	9439(5)	1545.0(18)	25.8(8)
C23	8359.6(7)	426(6)	7631.6(18)	25.5(8)
C54	8645.3(8)	5352(6)	6232.8(18)	26.3(8)
C24	8643.9(7)	-265(6)	7909.6(17)	24.8(8)
C53	8364.9(8)	4584(6)	5961.6(18)	26.4(8)
C56	9017.2(8)	5438(6)	7203.5(17)	25.1(8)
C26	9017.3(8)	-295(6)	8867.6(17)	26.0(8)
C3	6279.5(8)	-1276(6)	4356.7(17)	26.5(8)
C83	8366.5(8)	10236(6)	4301.0(18)	25.7(8)
C88	9396.6(8)	9757(6)	6512.3(18)	27.5(8)
C57	9107.5(8)	4725(6)	7883.4(18)	27.6(8)
C82	8281.3(8)	9563(6)	3612.4(18)	26.2(8)
C22	8274.1(8)	-300(6)	6947.7(18)	26.3(8)
C38	5140.0(8)	2208(6)	2637.8(18)	29.7(8)
C84	8651.7(7)	9570(6)	4577.3(17)	26.1(8)
C27	9108.9(8)	399(6)	9550.1(18)	27.2(8)
C39	6283.0(8)	3409(5)	2673.3(17)	25.9(8)
C87	9110.3(8)	10351(6)	6223.3(18)	27.0(8)
C18	7668.9(8)	-232(6)	5750.7(18)	29.7(8)
C47	7583.7(8)	5481(6)	3487.9(19)	29.6(8)
C55	8735.2(8)	4712(6)	6922.5(18)	26.9(8)
C46	7317.7(8)	5295(6)	3196.5(18)	29.3(8)
C62	6357.7(8)	8827(6)	1645.5(18)	28.3(8)
C65	5872.6(8)	8572(6)	1432.3(18)	28.8(8)
C4	5978.2(8)	-1386(6)	4237.7(17)	27.3(8)
C28	9394.0(8)	-263(6)	9827.4(18)	27.9(8)
C78	7606.0(8)	9093(6)	1804.6(19)	29.2(8)
C63	6274.1(8)	9508(6)	1021.8(18)	28.3(8)
C36	5428.1(8)	2582(5)	2630.4(18)	26.1(8)
C76	7448.6(8)	10248(6)	2772.5(19)	30.7(8)
C85	8739.2(8)	10267(6)	5262.1(18)	26.7(8)
C58	9394.1(8)	5365(6)	8162.8(19)	29.4(8)
C75	7182.0(8)	10158(6)	2474.5(19)	30.3(8)
C52	8274.1(8)	5197(6)	5271.2(18)	27.3(8)
C25	8733.8(8)	412(6)	8594.1(18)	27.0(8)
C79	7338.1(8)	9047(6)	1505.2(19)	29.8(8)
C32	5881.6(8)	2965(5)	3158.0(17)	26.4(8)
C70	5122.5(9)	8779(7)	933.4(18)	32.5(9)

C81	7991.6(8)	10177(6)	3371.8(18)	28.5(8)
C67	5415.1(8)	8957(6)	935.9(18)	30.2(8)
C40	6363.4(8)	3429(5)	3342.7(18)	27.2(8)
C17	7464.5(8)	760(6)	5997(2)	31.8(9)
C15	7124.5(8)	-531(6)	5149.5(19)	29.5(8)
C45	7122.7(8)	4182(6)	3482.8(19)	28.3(8)
C64	5973.6(8)	9414(6)	900.5(18)	29.9(8)
C41	6629.8(8)	3685(6)	3602.9(18)	27.9(8)
C80	6627.4(8)	8799(6)	1907.2(19)	29.0(8)
C19	7605.5(8)	-1306(6)	5181.8(19)	30.4(8)
C7	5417.2(8)	-1244(6)	4286.8(18)	30.8(9)
C33	5982.9(8)	3130(5)	2558.9(17)	25.8(8)
C10	5124.9(9)	-1232(7)	4300.8(19)	33.8(9)
C21	7993.0(8)	402(6)	6678.3(18)	27.4(8)
C43	6751.2(8)	3971(6)	2530.2(18)	29.5(8)
C66	5598.4(8)	8300(6)	1459.6(18)	29.9(8)
C51	7999.0(8)	4340(6)	5017.3(18)	29.1(8)
C11	6484.2(8)	-1716(6)	3981.5(18)	28.7(8)
C2	6356.7(8)	-490(6)	4975.7(18)	29.1(8)
C13	6833.4(8)	-741(6)	4870.9(19)	28.3(8)
C72	6744.8(9)	10065(6)	907.2(19)	30.9(8)
C5	5869.4(8)	-599(6)	4774.0(19)	30.6(8)
C14	6622.4(8)	-253(6)	5227.8(19)	29.9(8)
C42	6833.3(8)	3978(6)	3198.0(18)	28.2(8)
C9	5801.9(8)	-2126(6)	3727.1(18)	30.3(8)
C44	6479.8(8)	3702(6)	2263.1(18)	29.5(8)
C8	5521.3(9)	-2071(6)	3756.2(18)	32.0(9)
C31	6116.8(8)	3192(6)	3678.7(17)	27.7(8)
C71	6471.7(8)	10138(6)	648.3(19)	30.9(8)
C61	6110.2(8)	8184(6)	1936.4(18)	29.3(8)
C12	6756.4(9)	-1459(6)	4240.4(19)	30.6(8)
C16	7197.2(8)	610(6)	5700.6(19)	31.7(9)
C34	5802.4(8)	3076(6)	1995.4(18)	30.2(8)
C20	7338.3(8)	-1465(6)	4892.6(19)	29.7(8)
C37	5609.6(8)	2692(6)	3207.0(18)	28.8(8)
C1	6102.7(9)	-5(7)	5263.7(19)	33.0(9)
C6	5594.1(9)	-485(7)	4804.1(19)	33.4(9)
C49	7476.8(8)	3484(6)	4370(2)	30.7(8)
C35	5525.7(8)	2805(6)	2034.3(18)	30.9(9)
C69	5790.7(9)	10089(7)	386.7(19)	33.3(9)
C50	7211.7(8)	3294(6)	4071.4(19)	29.7(8)
C68	5513.1(9)	9877(7)	406.0(18)	33.9(9)
C29	9480.8(9)	485(7)	10504(2)	36.8(10)
C59	9479.9(9)	4565(7)	8836(2)	37.7(10)

C89	9478.9(9)	10577(7)	7179(2)	36.8(10)
C90	9765.3(10)	9994(8)	7478(2)	47.2(12)
C30	9765.4(9)	-165(9)	10794(2)	47.8(12)
C60	9766.8(10)	5173(9)	9127(2)	48.5(12)

Table S10: Anisotropic Displacement Parameters ($Å^2 \times 10^3$) for **CN-FO-Ar**. The Anisotropic displacement factor exponent takes the form: $-2\pi^2[h^2a^{*2}U_{11}+2hka^*b^*U_{12}+\ldots]$.

Atom	U_{11}	U_{22}	U_{33}	U_{23}	U_{13}	U_{12}
O6	25.3(14)	34.1(15)	25.8(13)	-6.4(11)	-1.0(10)	2.3(11)
O4	25.6(14)	38.4(16)	25.4(14)	4.3(12)	0.1(10)	-1.7(11)
O2	25.0(14)	37.0(16)	25.7(13)	-0.8(12)	-0.4(10)	0.4(11)
O3	41.1(16)	44.5(18)	19.5(13)	-1.5(12)	5.8(11)	0.5(13)
O5	42.3(16)	33.5(16)	24.9(14)	6.1(12)	6.4(11)	2.0(12)
O1	40.9(17)	62(2)	24.1(14)	-12.0(14)	6.6(12)	-0.1(15)
N2	37(2)	38(2)	27.8(17)	-3.5(15)	4.6(14)	0.4(15)
N3	31(2)	67(3)	26.9(18)	11.5(18)	3.1(14)	0.4(18)
C77	27.8(19)	17.7(17)	25.1(18)	-0.4(14)	-0.2(14)	2.4(13)
N1	31(2)	64(3)	29.2(18)	-9.4(18)	4.0(14)	-5.2(17)
C74	29.8(19)	17.9(17)	25.6(18)	-0.1(14)	2.4(14)	2.9(14)
C48	26.2(19)	34(2)	22.3(18)	2.3(16)	0.7(14)	0.2(15)
C86	30(2)	26(2)	21.3(18)	-3.7(15)	2.9(14)	-1.7(15)
C73	30(2)	20.0(18)	26.5(18)	-2.7(15)	2.2(14)	2.1(14)
C23	27.5(19)	25.1(19)	23.7(18)	-3.3(15)	2.1(14)	-1.3(14)
C54	27.4(19)	27(2)	24.3(18)	3.3(15)	2.4(14)	-1.0(15)
C24	28.0(19)	24.8(19)	21.5(17)	-2.5(14)	2.7(14)	-1.1(14)
C53	27.3(19)	27(2)	24.0(18)	1.1(15)	0.1(14)	-0.7(15)
C56	29.9(19)	23.7(19)	21.5(17)	-0.7(14)	2.7(14)	0.1(14)
C26	31(2)	24.6(19)	21.9(18)	-2.0(15)	1.9(14)	2.3(15)
C3	37(2)	23.0(19)	19.6(17)	-0.1(14)	3.0(14)	1.4(15)
C83	29.1(19)	22.9(19)	25.0(18)	-3.9(15)	2.5(14)	0.0(14)
C88	31(2)	25.3(19)	25.9(19)	-2.0(15)	1.4(15)	-1.6(15)
C57	30(2)	28(2)	23.8(18)	0.0(15)	0.9(14)	-0.1(15)
C82	27.7(19)	27(2)	23.3(18)	-1.5(15)	1.2(14)	-0.2(15)
C22	29(2)	25.7(19)	24.2(18)	-1.8(15)	1.1(14)	0.3(15)
C38	38(2)	30(2)	20.8(17)	-1.3(15)	3.0(15)	4.0(16)
C84	27.3(19)	29(2)	21.6(18)	0.0(15)	2.2(14)	-0.2(15)
C27	29(2)	29(2)	22.8(18)	-1.3(15)	2.2(14)	1.2(15)
C39	37(2)	21.9(18)	18.7(17)	-0.1(14)	1.3(14)	2.3(15)
C87	30(2)	29(2)	22.1(18)	-1.6(15)	2.9(14)	0.5(15)
C18	27(2)	37(2)	23.9(18)	1.8(16)	-0.1(14)	-2.6(16)
C47	31(2)	27(2)	31(2)	3.5(16)	5.7(15)	0.5(15)
C55	27.7(19)	28(2)	25.2(19)	1.3(15)	2.6(14)	-2.3(15)
C46	34(2)	31(2)	22.9(18)	-1.1(16)	1.6(15)	3.0(16)
C62	38(2)	24.0(19)	23.4(18)	0.2(15)	3.8(15)	-0.4(15)

C65	35(2)	30(2)	20.8(17)	-2.5(15)	2.8(15)	2.3(16)
C4	38(2)	24.6(19)	19.3(17)	-0.3(15)	5.0(15)	-1.4(15)
C28	31(2)	28(2)	23.9(18)	-2.6(15)	0.8(15)	0.7(15)
C78	29(2)	31(2)	27.6(19)	-3.6(16)	4.5(15)	1.1(16)
C63	36(2)	29(2)	19.1(17)	-1.2(15)	1.5(14)	-0.5(16)
C36	35(2)	19.6(18)	24.3(18)	-2.6(14)	5.0(15)	-0.2(14)
C76	35(2)	29(2)	27.4(19)	-7.9(16)	2.2(15)	2.9(16)
C85	30(2)	28(2)	22.6(18)	-0.6(15)	2.0(14)	-1.3(15)
C58	27(2)	33(2)	27.6(19)	-3.0(16)	-0.4(15)	0.0(15)
C75	32(2)	28(2)	31(2)	-8.4(16)	2.5(15)	4.6(16)
C52	25.9(19)	29(2)	26.4(19)	3.6(16)	1.8(14)	0.0(15)
C25	27.2(19)	29(2)	24.5(18)	-3.5(15)	1.2(14)	0.6(15)
C79	35(2)	30(2)	24.9(18)	-5.2(16)	3.3(15)	-1.1(16)
C32	38(2)	20.7(18)	21.0(17)	-2.8(14)	4.4(15)	-0.3(15)
C70	37(2)	42(2)	19.0(17)	2.6(17)	1.5(15)	-0.4(18)
C81	29(2)	31(2)	24.6(19)	-6.1(16)	0.0(15)	0.0(15)
C67	33(2)	38(2)	20.3(17)	-0.2(16)	4.3(15)	-0.1(17)
C40	38(2)	20.4(18)	23.6(18)	0.8(15)	6.3(15)	2.1(15)
C17	35(2)	31(2)	30(2)	1.7(17)	4.1(16)	3.4(16)
C15	35(2)	26(2)	27.2(19)	1.1(16)	2.6(15)	1.4(16)
C45	35(2)	22.6(19)	28.1(19)	-0.4(15)	6.2(15)	2.0(15)
C64	37(2)	33(2)	20.3(18)	-4.3(16)	5.5(15)	-2.7(16)
C41	37(2)	25.0(19)	21.1(17)	2.6(15)	2.9(15)	0.4(15)
C80	36(2)	27(2)	24.2(18)	-2.0(15)	2.1(15)	0.7(16)
C19	32(2)	33(2)	26.9(19)	2.8(16)	5.0(15)	-1.0(16)
C7	33(2)	37(2)	23.0(18)	0.3(16)	4.5(15)	-0.7(17)
C33	39(2)	20.0(18)	19.7(17)	1.2(14)	7.4(14)	1.5(15)
C10	39(2)	41(2)	20.9(18)	-5.3(17)	2.2(16)	-3.5(18)
C21	29(2)	31(2)	22.0(18)	-2.2(15)	1.1(14)	-0.6(15)
C43	37(2)	29(2)	23.6(18)	3.1(16)	6.9(15)	0.9(16)
C66	37(2)	30(2)	22.1(18)	2.7(16)	4.0(15)	0.6(16)
C51	29(2)	37(2)	20.3(17)	3.0(16)	-0.5(14)	0.5(16)
C11	37(2)	27(2)	23.4(18)	0.6(15)	7.3(15)	1.5(16)
C2	39(2)	27(2)	22.0(18)	1.6(15)	6.3(15)	1.7(16)
C13	35(2)	22.3(19)	27.5(19)	-0.6(15)	3.7(15)	-0.4(15)
C72	40(2)	27(2)	26.7(19)	-0.4(16)	6.6(16)	-1.0(16)
C5	39(2)	29(2)	23.1(18)	1.1(16)	4.2(15)	-1.7(16)
C14	40(2)	27(2)	22.7(18)	-2.1(15)	5.0(15)	0.5(16)
C42	36(2)	23.4(19)	25.3(18)	-0.8(15)	5.2(15)	1.6(15)
C9	39(2)	31(2)	20.1(17)	-2.4(16)	2.5(15)	-2.1(17)
C44	36(2)	31(2)	21.8(18)	3.3(16)	7.4(15)	-0.1(16)
C8	39(2)	36(2)	20.2(17)	0.1(16)	1.2(15)	-2.7(17)
C31	37(2)	26(2)	19.9(17)	0.5(15)	4.4(15)	0.2(16)
C71	37(2)	31(2)	25.4(19)	1.3(16)	4.9(15)	-0.2(16)

C61	38(2)	26(2)	24.0(18)	0.2(15)	4.5(15)	2.5(16)
C12	41(2)	26(2)	25.5(19)	0.5(16)	7.8(16)	2.1(16)
C16	34(2)	32(2)	31(2)	-2.5(17)	6.1(16)	3.1(16)
C34	37(2)	35(2)	18.8(17)	1.5(16)	3.1(15)	-0.4(17)
C20	35(2)	28(2)	26.2(19)	0.2(16)	4.0(15)	0.0(16)
C37	37(2)	26(2)	24.1(18)	-0.7(15)	4.1(15)	1.4(16)
C1	39(2)	38(2)	23.1(19)	-2.3(17)	6.6(16)	-0.9(18)
C6	39(2)	39(2)	23.1(19)	-2.7(17)	4.9(16)	-2.5(18)
C49	35(2)	26(2)	31(2)	7.3(16)	3.3(16)	0.2(16)
C35	40(2)	30(2)	21.8(18)	-0.4(16)	0.2(15)	-1.4(17)
C69	40(2)	38(2)	21.1(18)	-0.1(17)	1.1(16)	-1.3(18)
C50	36(2)	22.9(19)	31(2)	0.3(16)	4.9(16)	-0.3(15)
C68	43(2)	41(2)	17.4(17)	0.6(17)	2.2(15)	-1.0(18)
C29	37(2)	40(2)	31(2)	-2.7(18)	-4.3(17)	2.7(18)
C59	37(2)	45(3)	29(2)	4.4(19)	-4.6(17)	-1.0(19)
C89	38(2)	40(2)	30(2)	-5.6(18)	-5.3(17)	3.2(18)
C90	39(3)	57(3)	41(3)	0(2)	-11(2)	-1(2)
C30	38(3)	63(3)	39(3)	0(2)	-8.7(19)	3(2)
C60	38(3)	60(3)	43(3)	0(2)	-11(2)	-1(2)

Table S11: Bond lengths for **CN-FO-Ar**.

Atom	Atom	Length/Å	Atom	Atom	Length/Å
O6	C77	1.373(4)	C62	C80	1.372(6)
O6	C81	1.440(4)	C62	C61	1.497(6)
O4	C48	1.375(4)	C65	C64	1.403(5)
O4	C51	1.442(4)	C65	C66	1.372(6)
O2	C18	1.370(5)	C65	C61	1.498(5)
O2	C21	1.439(4)	C4	C5	1.413(5)
O3	C31	1.215(4)	C4	C9	1.388(5)
O5	C61	1.220(5)	C28	C29	1.519(5)
O1	C1	1.217(5)	C78	C79	1.388(5)
N2	C38	1.151(5)	C63	C64	1.471(6)
N3	C70	1.141(5)	C63	C71	1.393(6)
C77	C78	1.396(5)	C36	C37	1.410(5)
C77	C76	1.382(5)	C36	C35	1.400(5)
N1	C10	1.146(5)	C76	C75	1.382(5)
C74	C73	1.486(5)	C58	C59	1.524(5)
C74	C75	1.396(5)	C52	C51	1.514(5)
C74	C79	1.398(5)	C32	C33	1.410(5)
C48	C47	1.404(5)	C32	C31	1.495(5)
C48	C49	1.402(5)	C32	C37	1.369(6)
C86	C87	1.524(5)	C70	C67	1.445(6)
C86	C85	1.520(5)	C67	C66	1.406(5)
C73	C80	1.394(6)	C67	C68	1.417(5)

C73	C72	1.415(5)	C40	C41	1.367(6)
C23	C24	1.525(5)	C40	C31	1.487(5)
C23	C22	1.526(5)	C17	C16	1.386(6)
C54	C53	1.523(5)	C15	C13	1.485(6)
C54	C55	1.522(5)	C15	C16	1.409(6)
C24	C25	1.520(5)	C15	C20	1.401(6)
C53	C52	1.519(5)	C45	C42	1.481(6)
C56	C57	1.520(5)	C45	C50	1.399(5)
C56	C55	1.525(5)	C64	C69	1.395(6)
C26	C27	1.521(5)	C41	C42	1.405(5)
C26	C25	1.523(5)	C19	C20	1.382(6)
C3	C4	1.475(6)	C7	C10	1.444(6)
C3	C11	1.387(5)	C7	C8	1.404(6)
C3	C2	1.413(5)	C7	C6	1.405(6)
C83	C82	1.523(5)	C33	C34	1.385(5)
C83	C84	1.523(5)	C43	C42	1.405(5)
C88	C87	1.520(5)	C43	C44	1.395(6)
C88	C89	1.515(5)	C11	C12	1.393(6)
C57	C58	1.525(5)	C2	C14	1.358(6)
C82	C81	1.514(5)	C2	C1	1.493(6)
C22	C21	1.509(5)	C13	C14	1.395(6)
C38	C36	1.444(6)	C13	C12	1.417(5)
C84	C85	1.523(5)	C72	C71	1.386(6)
C27	C28	1.522(5)	C5	C1	1.498(6)
C39	C40	1.407(5)	C5	C6	1.367(6)
C39	C33	1.480(5)	C9	C8	1.391(6)
C39	C44	1.388(5)	C34	C35	1.388(6)
C18	C17	1.373(6)	C49	C50	1.381(6)
C18	C19	1.408(6)	C69	C68	1.381(6)
C47	C46	1.379(5)	C29	C30	1.524(6)
C46	C45	1.424(6)	C59	C60	1.526(6)
C62	C63	1.401(5)	C89	C90	1.524(6)

Table S12: Bond angles for **CN-FO-Ar**.

Atom	Atom	Atom	Angle/°	Atom	Atom	Atom	Angle/°
C77	O6	C81	116.4(3)	C66	C67	C70	121.1(4)
C48	O4	C51	116.1(3)	C66	C67	C68	120.6(4)
C18	O2	C21	116.9(3)	C68	C67	C70	118.2(4)
O6	C77	C78	115.9(3)	C39	C40	C31	109.0(3)
O6	C77	C76	124.9(3)	C41	C40	C39	122.3(4)
C76	C77	C78	119.2(3)	C41	C40	C31	128.8(3)
C75	C74	C73	119.7(3)	C18	C17	C16	119.6(4)
C75	C74	C79	117.3(3)	C16	C15	C13	120.7(4)
C79	C74	C73	123.1(3)	C20	C15	C13	122.6(4)

O4	C48	C47	116.1(3)	C20	C15	C16	116.7(4)
O4	C48	C49	124.6(3)	C46	C45	C42	123.3(3)
C49	C48	C47	119.3(4)	C50	C45	C46	117.3(4)
C85	C86	C87	113.3(3)	C50	C45	C42	119.4(4)
C80	C73	C74	120.3(3)	C65	C64	C63	109.3(3)
C80	C73	C72	117.9(4)	C69	C64	C65	119.6(4)
C72	C73	C74	121.6(3)	C69	C64	C63	131.0(4)
C24	C23	C22	112.6(3)	C40	C41	C42	120.0(4)
C55	C54	C53	113.1(3)	C62	C80	C73	119.8(4)
C25	C24	C23	113.9(3)	C20	C19	C18	120.7(4)
C52	C53	C54	113.6(3)	C8	C7	C10	118.9(4)
C57	C56	C55	113.4(3)	C8	C7	C6	120.8(4)
C27	C26	C25	113.5(3)	C6	C7	C10	120.4(4)
C11	C3	C4	132.8(3)	C32	C33	C39	108.9(3)
C11	C3	C2	118.2(4)	C34	C33	C39	131.5(3)
C2	C3	C4	108.9(3)	C34	C33	C32	119.6(4)
C84	C83	C82	113.3(3)	N1	C10	C7	178.7(5)
C89	C88	C87	113.0(3)	O2	C21	C22	107.6(3)
C56	C57	C58	114.3(3)	C44	C43	C42	122.7(4)
C81	C82	C83	111.3(3)	C65	C66	C67	117.5(4)
C21	C22	C23	112.0(3)	O4	C51	C52	107.9(3)
N2	C38	C36	177.9(4)	C3	C11	C12	118.9(4)
C85	C84	C83	113.5(3)	C3	C2	C1	108.4(3)
C26	C27	C28	114.3(3)	C14	C2	C3	122.5(4)
C40	C39	C33	108.2(3)	C14	C2	C1	129.1(4)
C44	C39	C40	118.9(4)	C14	C13	C15	120.8(4)
C44	C39	C33	132.8(3)	C14	C13	C12	117.0(4)
C88	C87	C86	114.6(3)	C12	C13	C15	122.2(4)
O2	C18	C17	124.6(4)	C71	C72	C73	122.2(4)
O2	C18	C19	116.0(4)	C4	C5	C1	108.4(3)
C17	C18	C19	119.3(4)	C6	C5	C4	122.4(4)
C46	C47	C48	120.1(4)	C6	C5	C1	129.2(4)
C54	C55	C56	114.2(3)	C2	C14	C13	120.6(4)
C47	C46	C45	121.2(4)	C41	C42	C45	119.6(3)
C63	C62	C61	108.7(3)	C43	C42	C45	122.9(4)
C80	C62	C63	122.2(4)	C43	C42	C41	117.5(4)
C80	C62	C61	129.1(4)	C4	C9	C8	119.0(4)
C64	C65	C61	108.1(3)	C39	C44	C43	118.7(4)
C66	C65	C64	122.7(4)	C9	C8	C7	120.6(4)
C66	C65	C61	129.2(4)	O3	C31	C32	127.5(4)
C5	C4	C3	108.6(3)	O3	C31	C40	126.8(4)
C9	C4	C3	131.9(4)	C40	C31	C32	105.7(3)
C9	C4	C5	119.5(4)	C72	C71	C63	118.7(4)
C29	C28	C27	112.9(3)	O5	C61	C62	126.9(4)

C79	C78	C77	120.2(4)	O5	C61	C65	127.8(4)
C62	C63	C64	108.5(3)	C62	C61	C65	105.3(3)
C71	C63	C62	119.0(4)	C11	C12	C13	122.6(4)
C71	C63	C64	132.5(4)	C17	C16	C15	122.5(4)
C37	C36	C38	121.2(3)	C33	C34	C35	118.9(4)
C35	C36	C38	118.5(3)	C19	C20	C15	121.0(4)
C35	C36	C37	120.3(4)	C32	C37	C36	117.7(4)
C77	C76	C75	120.2(4)	O1	C1	C2	126.0(4)
C86	C85	C84	113.9(3)	O1	C1	C5	128.3(4)
C59	C58	C57	112.5(3)	C2	C1	C5	105.7(3)
C76	C75	C74	121.9(4)	C5	C6	C7	117.7(4)
C51	C52	C53	111.5(3)	C50	C49	C48	119.9(4)
C24	C25	C26	113.5(3)	C34	C35	C36	121.1(4)
C78	C79	C74	121.2(4)	C68	C69	C64	119.2(4)
C33	C32	C31	108.1(3)	C49	C50	C45	122.0(4)
C37	C32	C33	122.4(4)	C69	C68	C67	120.5(4)
C37	C32	C31	129.5(3)	C28	C29	C30	114.0(4)
N3	C70	C67	178.0(5)	C58	C59	C60	113.7(4)
O6	C81	C82	108.1(3)	C88	C89	C90	113.8(4)

Table S13: Torsion angles for **CN-FO-Ar**.

A	B	C	D	Angle/°	A	B	C	D	Angle/°
O6	C77	C78	C79	176.9(4)	C17	C18	C19	C20	-3.9(6)
O6	C77	C76	C75	-175.3(4)	C15	C13	C14	C2	-178.5(4)
O4	C48	C47	C46	-175.8(4)	C15	C13	C12	C11	178.0(4)
O4	C48	C49	C50	176.4(4)	C64	C65	C66	C67	-2.0(6)
O2	C18	C17	C16	-175.0(4)	C64	C65	C61	O5	177.0(4)
O2	C18	C19	C20	174.4(4)	C64	C65	C61	C62	-1.3(4)
C77	O6	C81	C82	167.7(3)	C64	C63	C71	C72	179.2(4)
C77	C78	C79	C74	-1.4(6)	C64	C69	C68	C67	-1.0(6)
C77	C76	C75	C74	-1.4(6)	C41	C40	C31	O3	1.0(7)
C74	C73	C80	C62	175.7(3)	C41	C40	C31	C32	-180.0(4)
C74	C73	C72	C71	-174.0(4)	C80	C73	C72	C71	3.0(6)
C48	O4	C51	C52	-169.4(3)	C80	C62	C63	C64	-177.8(4)
C48	C47	C46	C45	-2.7(6)	C80	C62	C63	C71	1.5(6)
C48	C49	C50	C45	1.6(6)	C80	C62	C61	O5	1.1(7)
C73	C74	C75	C76	178.8(4)	C80	C62	C61	C65	179.5(4)
C73	C74	C79	C78	-177.4(4)	C19	C18	C17	C16	3.2(6)
C73	C72	C71	C63	-2.4(6)	C33	C39	C40	C41	-179.3(4)
C23	C24	C25	C26	179.7(3)	C33	C39	C40	C31	-0.8(4)
C23	C22	C21	O2	178.1(3)	C33	C39	C44	C43	178.3(4)
C54	C53	C52	C51	178.0(3)	C33	C32	C31	O3	177.1(4)
C24	C23	C22	C21	-179.8(3)	C33	C32	C31	C40	-2.0(4)
C53	C54	C55	C56	179.2(3)	C33	C32	C37	C36	-0.2(6)

C53	C52	C51	O4	179.7(3)	C33	C34	C35	C36	-0.2(6)
C56	C57	C58	C59	-178.1(3)	C10	C7	C8	C9	-179.5(4)
C26	C27	C28	C29	179.0(3)	C10	C7	C6	C5	177.7(4)
C3	C4	C5	C1	-1.5(5)	C21	O2	C18	C17	12.2(6)
C3	C4	C5	C6	179.5(4)	C21	O2	C18	C19	-166.1(4)
C3	C4	C9	C8	178.3(4)	C66	C65	C64	C63	179.7(4)
C3	C11	C12	C13	-0.8(6)	C66	C65	C64	C69	3.0(6)
C3	C2	C14	C13	1.7(6)	C66	C65	C61	O5	0.5(7)
C3	C2	C1	O1	-179.6(4)	C66	C65	C61	C62	-177.9(4)
C3	C2	C1	C5	1.1(5)	C66	C67	C68	C69	2.0(7)
C83	C82	C81	O6	176.7(3)	C51	O4	C48	C47	172.2(3)
C83	C84	C85	C86	179.6(3)	C51	O4	C48	C49	-8.2(6)
C57	C56	C55	C54	-177.9(3)	C11	C3	C4	C5	-175.2(4)
C57	C58	C59	C60	179.7(4)	C11	C3	C4	C9	6.4(8)
C82	C83	C84	C85	179.1(3)	C11	C3	C2	C14	-3.7(6)
C22	C23	C24	C25	-179.3(3)	C11	C3	C2	C1	175.8(4)
C38	C36	C37	C32	177.1(4)	C2	C3	C4	C5	2.2(4)
C38	C36	C35	C34	-176.9(4)	C2	C3	C4	C9	-176.2(4)
C84	C83	C82	C81	177.2(3)	C2	C3	C11	C12	3.2(6)
C27	C26	C25	C24	179.4(3)	C13	C15	C16	C17	176.3(4)
C27	C28	C29	C30	179.8(4)	C13	C15	C20	C19	-177.0(4)
C39	C40	C41	C42	1.3(6)	C72	C73	C80	C62	-1.3(6)
C39	C40	C31	O3	-177.4(4)	C5	C4	C9	C8	0.0(6)
C39	C40	C31	C32	1.7(4)	C14	C2	C1	O1	0.0(8)
C39	C33	C34	C35	-179.4(4)	C14	C2	C1	C5	-179.4(4)
C87	C86	C85	C84	178.6(3)	C14	C13	C12	C11	-1.2(6)
C87	C88	C89	C90	179.9(4)	C42	C45	C50	C49	-177.9(4)
C18	O2	C21	C22	164.7(3)	C42	C43	C44	C39	-0.7(6)
C18	C17	C16	C15	0.0(6)	C9	C4	C5	C1	177.1(4)
C18	C19	C20	C15	1.3(6)	C9	C4	C5	C6	-1.8(6)
C47	C48	C49	C50	-4.0(6)	C44	C39	C40	C41	-1.5(6)
C47	C46	C45	C42	178.4(4)	C44	C39	C40	C31	177.0(4)
C47	C46	C45	C50	0.2(6)	C44	C39	C33	C32	-177.8(4)
C55	C54	C53	C52	-179.4(3)	C44	C39	C33	C34	-0.1(7)
C55	C56	C57	C58	177.5(3)	C44	C43	C42	C45	177.4(4)
C46	C45	C42	C41	-150.2(4)	C44	C43	C42	C41	0.5(6)
C46	C45	C42	C43	33.0(6)	C8	C7	C6	C5	-0.6(6)
C46	C45	C50	C49	0.3(6)	C31	C32	C33	C39	1.5(4)
C62	C63	C64	C65	-3.4(5)	C31	C32	C33	C34	-176.5(4)
C62	C63	C64	C69	172.8(4)	C31	C32	C37	C36	178.0(4)
C62	C63	C71	C72	0.1(6)	C31	C40	C41	C42	-176.8(4)
C65	C64	C69	C68	-1.4(6)	C71	C63	C64	C65	177.4(4)
C4	C3	C11	C12	-179.6(4)	C71	C63	C64	C69	-6.4(8)
C4	C3	C2	C14	178.4(4)	C61	C62	C63	C64	2.5(4)

C4	C3	C2	C1	-2.0(4)	C61	C62	C63	C71	-178.2(4)
C4	C5	C1	O1	-179.0(5)	C61	C62	C80	C73	178.8(4)
C4	C5	C1	C2	0.3(5)	C61	C65	C64	C63	2.9(5)
C4	C5	C6	C7	2.1(6)	C61	C65	C64	C69	-173.8(4)
C4	C9	C8	C7	1.4(6)	C61	C65	C66	C67	174.1(4)
C78	C77	C76	C75	2.4(6)	C12	C13	C14	C2	0.8(6)
C63	C62	C80	C73	-0.9(6)	C16	C15	C13	C14	-16.8(6)
C63	C62	C61	O5	-179.2(4)	C16	C15	C13	C12	164.0(4)
C63	C62	C61	C65	-0.8(4)	C16	C15	C20	C19	1.8(6)
C63	C64	C69	C68	-177.3(4)	C20	C15	C13	C14	162.0(4)
C76	C77	C78	C79	-1.0(6)	C20	C15	C13	C12	-17.2(6)
C85	C86	C87	C88	-178.3(3)	C20	C15	C16	C17	-2.5(6)
C75	C74	C73	C80	-39.0(5)	C37	C36	C35	C34	2.1(6)
C75	C74	C73	C72	137.9(4)	C37	C32	C33	C39	-179.9(4)
C75	C74	C79	C78	2.4(6)	C37	C32	C33	C34	2.1(6)
C25	C26	C27	C28	-178.9(3)	C37	C32	C31	O3	-1.4(7)
C79	C74	C73	C80	140.7(4)	C37	C32	C31	C40	179.6(4)
C79	C74	C73	C72	-42.4(6)	C1	C2	C14	C13	-177.8(4)
C79	C74	C75	C76	-0.9(6)	C1	C5	C6	C7	-176.7(4)
C32	C33	C34	C35	-1.8(6)	C6	C7	C8	C9	-1.1(7)
C70	C67	C66	C65	-178.2(4)	C6	C5	C1	O1	-0.2(8)
C70	C67	C68	C69	179.8(4)	C6	C5	C1	C2	179.2(4)
C81	O6	C77	C78	-179.4(3)	C49	C48	C47	C46	4.6(6)
C81	O6	C77	C76	-1.7(5)	C35	C36	C37	C32	-1.8(6)
C40	C39	C33	C32	-0.5(4)	C50	C45	C42	C41	27.9(6)
C40	C39	C33	C34	177.3(4)	C50	C45	C42	C43	-148.9(4)
C40	C39	C44	C43	1.2(6)	C68	C67	C66	C65	-0.5(6)
C40	C41	C42	C45	-177.8(4)	C89	C88	C87	C86	178.3(3)
C40	C41	C42	C43	-0.8(6)					

Table S14: Hydrogen Atom Coordinates (Å×10^4) and Isotropic Displacement Parameters (Å^2×10^3) for **CN-FO-Ar**.

**Atom**	***x***	***y***	***z***	**U(eq)**
H86A	9157.31	10031.42	5260.02	31
H86B	9026.15	8180.74	5553.21	31
H23A	8357.37	1845.7	7629.93	31
H23B	8224.78	-17.23	7912.72	31
H54A	8782.1	4912.31	5956.2	32
H54B	8641	6771.16	6218.18	32
H24A	8646.1	-1685.04	7902.92	30
H24B	8778.18	190.39	7628.97	30
H53A	8368.96	3165.71	5981.4	32
H53B	8228.15	5034.3	6236.67	32
H56A	9152.98	5023.49	6920.59	30

H56B	9014.84	6857.5	7206.26	30
H26A	9017.52	-1714.85	8864.88	31
H26B	9151.3	143.66	8583.66	31
H83A	8360.68	11654.83	4311.62	31
H83B	8232.6	9751.73	4578.08	31
H88A	9405.51	8339.63	6536.1	33
H88B	9529.32	10187.98	6224	33
H57A	9101.2	3306.02	7882.97	33
H57B	8975.39	5186.77	8168.65	33
H82A	8408.2	10103.4	3327.86	31
H82B	8294.2	8146.98	3594.64	31
H22A	8408.81	140.18	6665.71	32
H22B	8275.07	-1719.53	6948.57	32
H84A	8656.58	8150.58	4572.92	31
H84B	8785.13	10036.41	4296.11	31
H27A	8976.13	-58.95	9834.21	33
H27B	9104.6	1818.86	9553.2	33
H87A	9100.66	11769.11	6214.83	32
H87B	8978.02	9889.1	6508.08	32
H47	7712.77	6185	3280.32	35
H55A	8735.92	3292.59	6936.45	32
H55B	8598.86	5165.71	7198.16	32
H46	7263.72	5920.93	2797.33	35
H28A	9398.2	-1682.54	9835.01	33
H28B	9527.33	176.85	9541.8	33
H78	7751.6	8721.04	1573.06	35
H76	7485.12	10699.24	3203.27	37
H85A	8735.06	11686.77	5265.17	32
H85B	8604.74	9808.55	5541.83	32
H58A	9526.66	4938.56	7873.45	35
H58B	9399.7	6783.94	8180.88	35
H75	7037.37	10523.76	2709.77	36
H52A	8261.71	6613.38	5250.68	33
H52B	8412.4	4783.09	4994.88	33
H25A	8599.38	-38.98	8875.19	32
H25B	8732.9	1831.77	8601.13	32
H79	7302.86	8670.14	1066.13	36
H81A	7861.4	9569.43	3634.28	34
H81B	7974.15	11586.46	3406.47	34
H17	7505.98	1543.01	6367.81	38
H41	6677.41	3664.44	4057.28	33
H80	6676.98	8344.16	2333.99	35
H19	7747.91	-1929.24	4994.68	37
H21A	7854.46	-80.82	6943.11	33

H21B	7987.83	1821.29	6680.86	33
H43	6886.14	4156.32	2248.81	35
H66	5534.76	7690.25	1818.84	36
H51A	8009.65	2922.63	5027.39	35
H51B	7858.45	4752.1	5287.92	35
H11	6439.47	-2184.94	3554.88	34
H72	6880.36	10448.04	647.67	37
H14	6665	249.78	5650.6	36
H9	5871.87	-2660.33	3363.26	36
H44	6430.61	3718.49	1809.15	35
H8	5399.06	-2598.6	3413.74	38
H71	6420.22	10608.15	224.1	37
H12	6896.12	-1778.51	3984.11	37
H16	7057.76	1303.38	5875.76	38
H34	5866.99	3221.76	1588.85	36
H20	7298.97	-2218.64	4513.96	36
H37	5545.49	2579.32	3615.18	35
H6	5524.8	86.97	5162.71	40
H49	7532.36	2836.29	4764.52	37
H35	5400.42	2771.22	1650.07	37
H69	5856.34	10687.12	27.86	40
H50	7085.96	2537.07	4271.73	36
H68	5386.88	10350.88	61.52	41
H29A	9346.29	51.18	10787.32	44
H29B	9476.51	1904.41	10494.43	44
H59A	9472.1	3147.56	8816.4	45
H59B	9346.99	4997.53	9123.68	45
H89A	9345.32	10148.49	7465.51	44
H89B	9469.69	11993.29	7153.69	44
H90A	9900.48	10487.8	7213.46	71
H90B	9777.66	8591.62	7499	71
H90C	9801.92	10526.36	7913.87	71
H30A	9901.04	287.9	10523.75	72
H30B	9770.68	-1568.41	10816.59	72
H30C	9807.48	368.52	11228.84	72
H60A	9901.74	4644.43	8867.9	73
H60B	9803.75	4688.55	9568.86	73
H60C	9779.18	6575.78	9129.96	73

6. NMR spectra

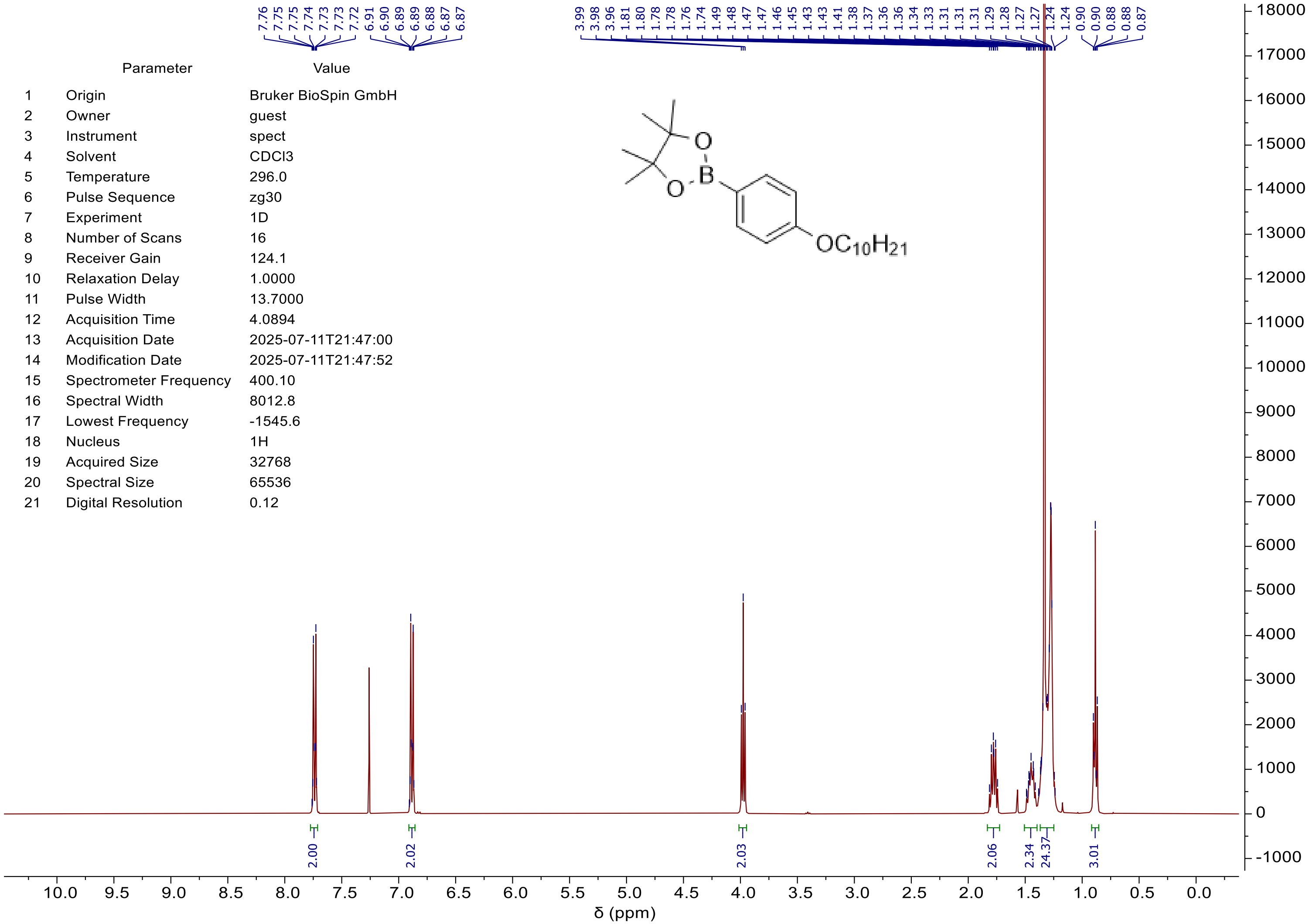

Figure S48: ^{1}H NMR (400 MHz, $CDCl_3$) of **Bpin-Ar**.

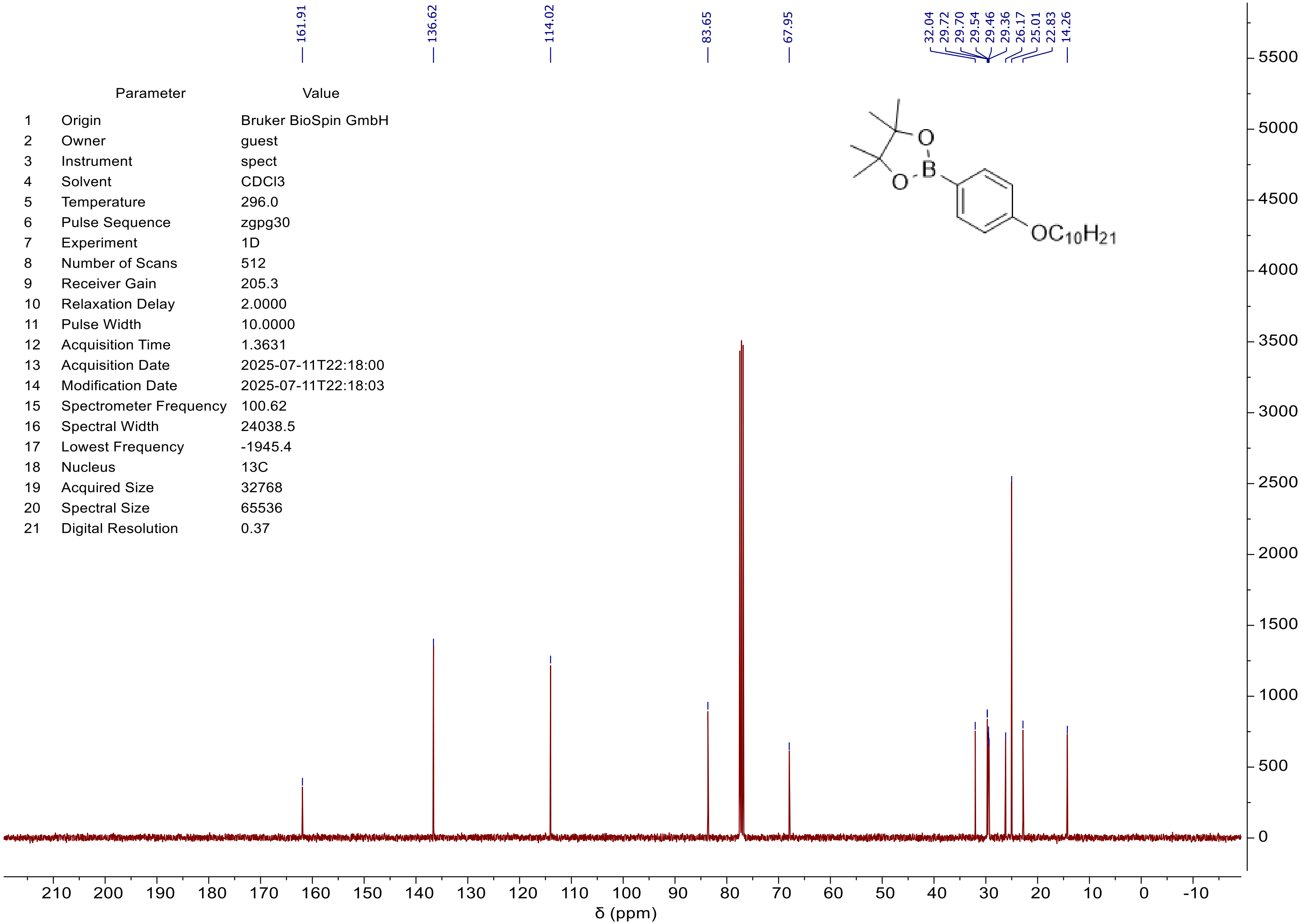

Figure S49: ^{13}C NMR (101 MHz, $CDCl_3$) of **Bpin-Ar**.

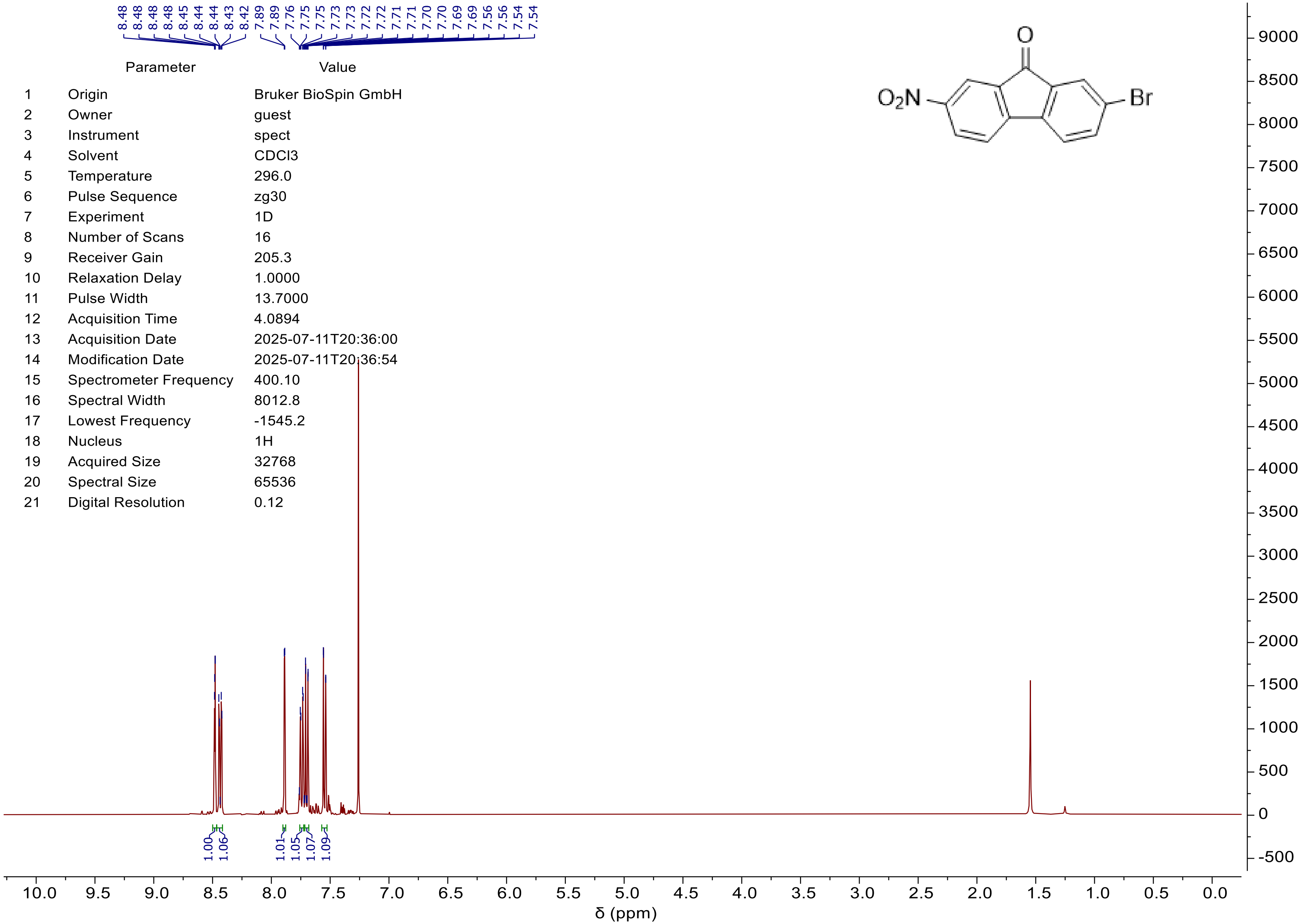

Figure S50: ^{1}H NMR (400 MHz, $CDCl_3$) of **NO_2-FO-Br**.

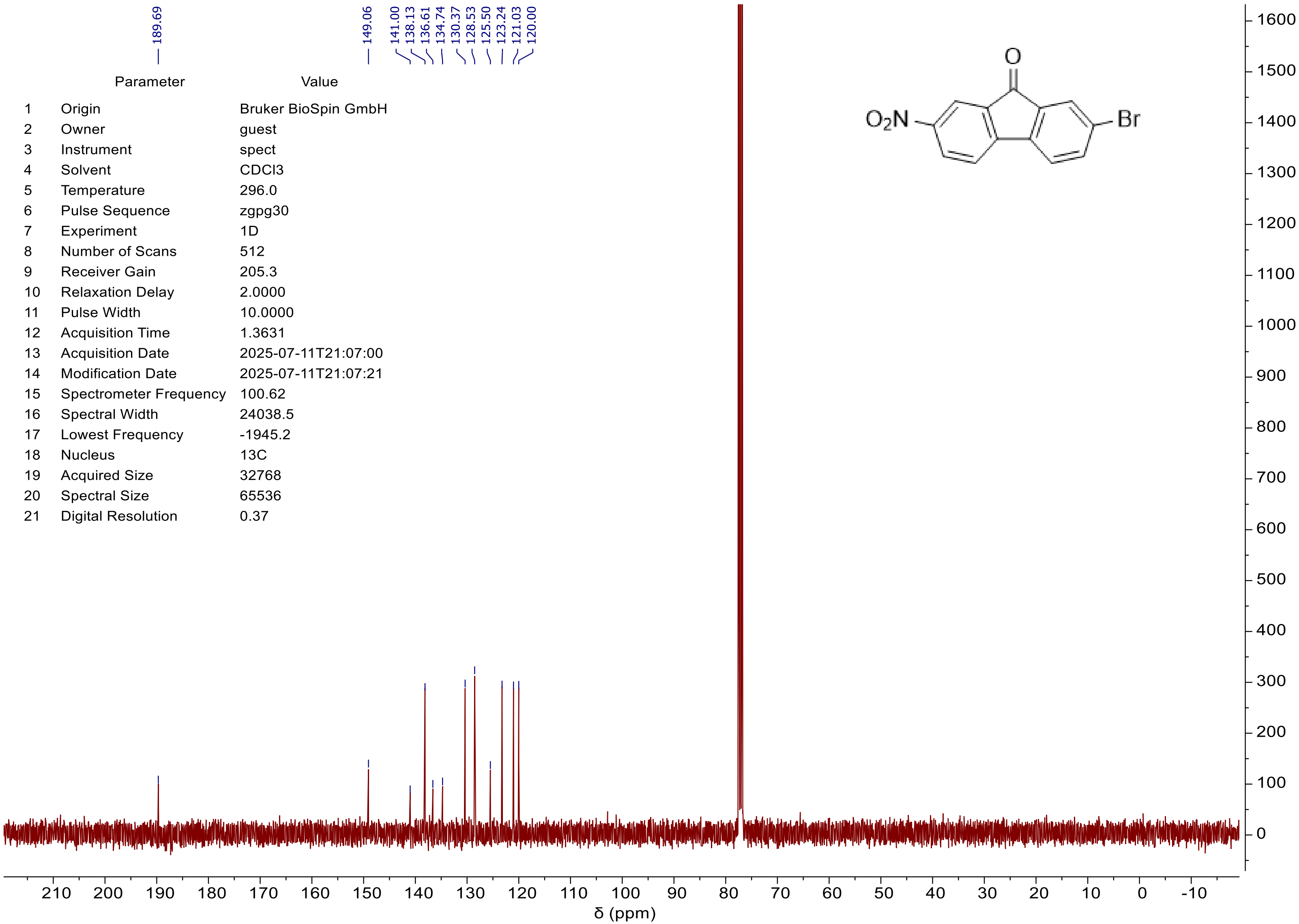

Figure S51: ^{13}C NMR (101 MHz, $CDCl_3$) of **NO_2-FO-Br**.

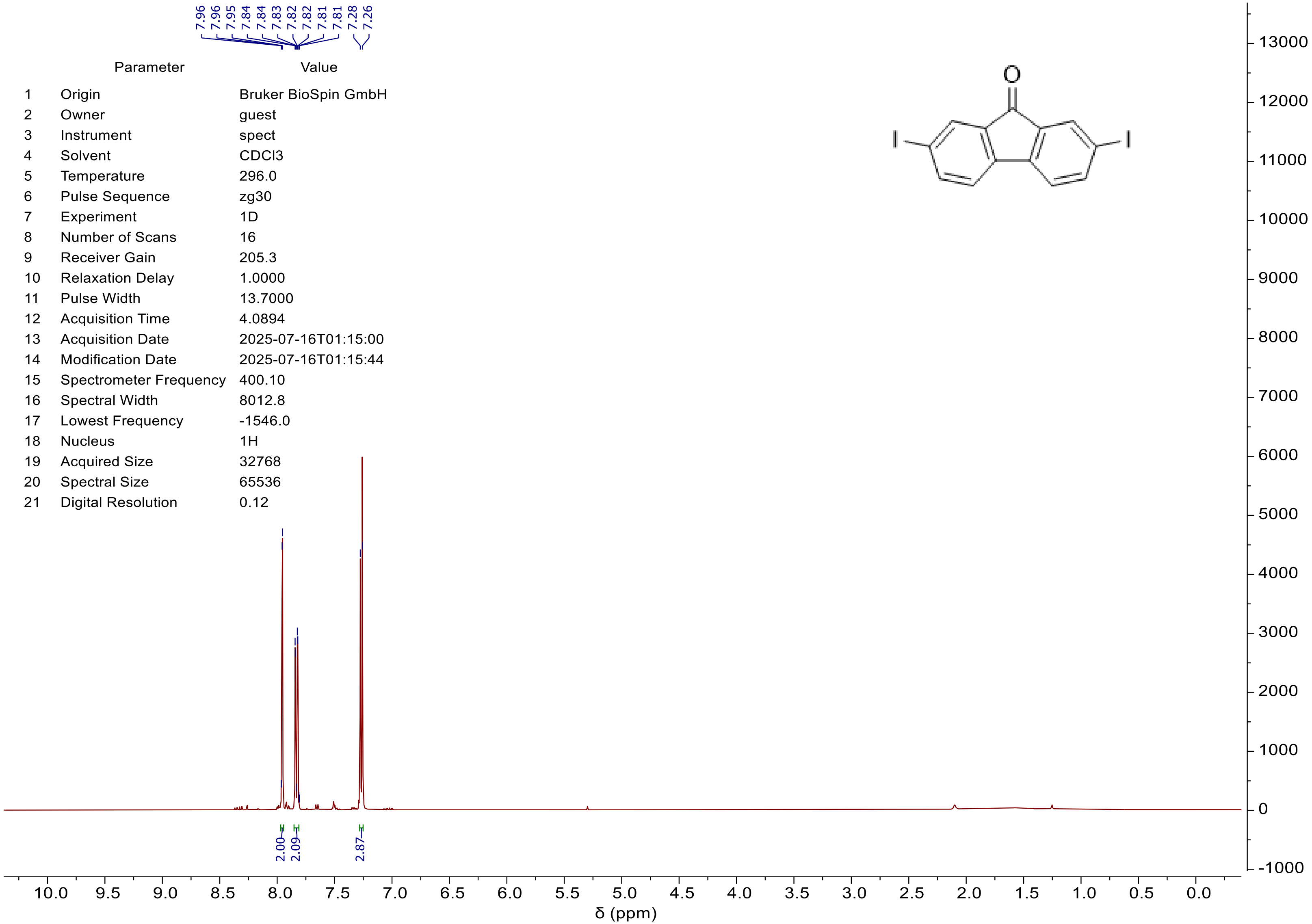

Figure S52: ^{1}H NMR (400 MHz, $CDCl_3$) of **I-FO-I**.

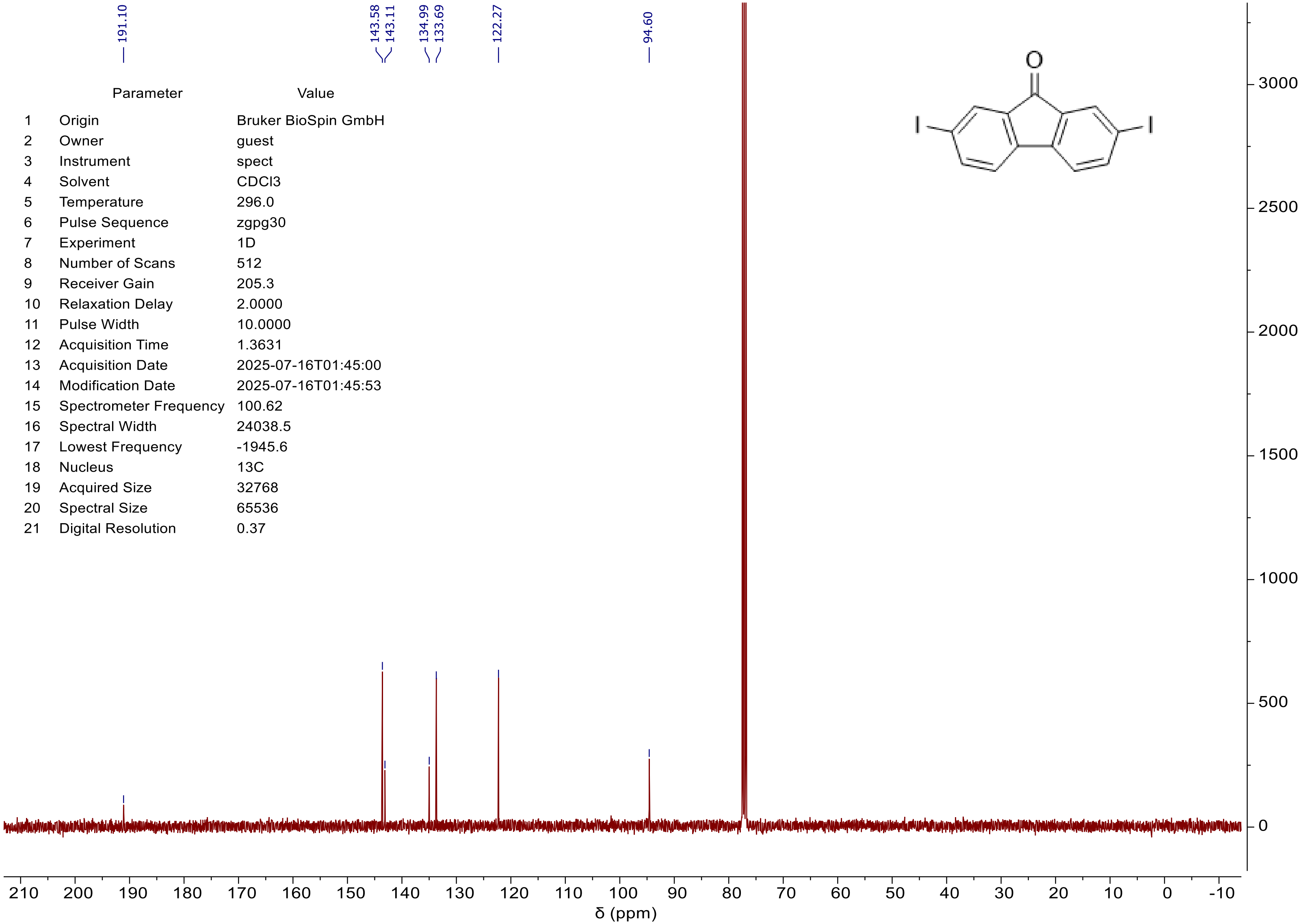

	Parameter	Value
1	Origin	Bruker BioSpin GmbH
2	Owner	guest
3	Instrument	spect
4	Solvent	CDCl3
5	Temperature	296.0
6	Pulse Sequence	zgpg30
7	Experiment	1D
8	Number of Scans	512
9	Receiver Gain	205.3
10	Relaxation Delay	2.0000
11	Pulse Width	10.0000
12	Acquisition Time	1.3631
13	Acquisition Date	2025-07-16T01:45:00
14	Modification Date	2025-07-16T01:45:53
15	Spectrometer Frequency	100.62
16	Spectral Width	24038.5
17	Lowest Frequency	-1945.6
18	Nucleus	13C
19	Acquired Size	32768
20	Spectral Size	65536
21	Digital Resolution	0.37

Figure S53: ^{13}C NMR (101 MHz, $CDCl_3$) of **I-FO-I**.

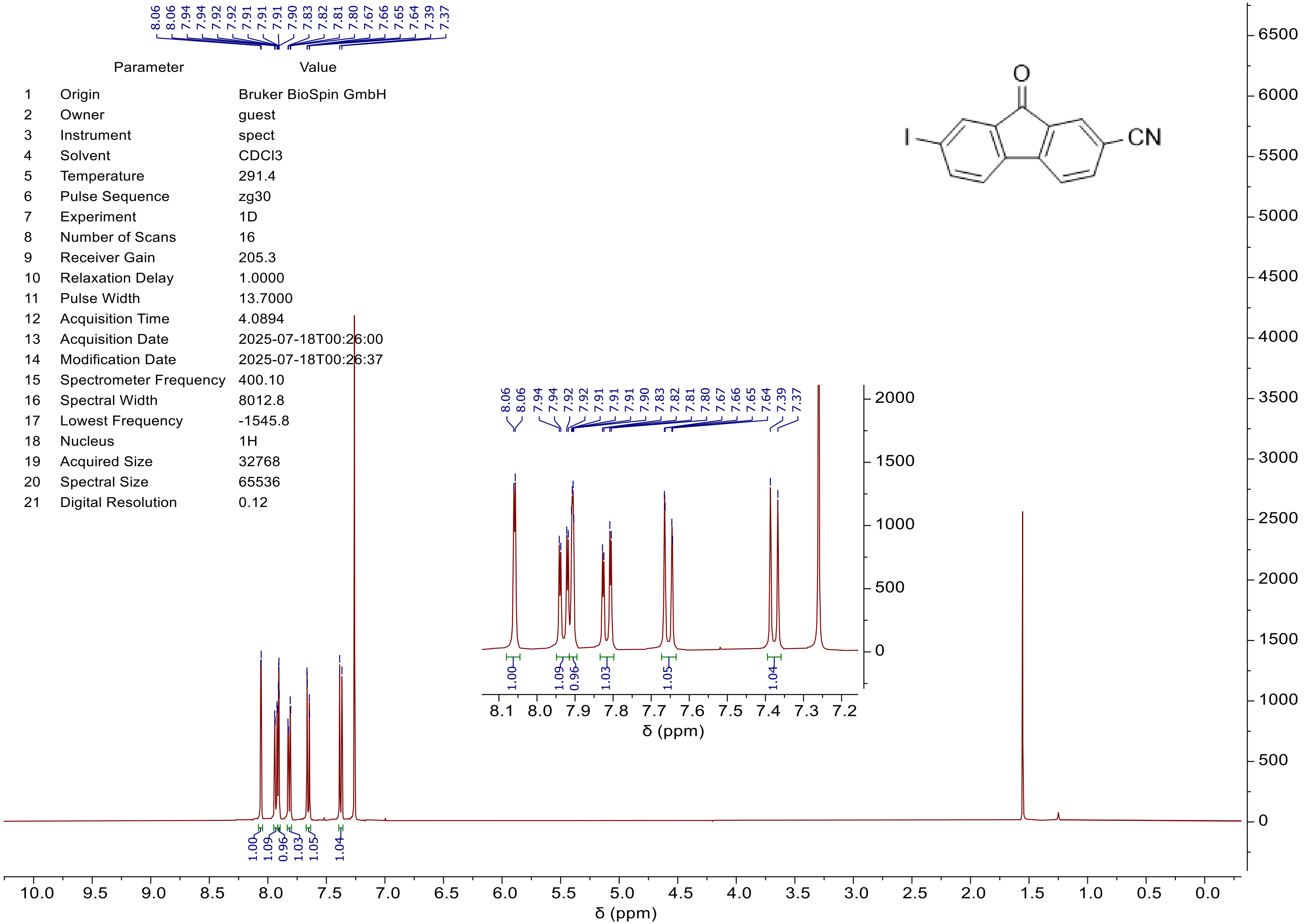

Figure S54: ^{1}H NMR (400 MHz, $CDCl_3$) of **CN-FO-I**.

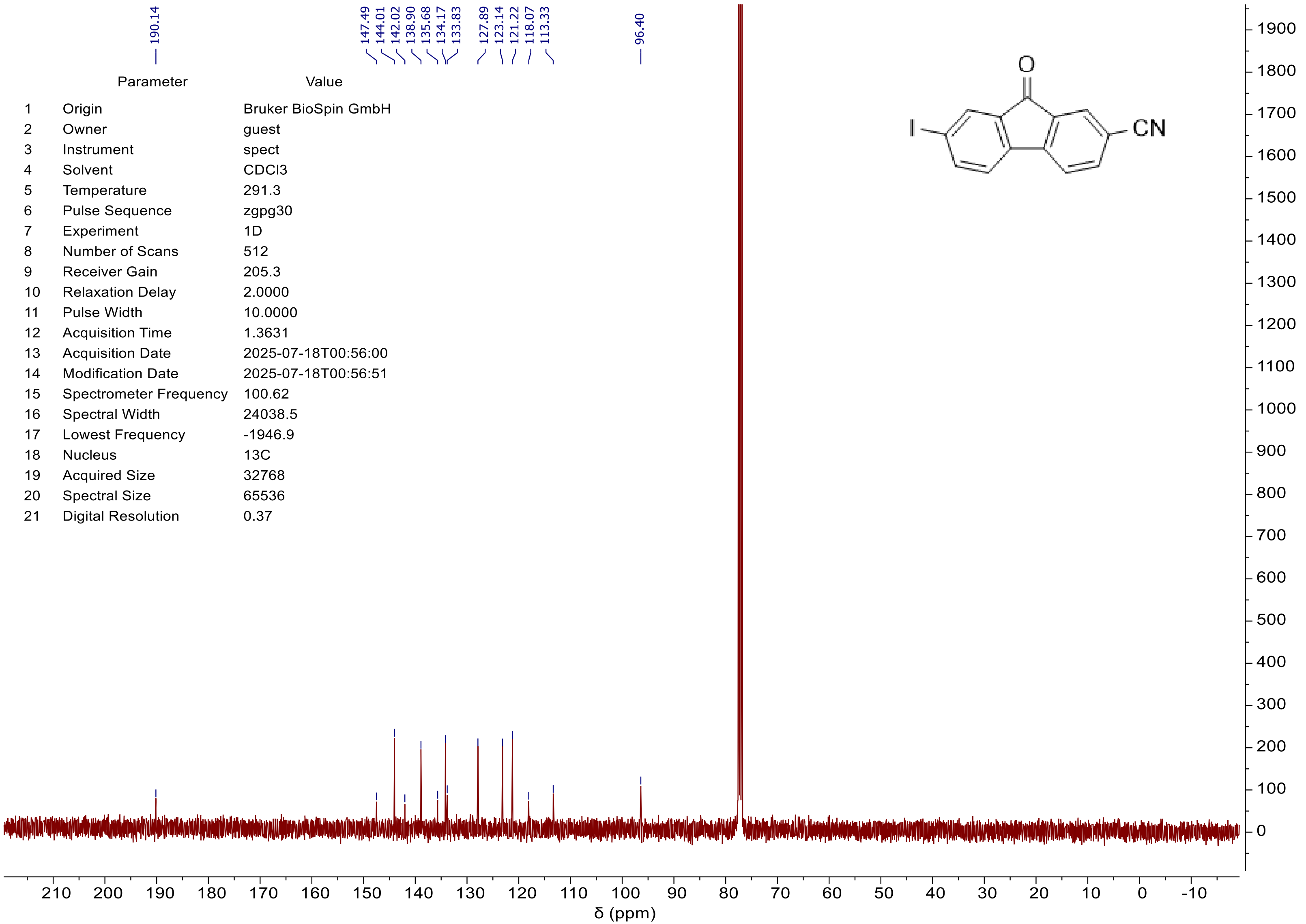

Figure S55: ^{13}C NMR (101 MHz, $CDCl_3$) of **CN-FO-I**.

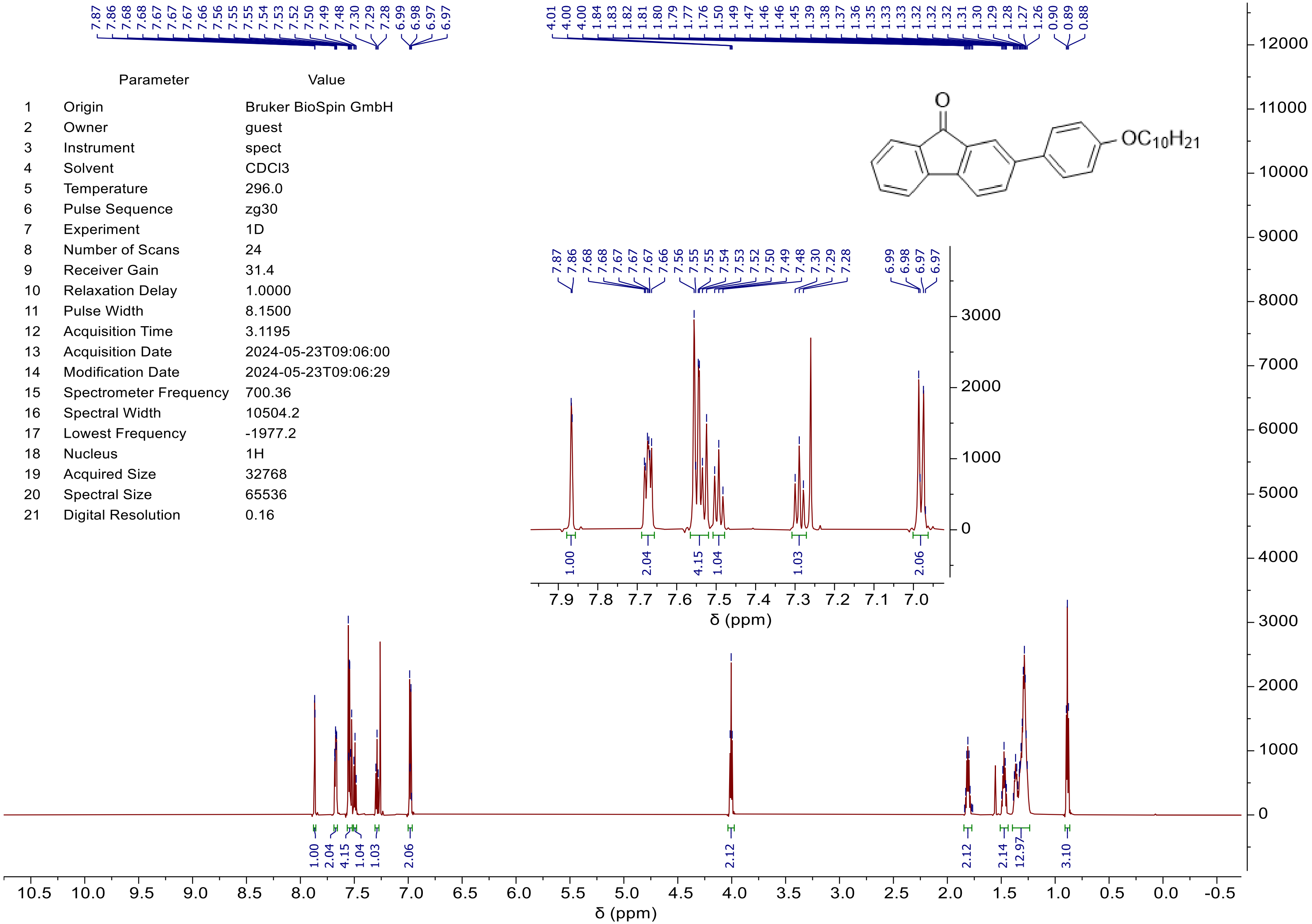

Figure S56: ^{1}H NMR (700 MHz, $CDCl_3$) of **H-FO-Ar**.

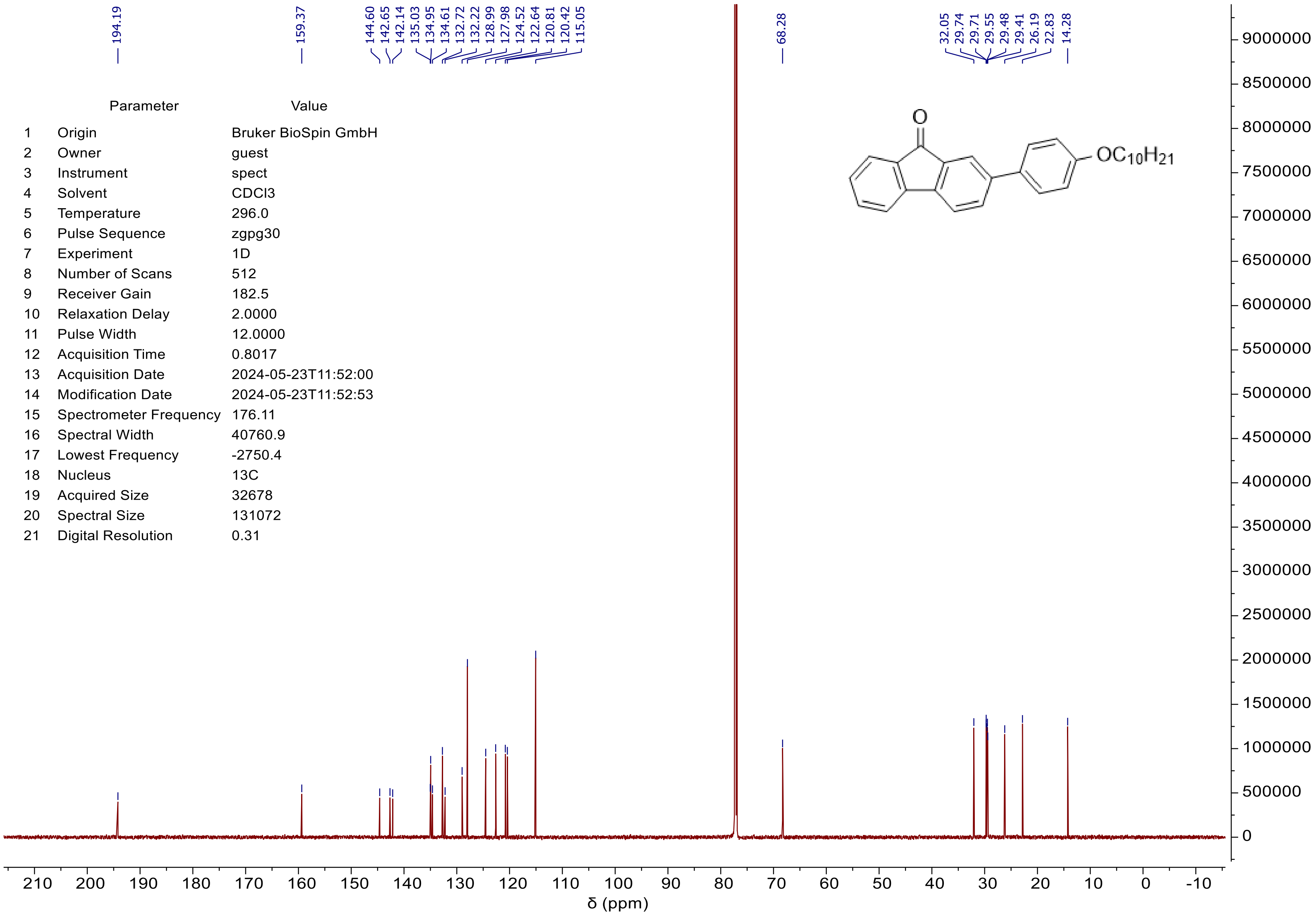

Figure S57: ^{13}C NMR (176 MHz, $CDCl_3$) of **H-FO-Ar**.

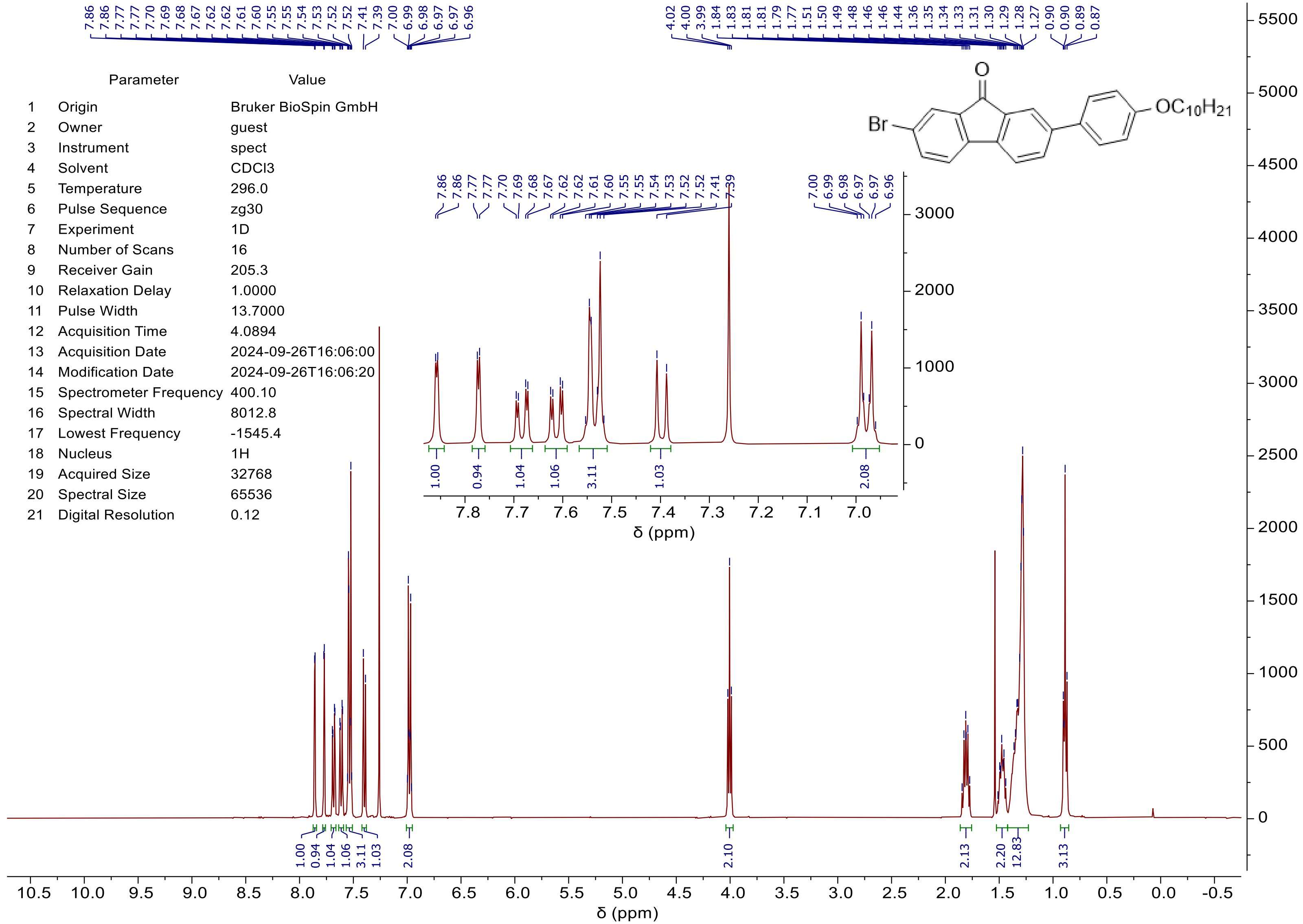

Figure S58: ^{1}H NMR (400 MHz, $CDCl_3$) of **Br-FO-Ar**.

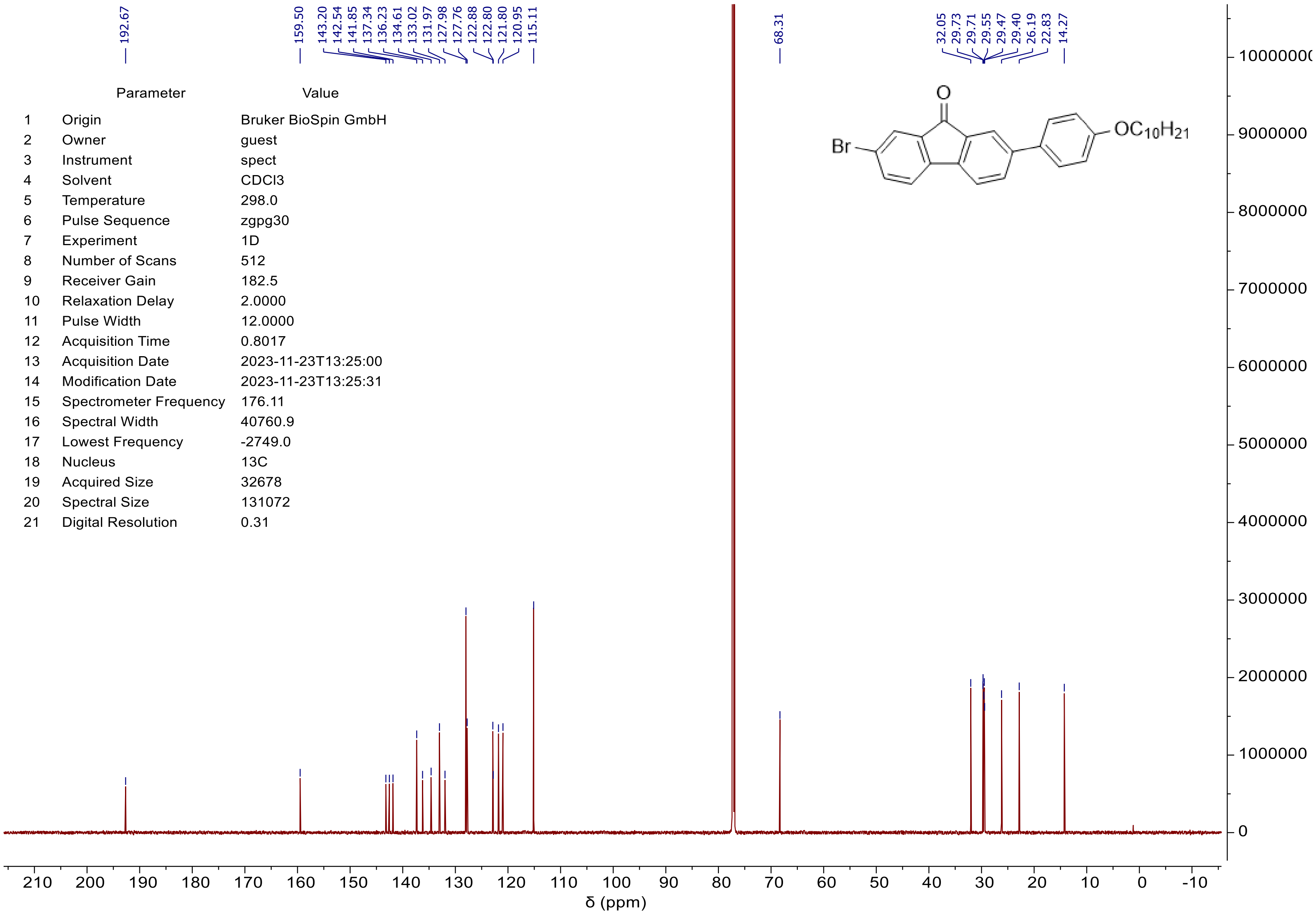

Figure S59: ^{13}C NMR (176 MHz, $CDCl_3$) of **Br-FO-Ar**.

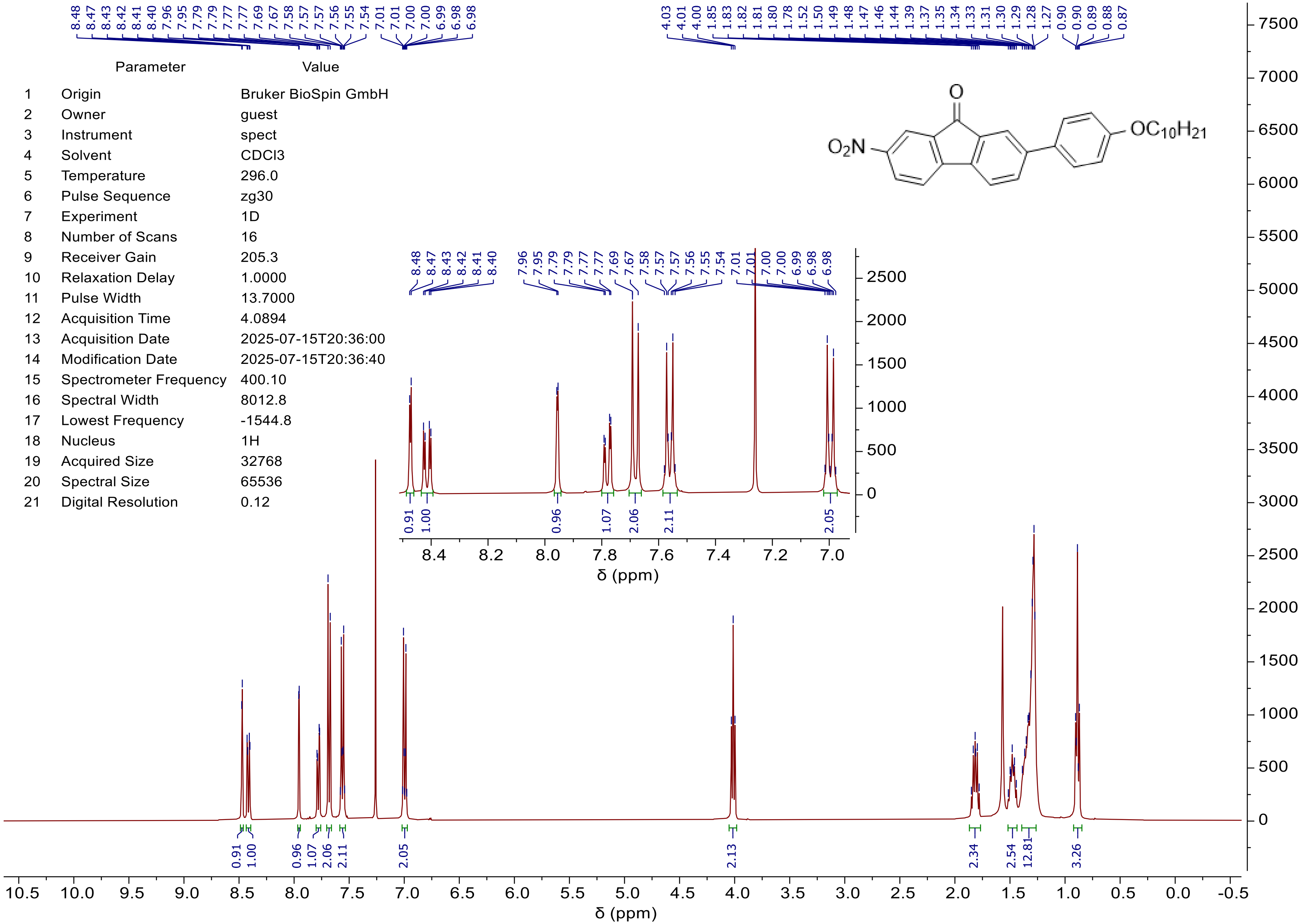

Figure S60: ^{1}H NMR (400 MHz, $CDCl_3$) of **NO_2-FO-Ar**.

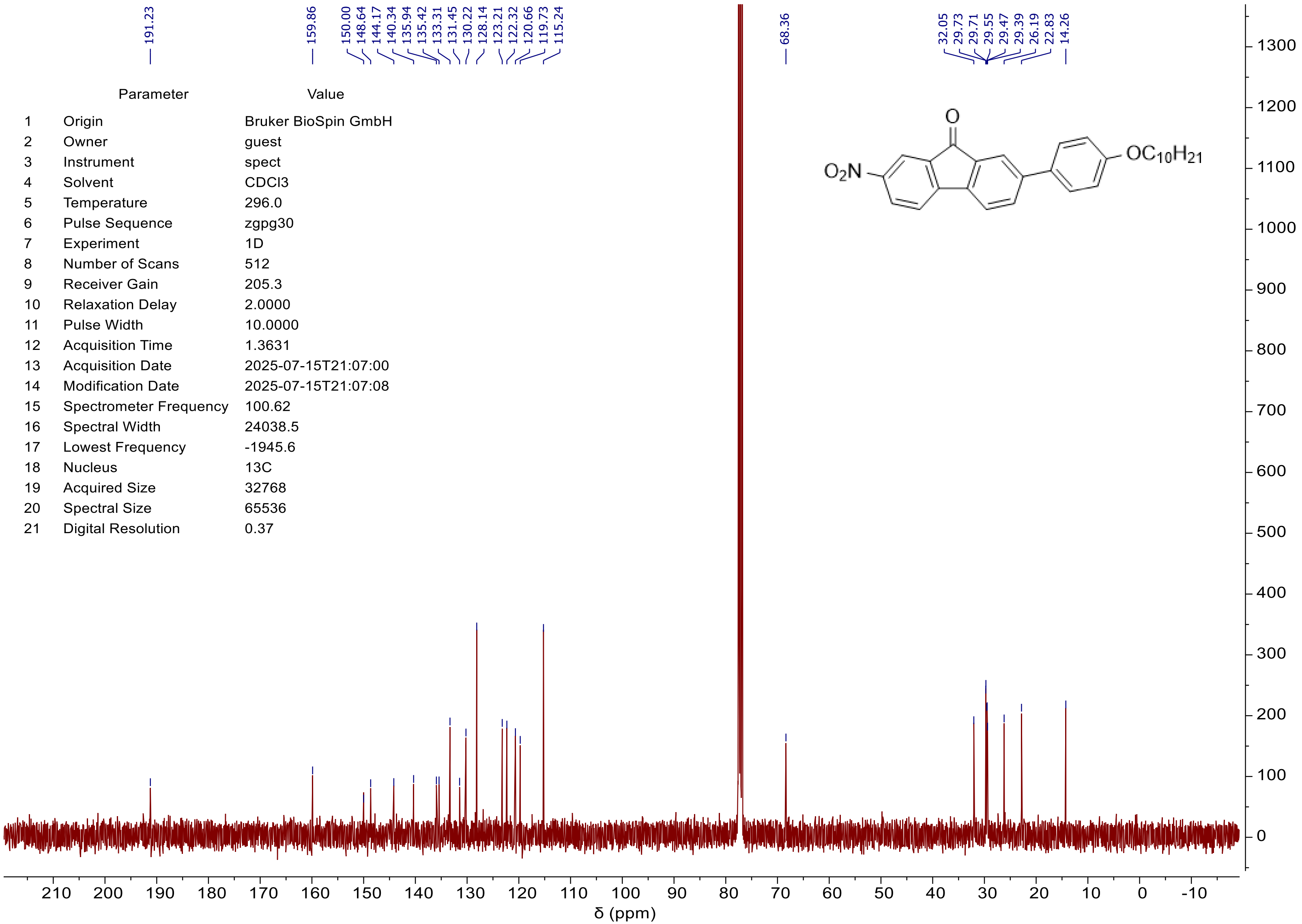

Figure S61: ^{13}C NMR (101 MHz, $CDCl_3$) of **NO_2-FO-Ar**.

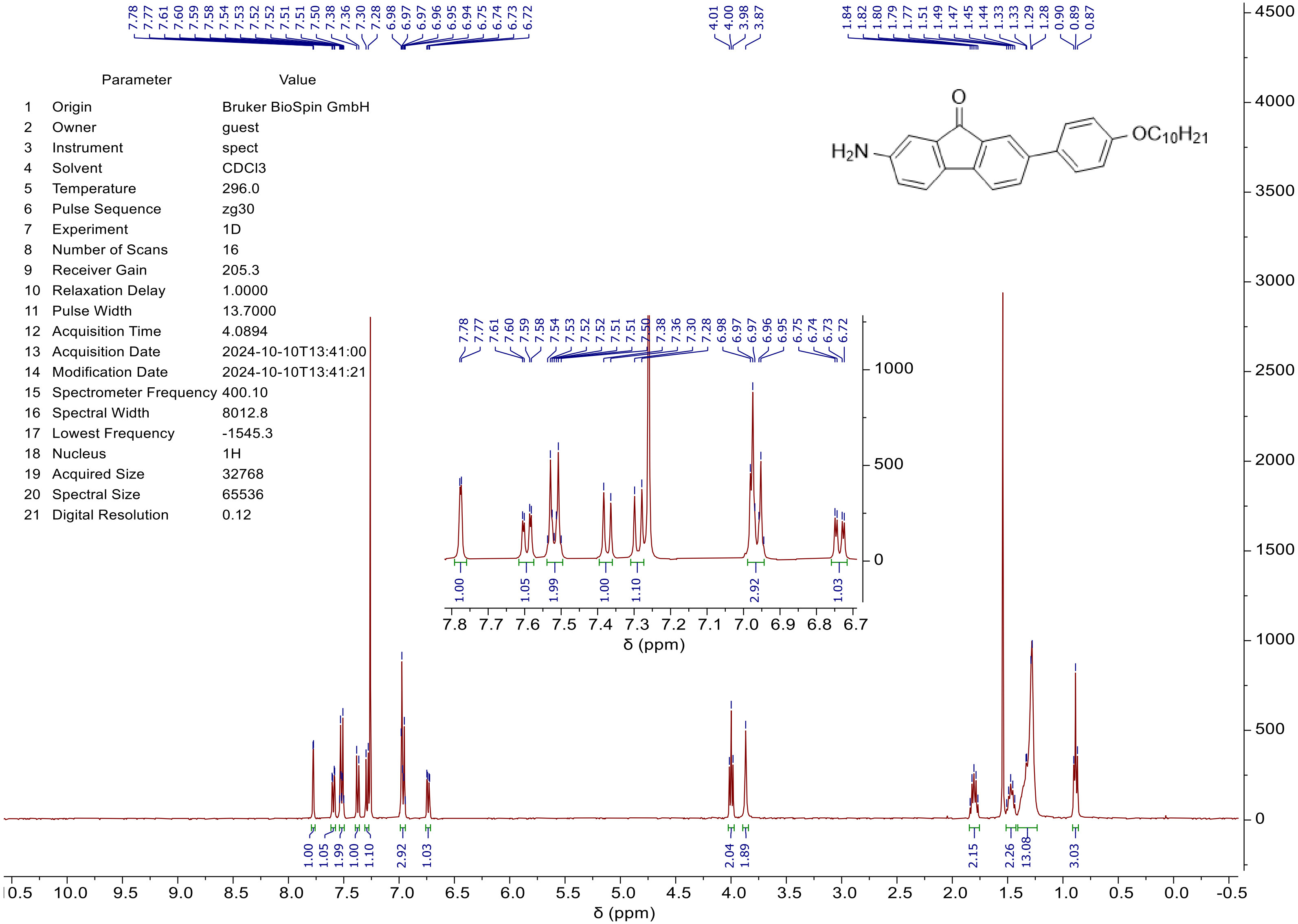

Figure S62: ^{1}H NMR (400 MHz, $CDCl_3$) of **NH_2-FO-Ar**.

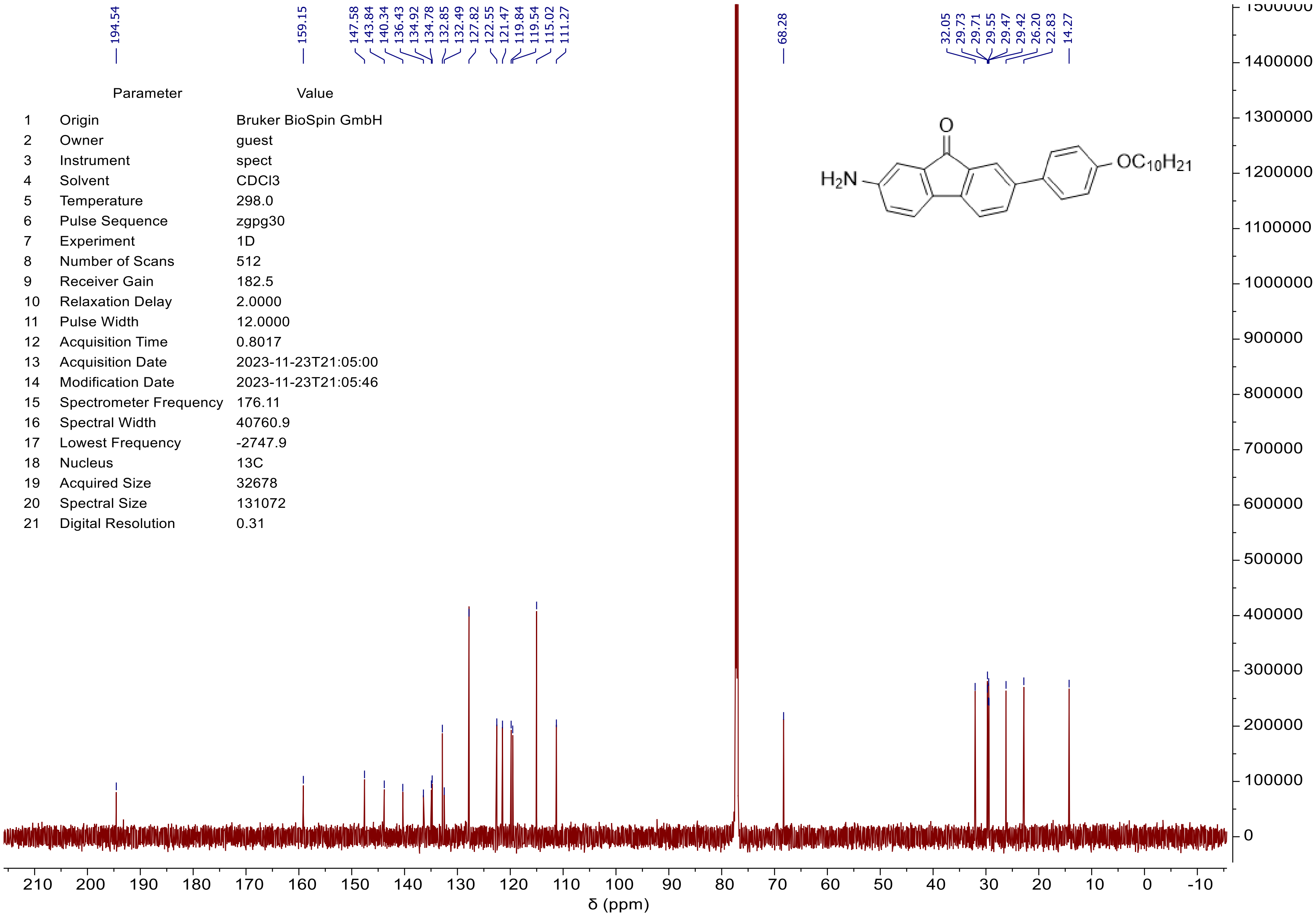

Figure S63: ^{13}C NMR (176 MHz, $CDCl_3$) of **NH_2-FO-Ar**.

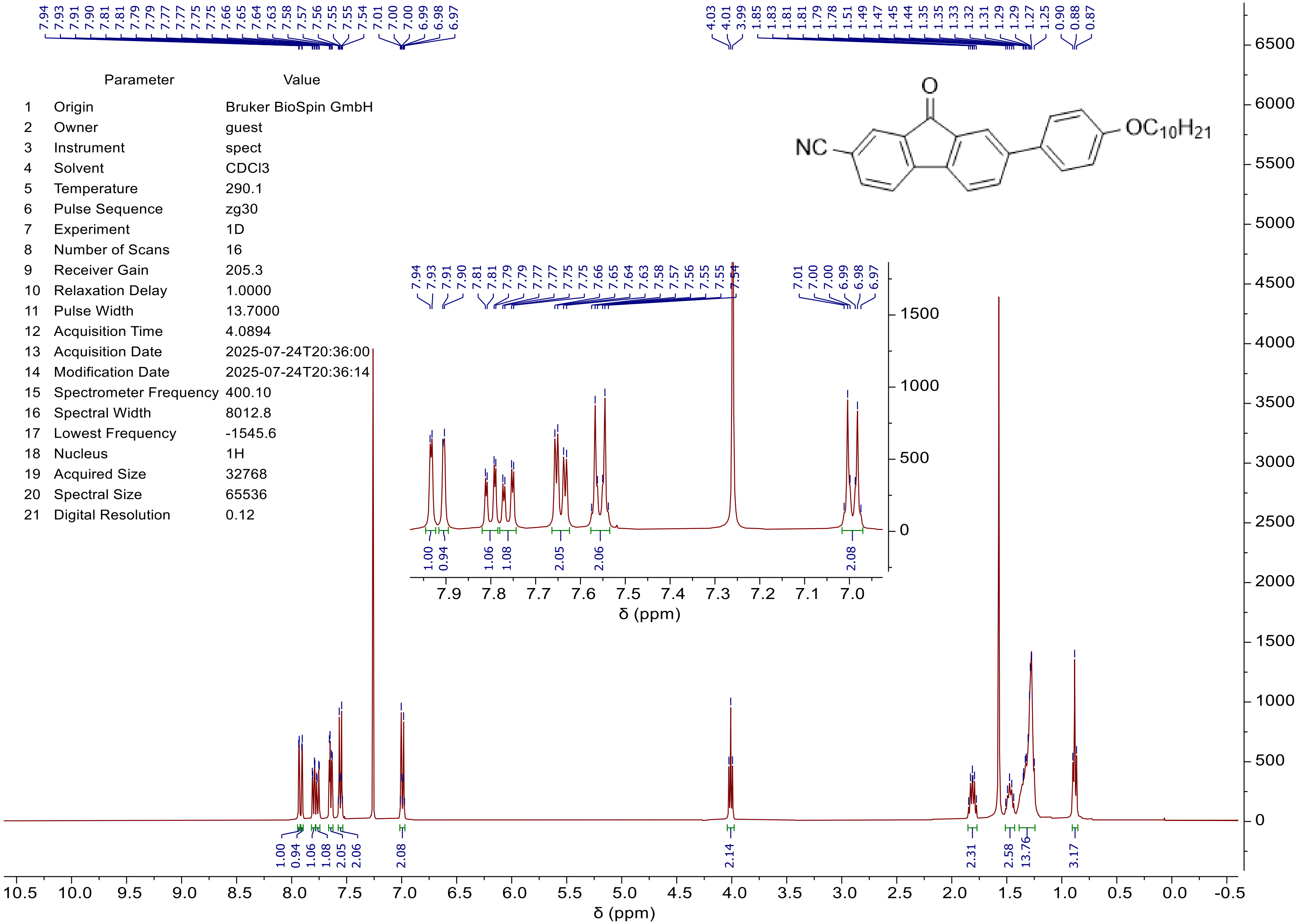

Figure S64: ^{1}H NMR (400 MHz, $CDCl_3$) of **CN-FO-Ar**.

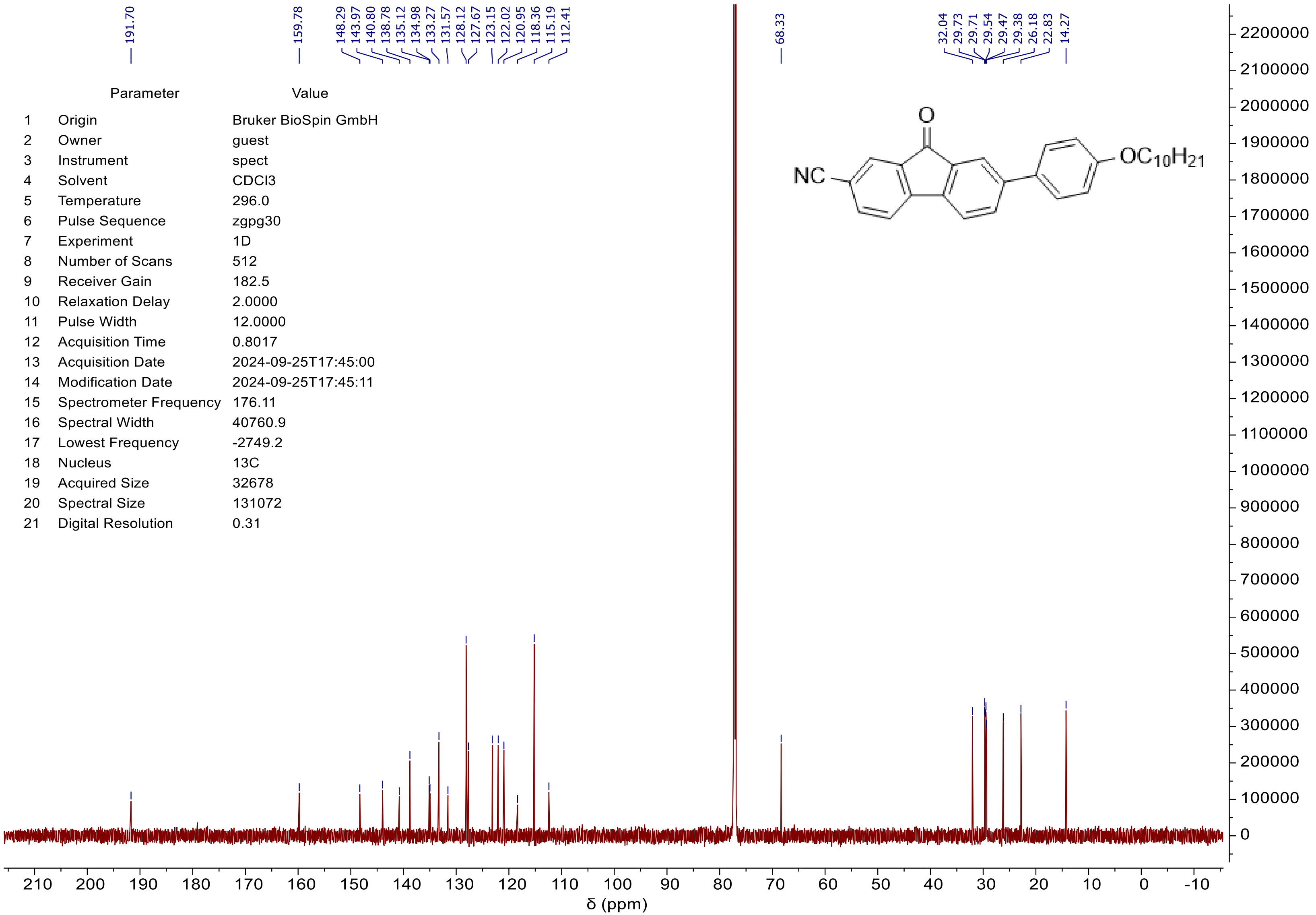

Figure S65: ^{13}C NMR (176 MHz, $CDCl_3$) of **CN-FO-Ar**.

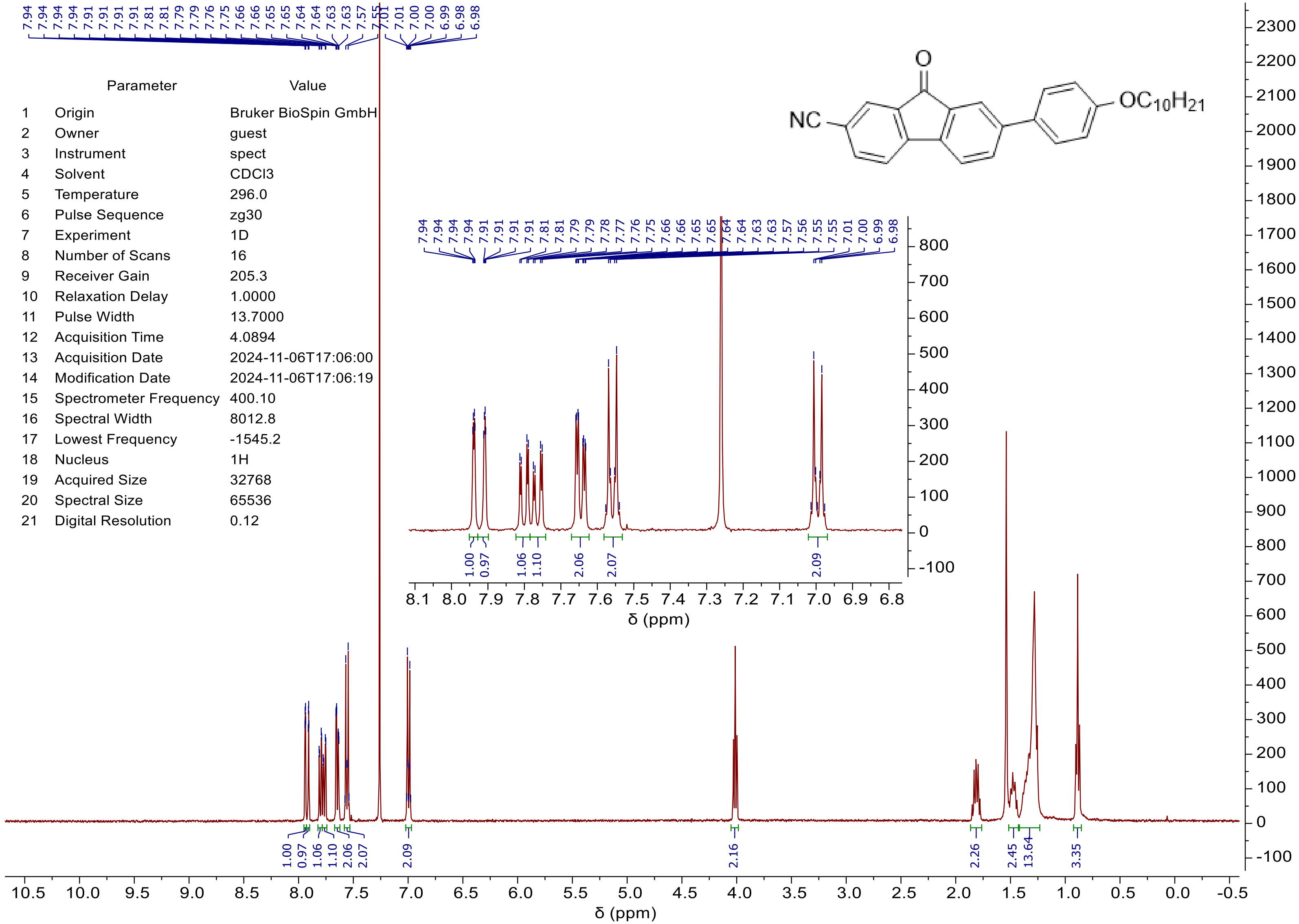

Figure S66: ^{1}H NMR (400 MHz, $CDCl_3$) of **CN-FO-Ar^h^**.

7. Mass spectra

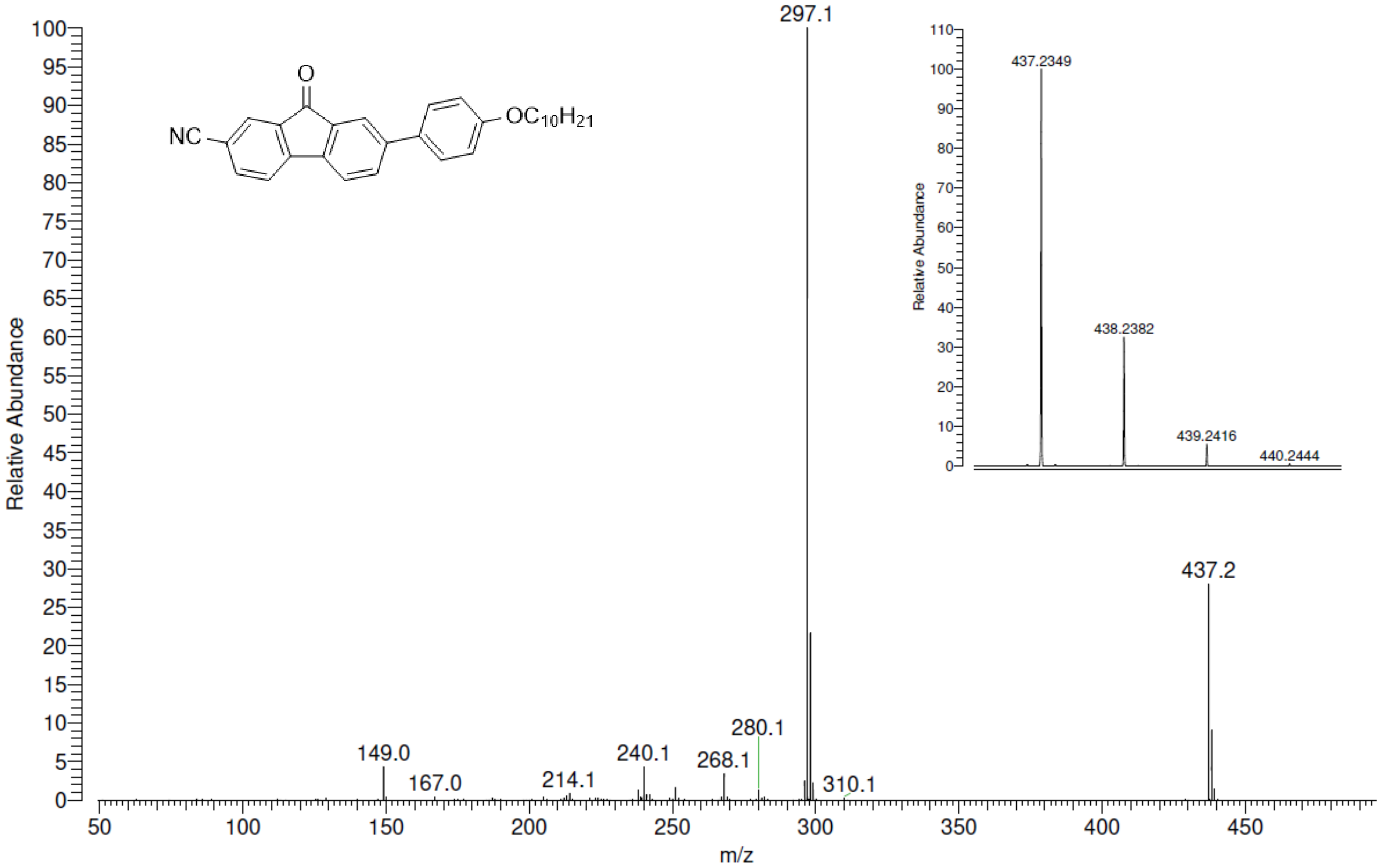

Figure S67: Mass spectrum (EI) with HRMS of **CN-FO-Ar**.

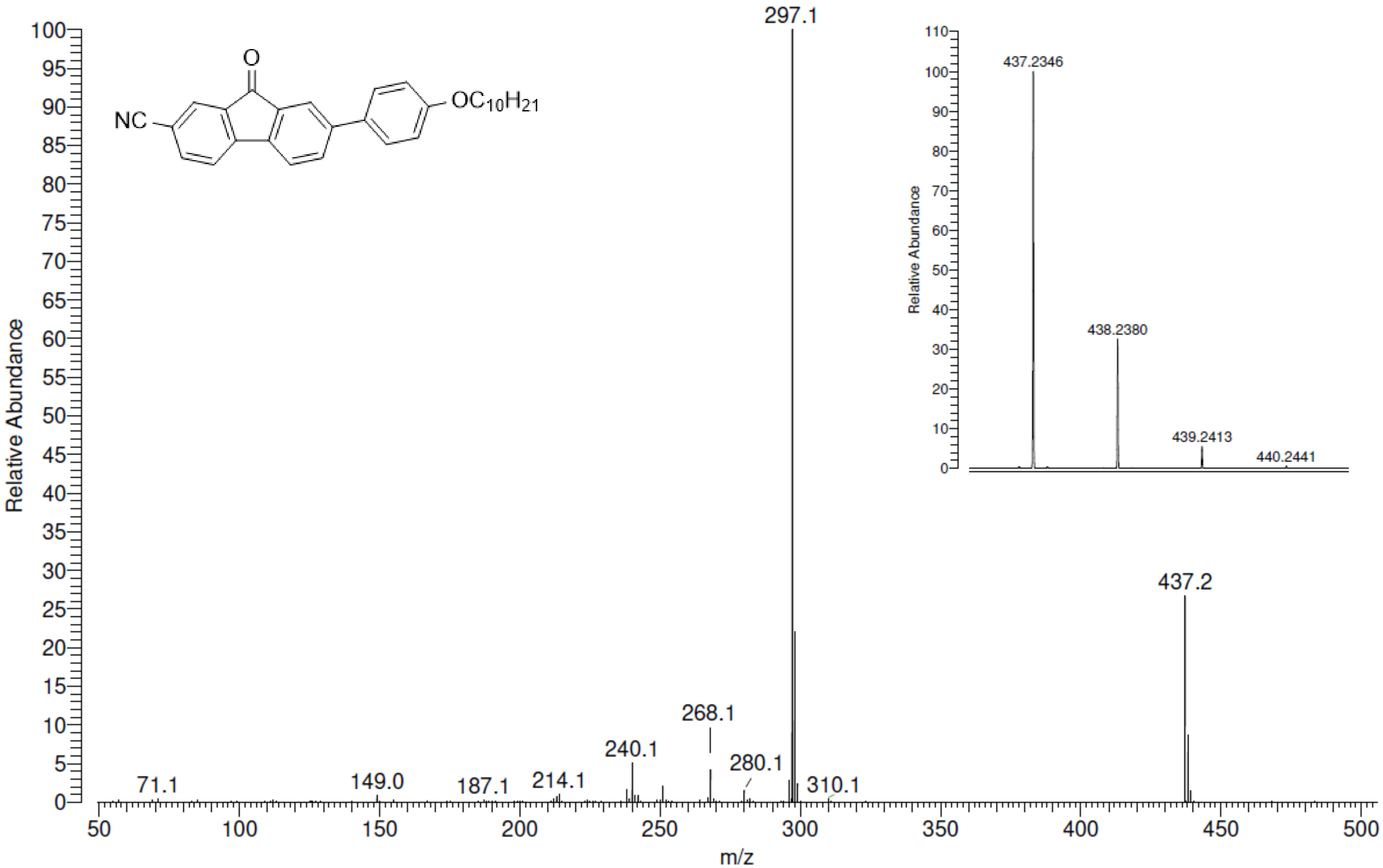

Figure S68: Mass spectrum (EI) with HRMS of **CN-FO-Ar**[h].

8. References

[1] H. E. Gottlieb, V. Kotlyar, A. Nudelman, “NMR Chemical Shifts of Common Laboratory Solvents as Trace Impurities” *J. Org. Chem.* **1997**, *62*, 7512–7515.

[2] CrysAlisPro 1.171.44.137a *Rigaku Oxford Diffraction*, **2026**

[3] O. V. Dolomanov, L. J. Bourhis, R. J. Gildea, J. A. K. Howard, H. Puschmann, “*OLEX2* : a complete structure solution, refinement and analysis program” *J. Appl. Crystallogr.* **2009**, *42*, 339–341.

[4] G. M. Sheldrick, “*SHELXT* – Integrated space-group and crystal-structure determination” *Acta Crystallogr. Sect. Found. Adv.* **2015**, *71*, 3–8.

[5] G. M. Sheldrick, “Crystal structure refinement with *SHELXL*” *Acta Crystallogr. Sect. C Struct. Chem.* **2015**, *71*, 3–8.

[6] Y. Pu, Y. Liang, X. Hu, G. Li, B. Deng, X. Liu, Y. Xiao, “The effect of terminal halogen atom on the self-assembly of bisthienylethene-based rod-like molecules” *J. Iran. Chem. Soc.* **2024**, *21*, 2489–2499.

[7] X. Zhang, J.-B. Han, P.-F. Li, X. Ji, Z. Zhang, “Improved, Highly Efficient, and Green Synthesis of Bromofluorenones and Nitrofluorenones in Water” *Synth. Commun.* **2009**, *39*, 3804–3815.

[8] W. Sharmoukh, K. C. Ko, C. Noh, J. Y. Lee, S. U. Son, “Designed Synthesis of Multi-Electrochromic Systems Bearing Diaryl Ketone and Isophthalates” *J. Org. Chem.* **2010**, *75*, 6708–6711.

[9] C. Reichardt, “Solvatochromic Dyes as Solvent Polarity Indicators” *Chem. Rev.* **1994**, *94*, 2319–2358.
